# Taxonomic revision of genus *Ablattaria* Reitter (Coleoptera, Silphidae) using geometric morphometrics

**DOI:** 10.3897/zookeys.477.8446

**Published:** 2015-01-26

**Authors:** Jarin Qubaiová, Jan Růžička, Hana Šípková

**Affiliations:** 1 Department of Ecology, Faculty of Environmental Sciences, Czech University of Life Sciences Prague, Kamýcká 129, CZ-165 21 Prague 6, Czech Republic

**Keywords:** Taxonomy, new synonymy, lectotype designation, distribution, Western Palaearctic Region

## Abstract

The genus *Ablattaria* Reitter, 1884 (Coleoptera: Silphidae: Silphinae) is revised. Four taxa are recognized as valid species: *Ablattaria arenaria* (Kraatz, 1876), *Ablattaria cribrata* (Ménétries, 1832), *Ablattaria laevigata* (Fabricius, 1775) and *Ablattaria subtriangula* Reitter, 1905. Ablattaria laevigata var. meridionalis Ganglbauer, 1899 is newly treated as a junior subjective synonym of *Ablattaria laevigata*. Lectotypes are designated for *Phosphuga arenaria* Kraatz, 1876, Ablattaria arenaria var. punctigera Reitter, 1884, Ablattaria arenaria var. alleoni Portevin, 1926, *Silpha cribrata* Ménétries, 1832, *Silpha laevigata* Fabricius, 1775, *Silpha gibba* Brullé, 1832, Ablattaria gibba var. costulata Portevin, 1926, Ablattaria gibba var. distinguenda Portevin, 1926, Ablattaria gibba var. punctata Portevin, 1926 and *Ablattaria subtriangula* Reitter, 1905. The distribution of all taxa is mapped, based on material examined. Geometric morphometric methods were used to evaluate shape variability in *Ablattaria*. Results indicated sexual dimorphism in all species. Shape inconsistency was found between the sexes of all taxa when tested independently. The first two relative warp axes indicated 65.17% shape variation in males and 65.72% in females. Canonical variate analysis separated the taxa studied. There was minimal overlap between some groups in both sexes. Differences in body shape between populations of *Ablattaria laevigata* from Central Europe, Italy and Greece + Turkey were also examined. Relative warps implied 58.01% shape variability on both axes in males and 64.78% in females. CVA revealed noticeable overlaps between the groups, although the Italian population demonstrated a higher separation in both sexes.

## Introduction

The genus *Ablattaria* Reitter, 1884 (Silphidae: Silphinae) is a specialized group of gastropod predators. Distributed in the Western Palaearctic Region, these beetles inhabit forests, gardens, scrubland and generally damp localities (Portevin 1926, Heymons and Lengerken 1932).

Reitter (1884) erected *Ablattaria* as a separate genus to accommodate five taxa of carrion beetles: the widely distributed European *Silpha laevigata* Fabricius, 1775; *Silpha gibba* Brullé, 1832 from Greece: Arcadia (originally described as separate species, but treated by Reitter as a variety of *Ablattaria laevigata*); *Silpha cribrata* Ménétries, 1832 from southern Russia; *Phosphuga arenaria* Kraatz, 1876 from Asia Minor; as well as the newly described Ablattaria arenaria var. punctigera Reitter, 1884 from Haifa. Later, Ganglbauer (1899) described Ablattaria laevigata var. meridionalis (merely as a geographic variety occurring in a large area ranging from southern Hungary to Greece) and Reitter (1905) added *Ablattaria subtriangula* from Spain. Portevin (1926) in his world revision of carrion beetles treated *Ablattaria gibba* once more as a separate species, and added several new varieties: Ablattaria arenaria var. alleoni (no distribution provided, but the type specimen is labelled as coming from Turkey: Adana), Ablattaria gibba var. costulata (type specimen from Turkey: Istanbul), Ablattaria gibba var. distinguenda and Ablattaria gibba var. punctata (type locality not specified for either taxon). *Silpha laevigata* Fabricius, 1775 is the type species of *Ablattaria* by subsequent designation by Hatch (1928), who treated *Ablattaria* as a subgenus of *Silpha* Linnaeus, 1758. Probably the broadest review of this genus was published by Schawaller (1979), who distinguished four species: *Ablattaria arenaria*, *Ablattaria cribrata*, *Ablattaria laevigata*, and *Ablattaria subtriangula*, and formally ranked two additional taxa as subspecies of *Ablattaria laevigata*: *Ablattaria laevigata gibba* (Brullé, 1832), and *Ablattaria laevigata meridionalis* Ganglbauer, 1899. Schawaller provided redescriptions of all taxa, a key to adults and a brief summary of their distributions. Recently, Nikolaev and Kozminykh (2002) treated only two taxa as full species. They regarded *Ablattaria cribrata* as a subspecies of *Ablattaria laevigata*, conditionally stated that *Ablattaria arenaria* should be considered also as a subspecies of *Ablattaria laevigata*, and formally treated *Ablattaria gibba* as a junior subjective synonym of *Ablattaria laevigata*. Most of these changes were followed in the Palaearctic catalogue by Růžička and Schneider (2004). The main morphological characters used to delimit separate species are differences in shape and surface punctation of pronotum and elytra. No consistent differences were found in the shape of male genitalia (Schawaller 1979).

Historically, most controversies have concerned the delimitations and distributions of *Ablattaria laevigata*, *Ablattaria gibba* and Ablattaria laevigata var. meridionalis (also treated at different ranks, see above). *Ablattaria laevigata* is a widely distributed European species (e.g., Portevin 1926, Schawaller 1979). Its distribution in Central Europe was given in detail by Horion (1949) for Germany and Austria and mentioned by Mroczkowski (1955) from southern Poland. *Ablattaria gibba* was originally described from southern Greece (Peloponnese Peninsula: Arcadia region) (Brullé 1832), and Ablattaria laevigata var. meridionalis was delimited as coming from “Illiria, Dalmatia, southern Hungary and Greece” (Ganglbauer 1899). However, later authors confused the distributions of the two taxa: Porta (1926) treated Ablattaria laevigata var. gibba from “Lombardia, Veneto, Toscana, Lazio, southern Italy” and Ablattaria laevigata var. meridionalis from “Corsica”. Portevin (1926) reported *Ablattaria gibba* from Romania, Greece and Anatolia and Ablattaria laevigata var. meridionalis from “southern Europe”. Hatch (1928) repeated Portevin’s distribution data for *Ablattaria gibba* as “Rumania [sic], Greece, Anatolia” and added a record for Ablattaria laevigata var. meridionalis from “Eastern Europe”. Schawaller (1979) reported *Ablattaria laevigata laevigata* from the south of Central Europe and from France and Spain, *Ablattaria laevigata gibba* from the Balkan Peninsula to central Anatolia, and *Ablattaria laevigata meridionalis* from Italy, including the surrounding islands.

The genus *Ablattaria* was further reported from many regions: Iberian Peninsula (Caminero Bago 1981, Piloña et al. 2002), France (Debreuil 2004), Central Europe (Růžička 2005), Bulgaria (Guéorguiev and Růžička 2002), Iran and Turkey (Růžička 1996, Háva et al. 1998, Růžička and Schneider 2002), Russia, Ukraine and the Caucasus (Nikolaev and Kozminykh 2002).

The ecology and detailed adult and larval morphology of *Ablattaria laevigata* were described in detail by Heymons and Lengerken (1932). Colkesen and Sekeroglu (1989) examined the development and biology of *Ablattaria arenaria* adults and larvae. Further, Sekeroglu and Colkesen (1989) studied the feeding and prey preferences of *Ablattaria arenaria* larvae.

In this study, we revise the taxonomy of the genus. We provide new lectotype designations and synonymies based on morphological characters and using the valuable technique of geometric morphometrics on the adult beetle’s body shape. These methods helped us to distinguish taxa and understand variation within and between populations. Based on the material examined, we further summarize information about the precise distribution of the taxa.

## Materials and methods

Overall, 2729 specimens were examined from various European museums and collections with acronyms as follow:

BMNH Natural History Museum, London, United Kingdom (M.V.L. Barclay)

EHOC Private collection of Erwin Holzer, Anger, Austria

HNHM Magyar Természettudományi Museum, Budapest, Hungary (O. Merkl)

JCOC Private collection of Jonathan Cooter, Hereford, United Kingdom

JRUC Private collection of Jan Růžička, Prague, Czech Republic

KORC Private collection of Kamil Orszulik, Frýdek-Místek, Czech Republic

MHNG Museum d’histoire naturelle, Genève, Switzerland (G. Cuccodoro)

MNHN Muséum national d’Histoire naturelle, Paris, France (Azadeh Taghavian)

MNCN Museo Nacional de Ciencias Naturales, Madrid, Spain (J.F. Gómez)

MZMB Moravské zemské muzeum, Brno, Czech Republic (I. Malenovský)

NHMW Naturhistorisches Museum, Vienna, Austria (H. Schillhammer)

NJAC Private collection of Nicklas Jansson, Linköping, Sweden

NMPC Národní muzeum, Prague, Czech Republic (J. Hájek)

SDEI Senckenberg Deutsche Entomologische Institut, Müncheberg, Germany (L. Zerche, L. Behne)

SMFD Forschungsinstitut Senckenberg, Frankfurt am Main, Germany (D. Kovac)

SMNS Staatliches Museum für Naturkunde, Stuttgart, Germany (W. Schawaller)

SMTD Staatliches Museum für Tierkunde, Dresden, Germany (O. Jäger)

TAUM Department of Zoology, Tel Aviv University, Tel Aviv, Israel (V. Chikatunov)

TSIC Private collection of Tomáš Sitek, Ostrava, Czech Republic

ZFMK Forschungsmuseum Alexander Koenig, Bonn, Germany (D. Ahrens)

ZMAN Zoölogisch Museum Amsterdam, Amsterdam, the Netherlands (S.A. Ulenberg)

ZMAS Zoological Museum, Academy of Sciences, St. Petersburg, Russia (M.G. Volkovich);

ZMHB Museum für Naturkunde – Leibniz-Institut für Evolutions- und Biodiversitätsforschung an der Humboldt-Universität zu Berlin, Berlin, Germany (J. Frisch);

ZMUC Zoological Museum, University of Copenhagen, Copenhagen, Denmark (A. Solodovnikov)

ZMUM Zoological Museum of Moscow Lomonosov State University, Moscow, Russia (N. Nikitsky)

ZSM Zoologische Staatssammlung, Munich, Germany (M. Balke)

Types of most taxa were located and examined. Lectotypes for most taxa are designated below to fix the concept of the taxon in question and to ensure its universal and consistent application and interpretation.

### Morphological analyses

Photographs of habitus and morphological details were taken using a Canon MP-E 65 mm or EF-S 60 mm macro photo lens on a Canon 550D, and several layers of focus combined in Zerene Stacker 1.04 software (Zerene Systems 2014; http://www.zerenesystems.com/cms/stacker). Exact label data of primary types were cited verbatim. Separate lines on labels are indicated by a slash “/”, separate labels by double slash “//”. Author’s remarks and comments are enclosed in square brackets. The following abbreviations are used: p – preceding data are printed; hw – preceding data are hand-written. Interpreted label data of non-type material examined is summarized in Appendix 1. Data are available from the Dryad Digital Repository (http://doi.org/10.5061/dryad.7dn7m). To determine the coordinates of the localities, Google Earth (2014; http://earth.google.com) was used along with maplandia (http://www.maplandia.com). Distributional maps were created in ESRI ArcMap 10.2 of ArcGIS Desktop 10.2 suite. For map layers, free level 0 data from Global Administrative Areas (http://www.gadm.org) and World Shaded Relief (http://www.arcgis.com/home/item.html?id=9c5370d0b54f4de1b48a3792d7377ff2) were used.

### Geometric morphometrics

Four species of the genus *Ablattaria* were examined: *Ablattaria laevigata* (145 males, 174 females), *Ablattaria arenaria* (85 males, 87 females), *Ablattaria cribrata* (49 males, 33 females) and *Ablattaria subtriangula* (5 males, 8 females). Moreover, three groups representing populations of *Ablattaria laevigata* were tested: one population from Greece and Turkey (26 males, 37 females), a population from Italy (39 males, 33 females), and one from Central Europe (Austria, Hungary and one specimen from the Czech Republic) (35 males, 33 females). Images were captured using an Olympus digital reflex camera (model E-330) connected to an Olympus stereoscopic microscope (model SZX7) and combined body length of pronotum and elytra was measured.

The geometric morphometric analysis was performed using the thin-plate spline (TPS) package; available free at http://life.bio.sunysb.edu/morph/index.html (Rohlf 2014). This technique utilizes coordinates of specific locations called landmarks that are precise points on each specimen describing the overall shape and representing the specimen’s morphology (Bookstein 1982, 1986, 1989 and 1991).

In TpsDig 2.10 (Rohlf 2006) the “draw background curves” tool was employed to digitize a curve that outlined only the left half of the pronotum and the left elytron formed from 55 points. The homology of these points on all samples and their reliability in demonstrating the highest shape variability was considered (Bookstein 1991, Slice 2007). The curve points were converted into landmarks using TpsUtil 1.44 (Rohlf 2009) for further analysis.

Landmarks were then superimposed by generalized Procrustes analysis, which allows calculating variability between the taxa after aligning their landmark configurations in a specific process that ensures homology (Rohlf 1990, Rohlf and Slice 1990, Rohlf and Marcus 1993, Zelditch et al. 1995). This was conducted in TpsRelw 1.53 (Rohlf 2013). Relative warp analysis was also performed, wherein the relative warps (RWs) are transformations that express the patterns of shape variation among the specimens and visualize it using D’Arcy Thompson’s transformation grids. The deformations in the grids represent the shape changes (Rohlf 1993, Richtsmeier et al. 2002, Adams et al. 2004, Zelditch et al. 2012).

Multivariate analysis of variance (MANOVA) and discriminant analysis (DA) were applied on the relative warp scores matrix to test the significance of the variations between groups (taxa/sexes), and canonical variate analysis (CVA) was performed to illustrate these differences (Zelditch et al. 2004, 2012). Graphical visualization of the CVA results was also made. All of the preceding analyses were executed in PAST ver. 2.11; freeware available for download at http://folk.uio.no/ohammer/past/ (Hammer et al. 2001).

Geometric morphometrics employs centroid size rather than linear size in calculations associated with allometry (which is the influence size has on shape) (Bookstein 1991; Klingenberg 2010, Zelditch et al. 2004, 2012). The natural logarithm of centroid size was used here, as it increases the statistical power (Viscoci and Cardini 2011). The taxon groups were first tested independently. Furthermore, multivariate analysis of covariance (MANCOVA) was used in the size correction when comparing groups to test its effect on body shape. In this analysis, the log of centroid size was used as the covariate. TpsRegr 1.38 (Rohlf 2011) was applied to calculate this influence and run permutation tests (Rohlf 1998, Viscoci and Cardini 2011, Zelditch et al. 2012).

## Taxonomy

### Ablattaria

Taxon classificationAnimaliaColeopteraSilphidae

Reitter, 1884

Ablattaria Reitter 1884: 75.

#### Type species.

*Silpha laevigata* Fabricius, 1775 (subsequently designated by Hatch (1928: 120)).

#### Diagnostic description.

Body, in general, dull-black (brown to dark brown in subteneral specimens), total body length 9–19 mm.

Head flattened with dense but fine puncturing, extra prolonged (used for the invasion of snail shells during feeding; Fig. 20). Eyes large, prominent, emerge to the sides. Antennae clavate, club formed by the antennomeres 9–11 (Fig. 22). Antennomere 1 longer than antennomeres 2 and 3 combined. Antennomere 2 slightly longer than antennomere 3. Frons broad, mandibles large and sickle-shaped, typical to snail eaters, maxilla densely haired outwards (Fig. 20).

Pronotum with continuous margins, semielliptical (Figs 12, 13, 15) (conical in *Ablattaria subtriangula*, Fig. 14), with distinct punctures covering its dorsal surface (Figs 13–15) (only very superficial medially in *Ablattaria arenaria*, Fig. 12), rarely with a fine line in the middle.

Scutellar shield small in size, cordiform in shape and with distinct punctation.

Elytra regularly vaulted, densely and regularly punctured (Figs 16–19), without vestigial ribs, rarely with two very fine, longitudinal lines that are occasionally more visible (Fig. 21). Elytral epipleural ridge is incomplete; extends along the elytron but not to its subapical part (Figs 9–11). Punctures homogenously distributed, of similar size (Figs 18, 19) or varying in size, fine punctures intermixed with larger ones, predominantly in medial part (Figs 16, 17).

Legs strong with fine spines, femur of hind legs broad, tibia ends with an apical spine stretching out (Figs 5, 6). Tarsi with robust tarsal claws. Males with laterally expanding tarsomeres, females with cylindrical and more slender tarsomeres (e.g., as show in Figs 4 and 5).

**Figures 1–4.**
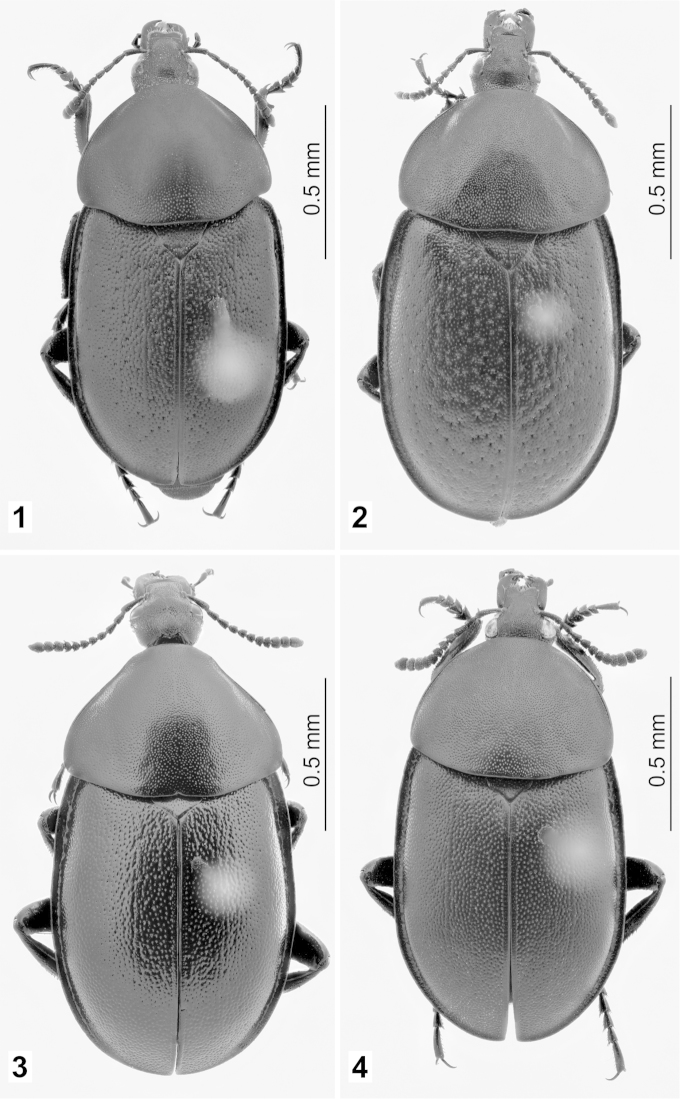
Habitus in dorsal view: **1** *Ablattaria arenaria* (male, Israel: Mount Carmel) **2** *Ablattaria cribrata* (female, Azerbaijan: Zagulba Baglari) **3** *Ablattaria subtriangula* (female, Spain: Cameros) **4** *Ablattaria laevigata* (male, Hungary: Budapest).

**Figures 5–8.**
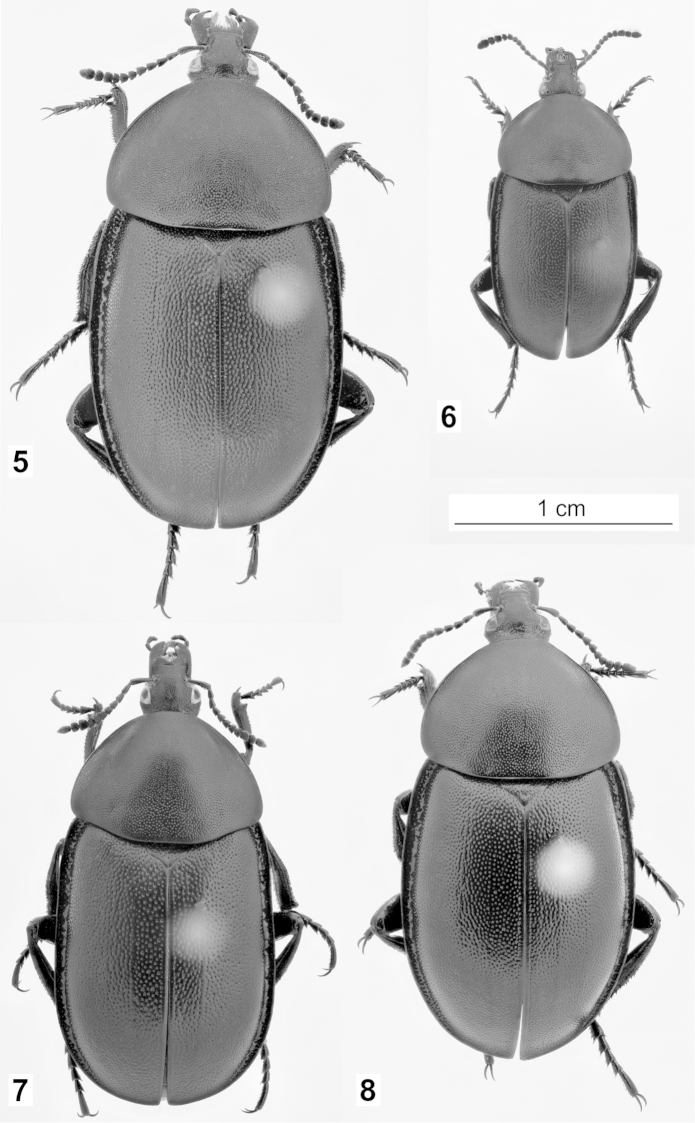
Habitus of *Ablattaria laevigata* in dorsal view: **5** female (Croatia: Pula) **6** male (Austria: Elenderwald) **7** female (Greece: Loutraki) **8** female (Italy: Pioppi).

**Figures 9–11.**
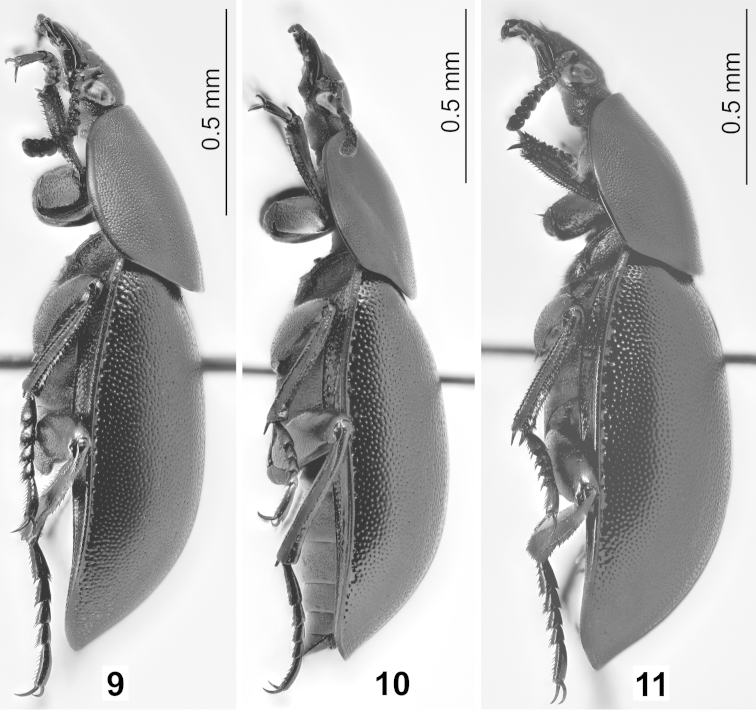
Habitus of *Ablattaria laevigata* in dorsal view: **9** male (Hungary: Budapest) **10** female (Greece: Loutraki) **11** female (Italy: Pioppi).

**Figures 12–15.**
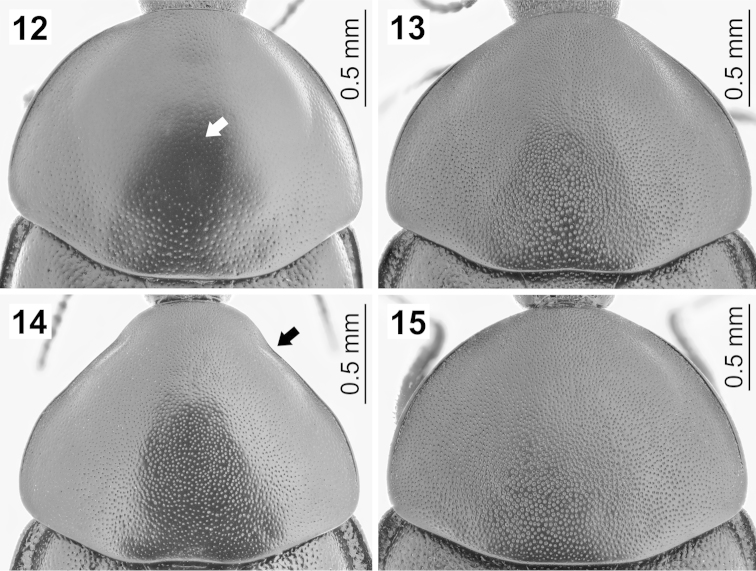
Pronotum in dorsal view: **12** *Ablattaria arenaria* (female, Iraq: Khanaqin) **13** *Ablattaria cribrata* (male, Russia: Dagestan) **14** *Ablattaria subtriangula* (male, Spain: Soto) **15** *Ablattaria laevigata* (female, Austria: Bisamberg).

**Figures 16–19.**
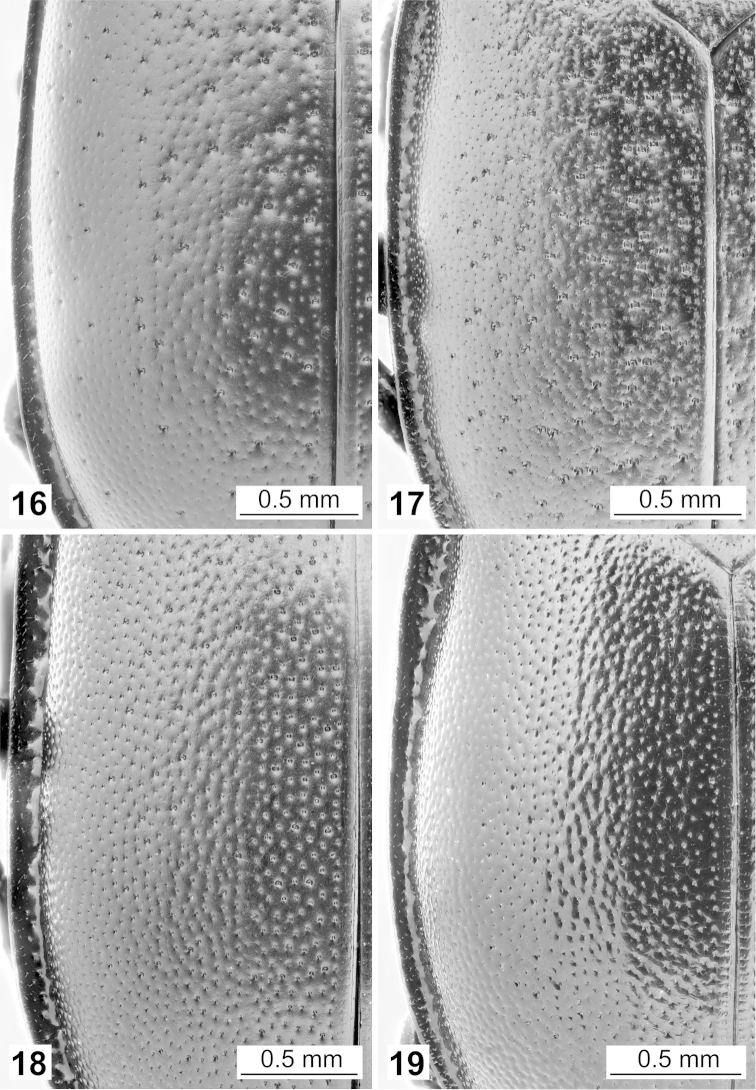
Left elytron in dorsal view: **16** *Ablattaria arenaria* (female, Iraq: Khanaqin) **17** *Ablattaria cribrata* (male, Russia: Dagestan) **18** *Ablattaria laevigata* (female, Austria: Bisamberg) **19** *Ablattaria subtriangula* (male, Spain: Soto).

**Figures 20–22.**
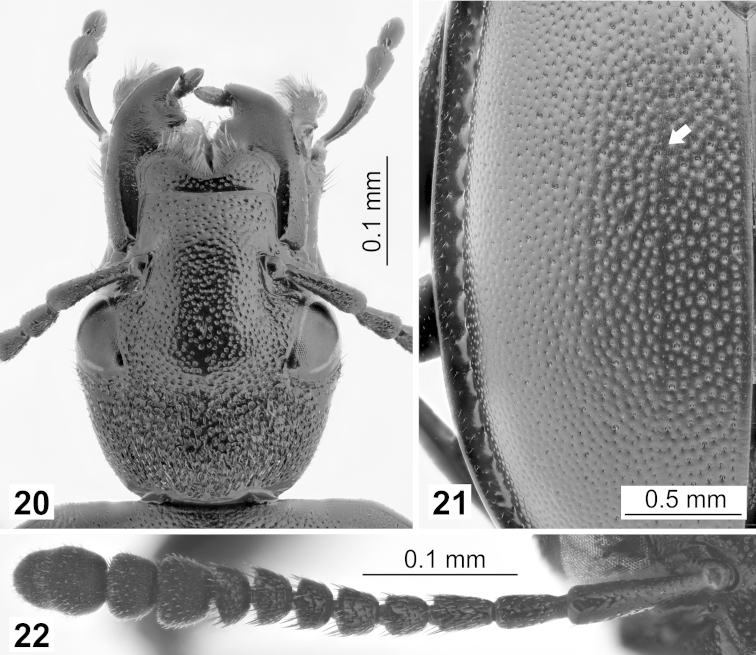
Morphological details in dorsal view: **20** *Ablattaria subtriangula*, elongated head (female, Spain: Cameros) **21** *Ablattaria laevigata*, variable left elytron with traces of two lines (male, Greece: Alistrati) **22** *Ablattaria laevigata*, right antenna (female, Hungary: Budapest).

#### Phylogenetic position.

*Ablattaria* is classified preliminarily as a sister lineage to *Phosphuga* Leach, 1817 and *Silpha* Linnaeus, 1758, based on 2.1 kB sequence of cytochrome oxidase subunits I and II (Dobler and Müller 2000, Sikes et al. 2005), sometimes treated also as a subgenus of *Silpha* (Sikes et al. 2005).

#### Key to the *Ablattaria* species

**Table:** 

1	Elytra with medium-sized, distinct punctures of similar size (Figs 18, 19)	**2**
–	Elytra with fine punctures, intermixed with larger ones (Figs 16, 17)	**3**
2	Pronotum semi-elliptical, margin regularly rounded anteriorly (Fig. 15), surface matt (Fig. 4). Widely distributed from western and southern Europe (incl. Spain) to southern Russia, western and northern part of Asia Minor and western Transcaucasia	***Ablattaria laevigata***
–	Pronotum almost conical in shape, margins anterolaterally constricted (Fig. 14, arrow), surface glossy (Fig. 3). Endemic to Spain	***Ablattaria subtriangula***
3	Pronotum with only very superficial, very fine punctures medially on disc (which looks impunctate under lower magnification), much larger punctures more peripherally (Fig. 12). Elytra with few larger punctures (usually slightly finer than in *Ablattaria cribrata*), dispersed mostly toward the inner elytral margin (Fig. 16). Eastern part of Greece, Asia Minor, Middle East	***Ablattaria arenaria***
–	Pronotum with evenly distributed, homogenous, distinct punctures on whole dorsal surface (Fig. 13). Elytra with middle-sized, more densely arranged larger punctures, very dense toward the inner elytral margin and here sometimes subquadrate in shape (Fig. 17). Caucasus, Transcaucasia, Iran, south-western Turkmenistan	***Ablattaria cribrata***

### Ablattaria arenaria

Taxon classificationAnimaliaColeopteraSilphidae

(Kraatz, 1876)

**Figures 23–27.**
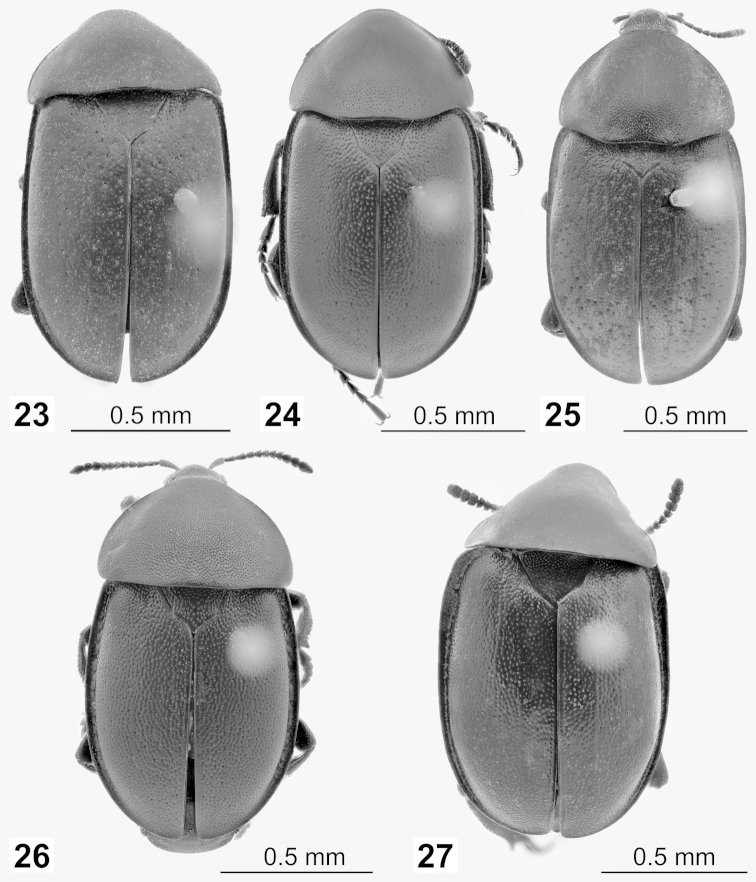
**23–26** Lectotypes of *Ablattaria* spp. in dorsal view: **23** *Phosphuga arenaria* Kraatz **24** Ablattaria arenaria var. punctigera Reitter **25** *Silpha cribrata* Ménétries **26** Ablattaria gibba var. punctata Portevin **27** *Ablattaria subtriangula* Reitter, paralectotype.

Figs 1 , 12 , 16 , 23 , 24

Phosphuga arenaria Kraatz 1876: 368 (type locality: “Creta, Anatolien” [= Crete, Anatolia]).
Ablattaria arenaria var. punctigera Reitter 1884: 75 (type locality: “Haifa” [ca. 32°49'N 34°59'E]) (as junior synonym of *Ablattaria arenaria* by Schawaller 1979: 6).
Ablattaria arenaria var. Alleoni Portevin 1926: 24 (type locality not stated) (as junior synonym of *Ablattaria arenaria* by Schawaller 1979: 6).

#### Type material examined.

Lectotype male of *Phosphuga arenaria* (here designated) (SDEI, general collection) (Fig. 23), pinned, labelled: “866 [hw, black frame] // coll. Kraatz [p] // Typus [p, red label] // arenaria / Kraatz Küst. XXX / Asia minor [hw, Kraatz’s handwriting, light yellow label with black frame] // coll. SDEI / (Müncheberg) / general coll. [p, light green label] // Lectotype / Phosphuga arenaria / Kraatz, 1876 / J. Qubaiová & J. Růžička / des. 2014 [p, red label] // Ablattaria / arenaria (Kraatz, 1876) / J. Qubaiová & J. Růžička / det. 2014 [p]”.

Lectotype male of Ablattaria arenaria var. alleoni (here designated) (MNHN, coll. Pic), pinned, labelled: “Adana [hw] // var. Alleoni / m. [hw, Portevin’s handwriting] // Type [p, red label with black frame] // Muséum Paris / Coll. M. Pic [p] // Lectotype / Ablattaria arenaria / var. alleoni Portevin, 1926 / J. Qubaiová & J. Růžička / des. 2014 [p, red label] // Ablattaria / arenaria (Kraatz, 1876) / J. Qubaiová & J. Růžička / det. 2014 [p]”.

Lectotype male of Ablattaria arenaria var. punctigera (here designated) (HNHM) (Fig. 24), pinned, labelled: “Syrien / Haifa / Reitter [p, black frame] // coll. Reitter [p] // Holotypus [p, red letters] 1884 / Ablattaria arenaria / v. punctigera / Reitter [hw, thick red frame; subsequent label added probably by Z. Kaszab or V. Székessy (O. Merkl, pers. comm.)] // Lectotype / Ablattaria arenaria / var. punctigera / Reitter, 1884 / J. Qubaiová & J. Růžička / des. 2014 [p, red label] // Ablattaria / arenaria (Kraatz, 1876) / J. Qubaiová & J. Růžička det. 2014 [p]”. Paralectotypes: 1 male, 2 females (HNHM), pinned, with identical labels as lectotype except for “Paratypus [p, red letters] 1884 / Ablattaria arenaria / v. punctigera / Reitter [hw, thick red frame] // Paralectotype / Ablattaria arenaria / var. punctigera / Reitter, 1884 / J. Qubaiová & J. Růžička / des. 2014 [p, red label]”; 1 male (SMTD), pinned, with identical labels as previous paralectotypes except for “Staatl. Museum für / Tierkunde Dresden [p]”; 1 male (SDEI, coll. Heyden), pinned, labelled: “Haifa Syriae [p] // Simon. [p] // Syntypus [p, red label] // arenaria / v. puncti- / gera m. [hw, Reitter’s handwriting] // coll. SDEI / (Müncheberg) / coll. HEYDEN [p, modern light green label] // Paralectotype / Ablattaria arenaria / var. punctigera / Reitter, 1884 / J. Qubaiová & J. Růžička / des. 2014 [p, red label] // Ablattaria / arenaria (Kraatz, 1876) / J. Qubaiová & J. Růžička / det. 2014 [p]”; 1 female (MNHN, general collection), pinned, labelled: “Syrien / Haifa / Reitter [p] // arenaria / Kratz [hw] // arenaria / v. punctigera [hw, Reitter’s handwriting; yellow label] // 249 // MUSEUM PARIS / Coll. A. GROUVELLE 1917 [p] // Paralectotype / Ablattaria arenaria / var. punctigera / Reitter, 1884 / J. Qubaiová & J. Růžička / des. 2014 [p, red label] // Ablattaria / arenaria (Kraatz, 1876) / J. Qubaiová & J. Růžička / det. 2014 [p]”.

#### Additional material examined.

391 specimens, see Appendix 1.

#### Diagnostic description.

Total body length 11–15 mm, body matt. Pronotum semi-elliptical, with only very superficial, very fine punctures medially on disc (which looks impunctate under lower magnification), much larger punctures more peripherally (Fig. 12). Elytra with fine punctures that are finer in size and less close together than in *Ablattaria cribrata* and *Ablattaria laevigata*, with few intermixed larger punctures dispersed mostly toward the inner elytral margin (Fig. 16).

#### Remarks.

Subtle differences in punctation of the scutellar shield and elytra, which differentiate the two varieties described by Reitter (1884) and Portevin (1926), fall within the intraspecific variability of *Ablattaria arenaria*. We confirm their status as junior subjective synonyms, as already proposed by Schawaller (1979).

#### Biology.

Seasonal activity of adults with a peak in March–May (Table 1).

**Table 1.** Seasonal activity of *Ablattaria* spp. (based on number of examined specimens per month). *Ablattaria subtriangula* is excluded, due to lack of material with precise seasonality data.

Species / month	1	2	3	4	5	6	7	8	9	10	11	12
*Ablattaria arenaria*	1	11	24	68	31	19	5	1	1	6	0	2
*Ablattaria cribrata*	0	0	1	6	13	13	7	5	4	0	0	2
*Ablattaria laevigata*	1	7	50	222	235	154	88	71	33	33	1	1

#### Distribution.

Greece (only Crete and Rhodes), Cyprus, Turkey, to the south of Iraq, Israel, Jordan, Lebanon, Syria and the south-west of Iran (Fig. 32).

### Ablattaria cribrata

Taxon classificationAnimaliaColeopteraSilphidae

(Ménétries, 1832)

Figs 2 , 13 , 17

Silpha cribrata Ménétries 1832: 168 (attributed to Faldermann) (type locality: “Derbent” [ca. 42°03'N, 48°18'E]).
Silpha cribrata Faldermann 1835: 221 (type locality: “Rossia” [= Russia]; preoccupied, not Ménétries 1832: 168 (junior primary homonym)).

#### Type material examined.

Lectotype female of *Silpha cribrata* Ménétries (here designated) (MNHN, general collection) (Fig. 25), pinned, subteneral, labelled: “[female sign, hw] // Ménétr. [hw] // TYPE [p, modern red label] // Cribrata / Faldermann / Caucase [hw, probably Ménétries’s handwriting, yellow label with partial black frame] // MUSEUM PARIS / Coll. A. GROUVELLE 1915 [p] // Lectotype / Silpha cribrata / Ménétries, 1832 / J. Qubaiová & J. Růžička / des. 2014 [p, red label] // Ablattaria / cribrata (Ménétries, 1832) / J. Qubaiová & J. Růžička / det. 2014 [p]”. Paralectotypes: 1 male (ZMAS), pinned, labelled: “[golden-black quadrate label] // Caucasus [p, pink label] // cribrata / Fald. Russ. mer. [hw, double black frame] // Paralectotype / Silpha cribrata / Ménétries, 1832 / J. Qubaiová & J. Růžička / des. 2014 [p, red label] // Ablattaria / cribrata (Ménétries, 1832) / J. Qubaiová & J. Růžička / det. 2014 [p]”; 1 specimen (sex not identified) (ZMHB), pinned, badly damaged (only part of meso- and metathorax with right elytron and left meso- and metaleg present), labelled: “Type. [p] // Elliposilpha / cribrata / Caucas Ménétr [hw, Motschulsky’s handwriting] // Museum für Naturkunde / Humboldt-Univ. Berlin / (MNHUB) [p, modern label] // [large red label] // Paralectotype / Silpha cribrata / Ménétries, 1832 / J. Qubaiová & J. Růžička / des. 2014 [p, red label] // Ablattaria / cribrata (Ménétries, 1832) / J. Qubaiová & J. Růžička / det. 2014 [p]”.

#### Additional material examined.

95 specimens, see Appendix 1.

#### Diagnostic description.

Total body length 11–16 mm, body matt. Pronotum semi-elliptical, with evenly distributed, homogenous, distinct punctures on whole dorsal surface (Fig. 13). Elytra more flattened; with middle-sized, more densely arranged larger punctures, very dense toward the inner elytral margin and here sometimes subquadrate in shape (Fig. 17).

Elytra more flattened. Large punctures are dispersed over the entire elytra with a higher concentration towards the inner elytral margin, which makes the elytra appear coarse. Smaller punctures are also present on both elytra, scutellum and pronotum, although they appear to be larger than those of *Ablattaria laevigata* but less frequent.

#### Biology.

Seasonal activity of adults with a peak in May and June (Table 1).

#### Distribution.

From the south of Russia (Dagestan, Chechnya), Georgia, Azerbaijan, Armenia, Iran to south-western Turkmenistan (Fig. 32).

### Ablattaria laevigata

Taxon classificationAnimaliaColeopteraSilphidae

(Fabricius, 1775)

Figs 4 , 5–8 , 9–11 , 15 , 18 , 21 , 22 , 26 , 28–31

Silpha laevigata Fabricius 1775: 74 (with reference to Geoffroy (1762)) (type locality: Europe [“Habitat in sylvis Europae”]).
Silpha polita Sulzer 1776: 28 (with reference to Geoffroy (1762)) (type locality: “Schweiz” [Switzerland]; preoccupied, not Fueßli 1775: 6 (junior primary homonym)) (as junior synonym of *Ablattaria laevigata laevigata* by Reitter 1884: 75, confirmed by Schawaller 1979: 5).
Silpha gibba Brullé 1832: 162 (type locality: “Arcadie” [= Greece: Peloponnese Peninsula, Arcadia region]) (as junior synonym of *Ablattaria laevigata* by Nikolaev and Kozminykh 2002: 70).
Ablattaria laevigata gibba : Schawaller 1979: 5.
Ablattaria gibba var. costulata Portevin 1926: 25 (type locality: Istanbul [ca. 41°01'N, 28°57'E]) (as junior synonym of *Ablattaria laevigata gibba* by Schawaller 1979: 5).
Ablattaria gibba var. distinguenda Portevin 1926: 25 (type locality not stated) (as junior synonym of *Ablattaria laevigata gibba* by Schawaller 1979: 5).
Ablattaria gibba var. punctata Portevin 1926: 26 (type locality not stated) (as junior synonym of *Ablattaria laevigata gibba* by Schawaller 1979: 5).
Ablattaria laevigata var. meridionalis Ganglbauer 1899: 191 (type locality: “Illirien, Dalmatien, Südungarn und Griechenland” [= Illiria, Dalmatia, southern Hungary and Greece], **syn. n.**
Ablattaria laevigata meridionalis : Schawaller 1979: 5.

#### Type material examined.

**Figures 28–31.**
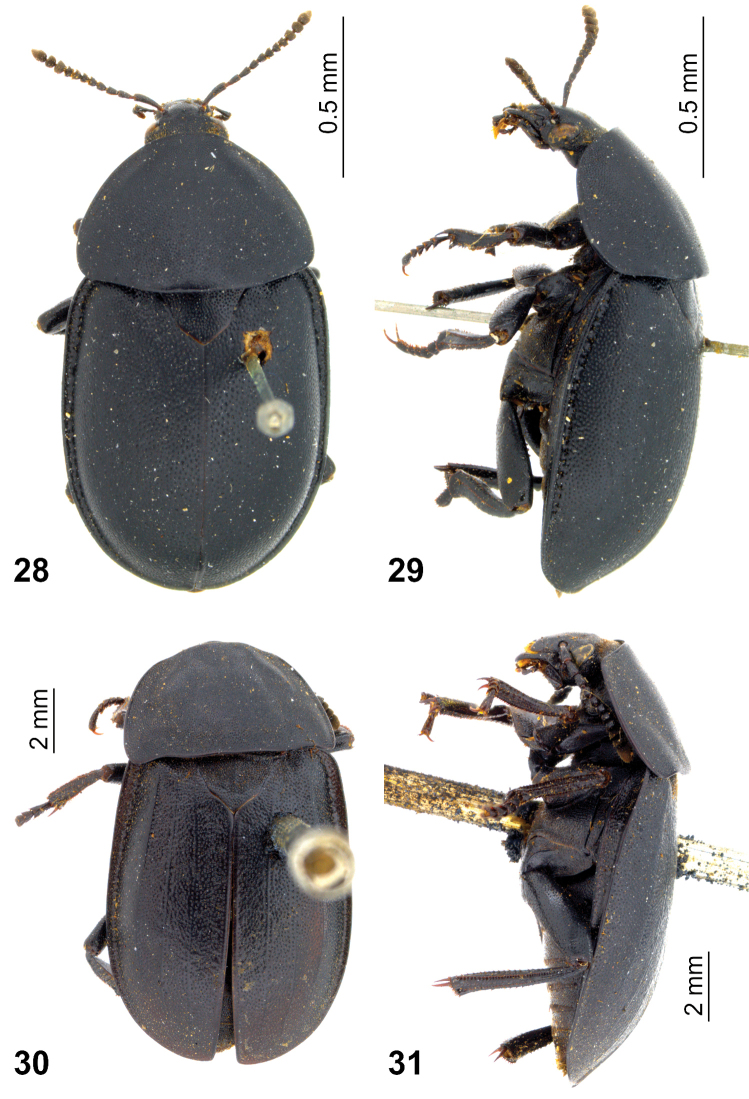
Lectotype and paralectotype of *Silpha laevigata* Fabricius: **28, 29** lectotype (female) **30, 31** paralectotype (female, *Silpha tyrolensis*; see text) **28, 30** dorsal view **29, 31** lateral view. (Photo K.P. Puliafico).

Lectotype female of *Silpha laevigata* (here designated) (ZMUC, Collection of Ove Ramel Sehested and Niels Tønder Lund, the “Copenhagen collection” [= coll. S & TL]) (Figs 28–29), pinned, labelled: “Lectotype / Silpha / laevigata / Fabricius, 1775 / Jan Růžička des. 2012 [p, red label] // Ablattaria / laevigata / (Fabricius, 1775) / Jan Růžička det. 20 [p] 12 [hw] // zmuc / 00021148 [p, this and subsequent numbers below are associated with photodocumentation]”. Paralectotypes: 1 male (ZMUC, coll. S & TL), pinned, labelled “Paralectotype / Silpha / laevigata / Fabricius, 1775 / Jan Růžička des. 2012 [p, red label] // Ablattaria / laevigata / (Fabricius, 1775) / Jan Růžička det. 20 [p] 12 [hw] // zmuc / 00021149 [p]”; 1 male and 1 female (ZMUC, Fabricius personal collection, the “Kiel collection”), pinned, identical labels as previous except for “laeviga / ta [hw, Fabricius’s handwriting, label pinned in box left to the first specimen]” and “zmuc00021468 [p]” or “zmuc00021469 [p]”; 1 female of *Silpha tyrolensis* Laicharting, 1781 (ZMUC, “Kiel collection”) (Figs 30–31), pinned, labelled “Paralectotype / Silpha / laevigata / Fabricius, 1775 / Jan Růžička des. 2012 [p, red label] // Silpha / tyrolensis / Laicharting, 1781 / Jan Růžička det. 20 [p] 12 [hw] // zmuc00021467 [p]”; 1 male (BMNH, coll. Banks), pinned, labelled: “HOLO- / TYPE [p, modern round label with thick red margin] // Silpha laevigata [hw] / Fab. Entom. p. [p] 74. n / 10 [hw, double black frame] // Paralectotype / Silpha / laevigata / Fabricius, 1775 / Jan Růžička des. 2012 [p, red label] // Ablattaria / laevigata / (Fabricius, 1775) / Jan Růžička det. 20 [p] 12 [hw]”.

Lectotype female of *Silpha gibba* (here designated) (MNHN, coll. generale), labelled: “Type / de Brullé [hw] // TYPE [p, red label] // MUSEUM PARIS / MORÉE / BRULLÉ 4187-33 [p] // Lectotype / Silpha gibba / Brullé, 1832 / J. Qubaiová & J. Růžička / des. 2014 [p, red label] // Ablattaria / laevigata (Fabricius, 1775) / J. Qubaiová & J. Růžička det. 2014 [p]”. Paralectotype: 1 male (MNHN, coll. generale), labelled: “gibba Br. [hw] // TYPE [p, red label] // MUSEUM PARIS / MORÉE / BRULLÉ 4187-33 [p] // Paralectotype Silpha gibba / Brullé, 1832 / J. Qubaiová & J. Růžička des. 2014 [p, red label] // Ablattaria / laevigata (Fabricius, 1775) / J. Qubaiová & J. Růžička det. 2014 [p]”.

Lectotype female of Ablattaria gibba var. costulata (here designated) (MNHN, coll. Pic), labelled “TURQUIE / Constantinople [= Istanbul] [p] // var. costatula [sic] / m. [hw, Portevin’s handwriting] // TYPE [p, red label] // Muséum Paris / Coll. M. Pic [p] // Lectotype / Ablattaria gibba / var. costulata / Portevin, 1926 / J. Qubaiová & J. Růžička / des. 2014 [p, red label] // Ablattaria / laevigata (Fabricius, 1775) / J. Qubaiová & J. Růžička / det. 2014 [p]”.

Lectotype male of Ablattaria gibba var. distinguenda (here designated) (MNHN, coll. Pic), pinned, labelled: “laevigata / var. [hw] // var. distinguenda / m. [hw, Portevin’s handwriting] // TYPE [p, red modern label] // Muséum Paris / Coll. M. Pic [p] // Lectotype / Ablattaria gibba / var. distinguenda / Portevin, 1926 / J. Qubaiová & J. Růžička / des. 2014 [p, red label] // Ablattaria / laevigata (Fabricius, 1775) / J. Qubaiová & J. Růžička det. 2014 [p]”.

Lectotype male of Ablattaria gibba var. punctata (here designated) (MNHN, coll. Marmottan) (Fig. 26), pinned, labelled: “Turquie [hw] // TYPE [p, red modern label] // var. punctata / m. [hw, probably Portevin’s handwriting] // Muséum Paris / 1914 / Coll. H. Marmottan [p, modern label] // Lectotype / Ablattaria gibba / var. punctata / Portevin, 1926 / J. Qubaiová & J. Růžička / des. 2014 [p, red label] // Ablattaria / laevigata (Fabricius, 1775) / J. Qubaiová & J. Růžička / det. 2014 [p]”. Paralectotype: 1 male, labelled: “MUSEUM PARIS [p] / Turquie / Jejeune 1881 [hw] // TYPE [p, red label] // Paralectotype / Ablattaria gibba var. punctata / Portevin, 1926 / J. Qubaiová & J. Růžička des. 2014 [p, red label] // Ablattaria / laevigata (Fabricius, 1775) / J. Qubaiová & J. Růžička det. 2014 [p]”.

#### Additional material examined.

2206 specimens, see Appendix 1.

#### Diagnostic description.

Total body length 9–19 mm, body matt. Pronotum semi-elliptical, with evenly distributed, homogenous, distinct punctures on whole dorsal surface (Fig. 15). Elytra regularly rounded (more in larger specimens – compare Figs 9–11); with densely and evenly distributed medium-sized, distinct punctures of similar size (Fig. 18), rarely with two very fine, longitudinal lines that are occasionally more visible (Fig. 21). Rarely, individual larger punctures are intermixed, but never in such regular pattern as in *Ablattaria arenaria* and *Ablattaria cribrata*.

#### Remarks.

Both Fabricius (1775) and Sulzer (1776) refer in their descriptions of *Silpha laevigata* and *Silpha polita* to Geoffroy (1762: 122, species #8). However, the book of Geoffroy is not consistently binominal and Opinion 1754 (ICZN 1994) placed it on the Official List of Works in Zoological Nomenclature with only some generic names available. Accordingly, the author of *Silpha laevigata* is Fabricius and the author of *Silpha polita* is Sulzer.

In the syntype series of *Silpha laevigata* from ZMUC and BMNH, consistent with current understanding of *Ablattaria laevigata*, we also found intermixed a single specimen of *Silpha tyrolensis* Laicharting, 1781 (in ZMUC, “Kiel collection”; see above for details). This syntype specimen is here considered a paralectotype. We have designated a female from ZMUC, the “Copenhagen collection”, as the lectotype to fix this name as currently used.

*Ablattaria laevigata* is a widely distributed species with regional variation in size and shape between populations (see Geometric morphometrics section below), and also with some variability in punctation of elytra, sometimes with intermixed larger punctures or an impunctate pair of longitudinal lines present on elytra.

There are no distinct differences in the description of *Silpha polita* to separate it from *Ablattaria laevigata*, and we believe that this taxon is correctly considered as a junior subjective synonym of *Ablattaria laevigata* by Reitter (1884) and Schawaller (1979). In our opinion, the variation in body size, proportions and surface sculpturation which led to the description of *Silpha gibba* and several varieties of Ganglbauer (1899) and Portevin (1926) fall within the infrasubspecific variation of *Ablattaria laevigata*. We agree with Schawaller (1979), who considered Ablattaria gibba var. costulata, Ablattaria gibba var. distinguenda and Ablattaria gibba var. punctata as junior subjective synonyms of *Ablattaria laevigata*. Further, we consider Ablattaria laevigata var. meridionalis of Ganglbauer (1899) as a junior subjective synonym of *Ablattaria laevigata*.

#### Biology.

Seasonal activity of adults with a peak in April–June (Table 1).

#### Distribution.

**Figures 32–33.**
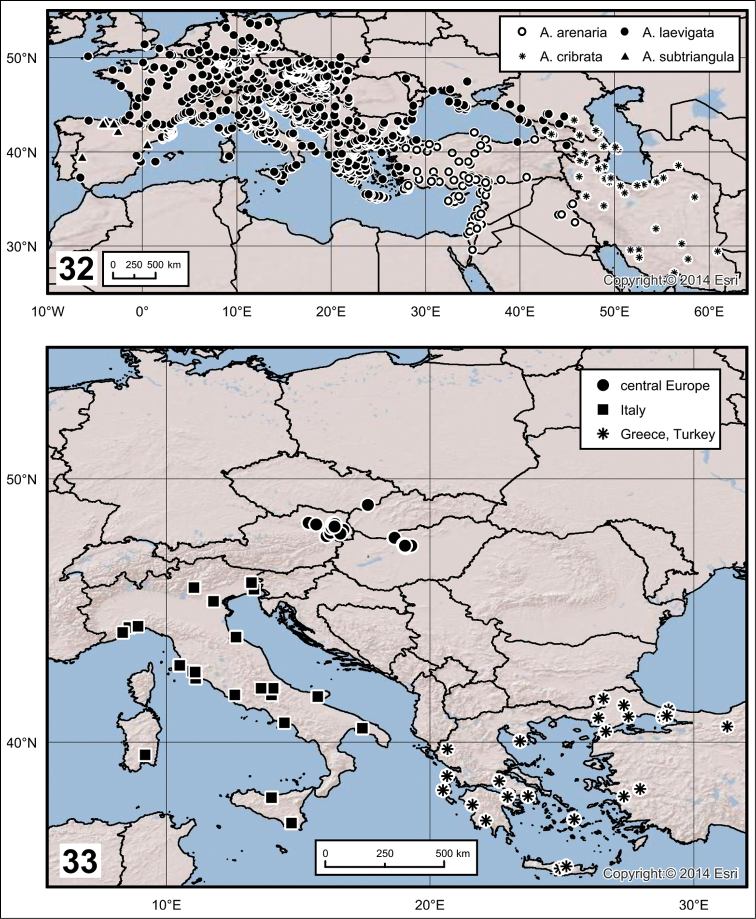
**32** Distribution of *Ablattaria* spp. in Western Palaearctic region **33** Delimitation and distribution of samples of *Ablattaria laevigata* used in geometric morphometrics.

Most of Europe; from the west (Spain to United Kingdom), through all of central and southern Europe, reaching to the east and north of Turkey; Ukraine, southern Russia, Georgia to Armenia (Fig. 32).

### Ablattaria subtriangula

Taxon classificationAnimaliaColeopteraSilphidae

Reitter, 1905

Figs 3 , 14 , 19 , 20 , 27

Ablattaria subtriangula Reitter 1905: 90 (type locality: “Spanien, Sierra de Guadeloupe [= Sierra de Villuercas mountain range], Cáceres [ca. 39°29'N, 06°22'W]).

#### Type material examined.

Lectotype male (here designated) (MNHN, coll. Pic), pinned, labelled: “SIERRA DE GUADALUPE / (CÁCERES) / MAI 1904 / G. SCHRAMM [p] // 71 [hw] // type [hw] // Ablattaria / laevigata / v. subtriangula Rtt. / (Reitt. vid.) [hw, probably Portevin’s handwriting] // Ablattaria subtriangula / Reitt. [hw, Pic’s handwriting] // Lectotype / Ablattaria subtriangula / Reitter, 1905 / J. Qubaiová & J. Růžička / des. 2014 [p, red label]. Paralectotype male (MNHN, coll. Marmottan) (Fig. 27), pinned, labelled: “SIERRA DE GUADALUPE / (CÁCERES) / MAI 1904 / G. SCHRAMM [p] // TYPE [p, red modern label] // Subtriangula / Reitt. / (Reynoza) [hw] // Muséum Paris / 1914 / Coll. H. Marmottan [p, modern label] // Paralectotype / Ablattaria subtriangula / Reitter, 1905 / J. Qubaiová & J. Růžička des. 2014 [p, red label]”.

#### Additional material examined.

18 specimens, see Appendix 1.

#### Diagnostic description.

Total body length 12–16 mm, body glossy, black (Figs 3, 14, 19). Pronotum almost conical in shape, margins anterolaterally constricted (Fig. 14); with evenly distributed, homogenous, distinct punctures on whole dorsal surface (Fig. 14). Elytra regularly rounded; with densely and evenly distributed medium-sized, distinct punctures of similar size (Fig. 19).

#### Remarks.

Additional male specimen (MNHN, coll. Marmottan), pinned, labelled: “Soto [hw] // TYPE [p, red modern label] // Silpha subtriangula / Reitt. / Co-type [hw, same handwriting as on identification label of lectotype specimen]” is not considered here as paralectotype, because its locality is not consistent with precise information provided in the original description by Reitter (1905). “Soto” is vague, as there seem to be more than 10 localities with this name across Spain (http://en.wikipedia.org/wiki/Soto), none of which are in either Cáceres Province or elsewhere in the Extramadura autonomous community.

#### Biology.

Regarding the seasonal activity of *Ablattaria subtriangula*, in the limited adult material examined, most specimens were collected between April and June.

#### Distribution.

Endemic to continental Spain (Fig. 32).

##### Geometric morphometrics

Relative warps (RWs) of both males and females of the four *Ablattaria* taxa were calculated and plotted on an axis system. The first RW (RW1) axis represented 44.25% of shape variability and the second axis (RW2) accounted for 20.22%. Subsequently, discriminant analysis (DA) was applied between the sexes on the first 30 axes representing 99.94% of variability. The results indicated shape sexual dimorphism (Hotelling’s test: 444.2, *F*: 14.071, *p* < 0.0001). Specimens correctly classified to their means showed a percentage of 82.59.

Male groups of the four taxa were tested independently and RW1 accounted for 44.74% of the total variance whereas RW2 accounted for 20.43%. A higher 46.98% of variability was explained by the RW1 axis in females and 18.73% by RW2. Both scatter plots of the two first RWs for male and female *Ablattaria* displayed a high overlap between the groups of the different taxa. The thin-plate spline (TPS) transformation grids (not included in the article) indicated some shape differences between the taxa especially in *Ablattaria arenaria*; less rounded or curved pronotal margins posteriorly and more parallel elytra. In *Ablattaria laevigata* the posterior pronotal margins appeared more rounded (semi-elliptical) and the elytra were more robust than the other taxa particularly in the females, whereas the pronotal shape of *Ablattaria subtriangula* was more narrowed to the front (conical).

Multivariate analysis of variance (MANOVA) was performed on the four groups. The results indicated significant shape variations, but the separations between the groups were weak, given that the number of *Ablattaria subtriangula* specimens was very low compared to those of other groups. Hence, the analysis was repeated without the *Ablattaria subtriangula* samples to obtain a clearer separation.

Shape diversity of both pronotum and elytra between the three taxa was indicated by MANOVA. Male groups revealed significant shape differences (*F* = 32.93; Wilk’s lambda = 0.0784; DF = 40/512; *p* < 0.00001). Female groups demonstrated higher body shape variability (*F* = 24.93; Wilk’s lambda = 0.1252; DF = 40/546; *p* < 0.00001).

**Figure 34.**
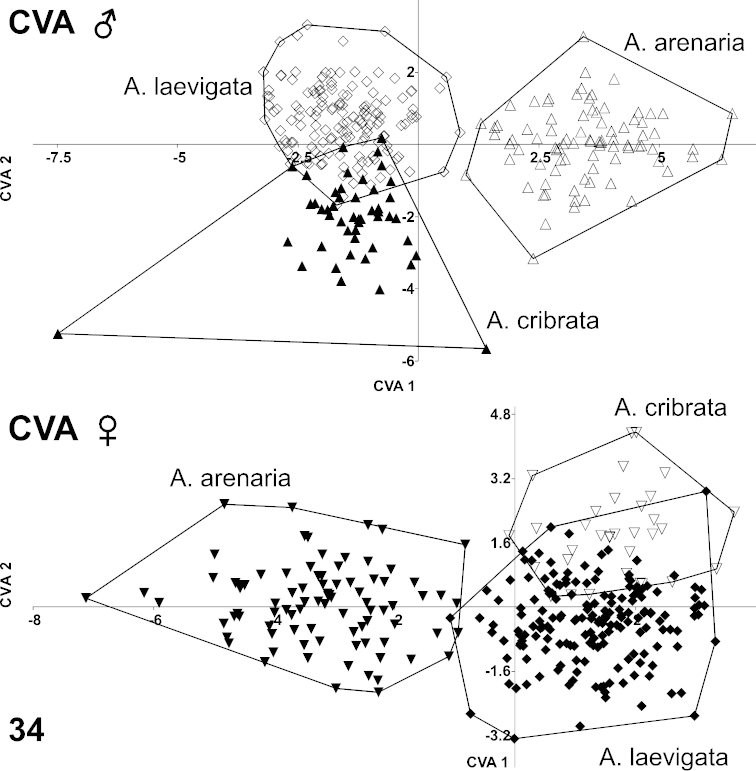
Canonical variate analysis (CVA) of male (above) and female (below) body shape changes in *Ablattaria*.

Two individual canonical variate analyses (CVA) for males and females (separately) were performed to obtain separation of the four groups on the first 20 axes of the RW scores matrix. These axes covered 99.81% of the shape variation between male groups and 99.82% between female groups. Results indicated no overlap between *Ablattaria arenaria* and either *Ablattaria laevigata* or *Ablattaria cribrata* in males and only with one specimen in females (Fig. 34). The overlap between *Ablattaria laevigata* and *Ablattaria cribrata* was minimal and more evident in males than in females.

The jackknifed (or leave-one-out) values of the confusion matrix in *Ablattaria laevigata* males illustrated a correct mean classification of 131 from 144 specimens (13 showed means closer to that of *Ablattaria cribrata*). In *Ablattaria arenaria*, this was the case for 84 of 85 (1 was closer in its mean value to that of *Ablattaria cribrata*). *Ablattaria cribrata* had 42 accurate classifications of 49 in total (7 specimens were closer to *Ablattaria laevigata*). In the females, 150 specimens of 175 in *Ablattaria laevigata* were correctly classified (24 were closer to *Ablattaria cribrata* and 1 to *Ablattaria arenaria*). In *Ablattaria arenaria*, 82 of 87 were correctly classified (4 were closer to *Ablattaria laevigata* and 1 to *Ablattaria cribrata*). In *Ablattaria cribrata*, 25 of 33 were clearly classified (8 were closer to *Ablattaria laevigata*). These findings strongly indicate the shape variations of these taxa, and thus support the hypothesis that all three taxa constitute separate species.

Nevertheless, both males and females (independently) of *Ablattaria subtriangula* were tested and compared with one group formed by the three other taxa to ensure its independence by discriminant analysis (DA). Results indicated significant shape variability in males (Hotelling’s test: 20.598, *F*: 5.0946, *p* < 0.001) with 86.93% correct classification of specimens to their means. For females (Hotelling’s test: 40.282, *F*: 10.465, *p* < 0.0001), specimens correctly classified were 85.48%. As a result, *Ablattaria subtriangula* indicates its division from the other taxa and therefore may also be considered as a separate species.

To examine allometry effects, the influence of size on body shape was tested first on the four taxa by separating them into groups based on taxon and sex. The multivariate regressions of shape onto size were performed one group at a time. Results showed significant relationship in both sexes of *Ablattaria laevigata*, males of *Ablattaria arenaria* and *Ablattaria subtriangula*, and females of *Ablattaria cribrata*. The results were insignificant for female *Ablattaria arenaria*, *Ablattaria subtriangula* and male *Ablattaria cribrata* (Table 2).

**Table 2.** Multivariate regressions of shape onto size for each *Ablattaria* species and sex separately.

Species	*Ablattaria laevigata*			*Ablattaria arenaria*		
	Explained variance	Goodall’s *F*-test		Explained variance	Goodall’s *F*-test	
Sex		*F*-value	*p*-value		*F*-value	*p*-value
Males	1.61%	2.33	*p* < 0.0001	1.70%	1.45	*p* < 0.01
Females	0.91%	1.59	*p* < 0.0001	1.60%	1.34	*p* < 0.1
**Species**	***Ablattaria cribrata***			***Ablattaria subtriangula***		
	**Explained variance**	**Goodall’s F-test**		**Explained variance**	**Goodall’s F-test**	
**Sex**		**F-value**	**p-value**		**F-value**	**p-value**
Males	1.30%	0.61	*p* = 0.9994	51.03%	3.13	*p* < 0.0001
Females	7.60%	2.55	*p* < 0.0001	4.79%	0.3	*p* = 1.00

Since allometry was significant in most taxa groups, size correction was provided by multivariate analysis of covariance (MANCOVA). This tool indicates if variation in shape is a result of size difference alone. MANCOVA was applied on male and female groups of the four taxa. Results suggested a significant interaction between body shape and body size (Table 3). Permutation tests with 1000 random permutations demonstrated a *p*-value of 0.00021 in males and 0.00087 in females. Considering that the percentage explained by size was 16.09% in males and 11.14% in females, some effect on the body shape variability between the taxa can be observed.

**Table 3.** Multivariate analysis of covariance (MANCOVA) for the four *Ablattaria* taxa.

	Explained variance	Goodall’s *F*-test	
Sex		*F*-value	*p*-value
Males	16.09%	6.57	*p* < 0.00001
Females	11.14%	4.61	*p* < 0.00001

Given that *Ablattaria laevigata* has such a wide ranging geographical distribution, it was interesting to examine the species’ body changes in various populations. Three different populations were studied: one from Greece and Turkey (Gr. & Tr.), a population from Italy (It.), and a population from Central Europe (CE) (geographic origins of examined specimens are summarized in Fig. 33). Relative warps were calculated in male and female populations separately and plotted on an axis system. The RW1 axis of males corresponded to 39.02% and RW2 to 18.99% of shape variability. In females, RW1 indicated 43.87% of shape variation and RW2 indicated 20.91%. TPS transformation grids (not included in the article) showed little shape variability; the elytra appeared, in general, more parallel in the Greek and Turkish populations. Populations from Italy had a more arched elytra and the pronotum was slightly broader (Figs 7 and 8).

**Figure 35.**
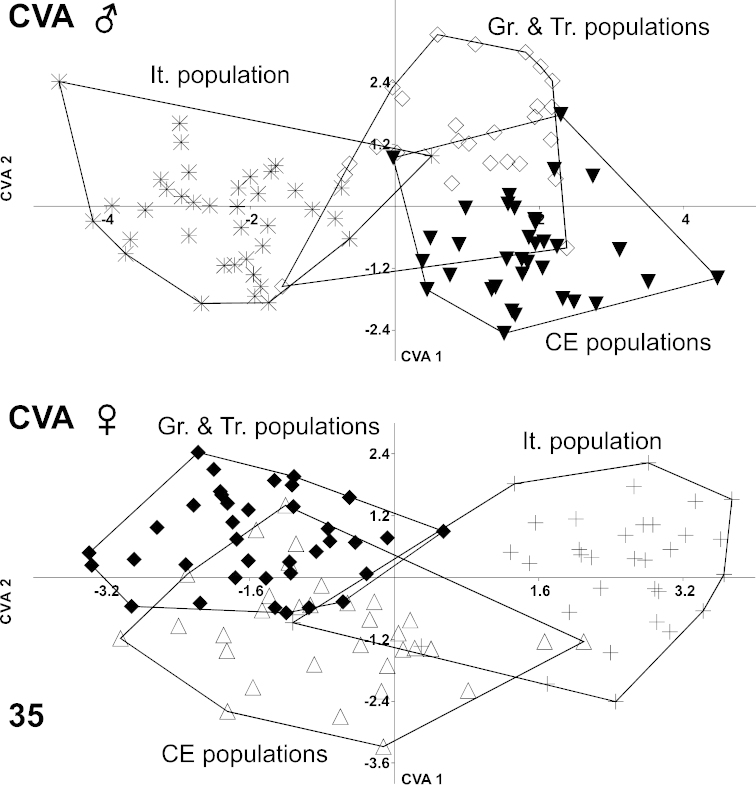
Canonical variate analysis (CVA) of male (above) and female (below) body shape changes in selected populations of *Ablattaria laevigata*; Gr. & Tr. (Greece & Turkey), It. (Italy), CE (Central Europe).

MANOVA was performed subsequently. Male populations revealed significant shape dissimilarity (*F* = 10.35; Wilk’s lambda = 0.121; DF = 30/166; *p* < 0.00001). Shape variability was found to be also significant in the female populations (*F* = 8.337; Wilk’s lambda = 0.166; DF = 30/172; *p* < 0.00001). Canonical variate analysis on the first 15 axes was performed and represented 99.54% of the shape variation in males and 99.68% in females. Results indicated overlap between all groups (Fig. 35). The jackknifed values of the confusion matrix for both sexes are presented in Tables 4 and 5. The most obvious separation was seen in the Italian population, which showed incorrect classification of only 7 specimens in the two sexes taken together of a total 72 specimens. The Central European population showed higher variation in the male than was that in the female populations from Greece and Turkey.

**Table 4.** Canonical variate analysis confusion matrix of male *Ablattaria laevigata* populations demonstrating the classification of specimens to the groups depending on their proximity to the various means.

*Ablattaria laevigata* populations, males	Pop. (Gr. & Tr.)	Pop. (It.)	Pop. (CE)	Total
Pop. (Gr. & Tr.)	**15**	4	7	26
Pop. (It.)	3	**35**	1	39
Pop. (CE)	7	0	**28**	35

**Table 5.** Canonical variate analysis confusion matrix of female *Ablattaria laevigata* populations.

*Ablattaria laevigata* populations, females	Pop. (Gr. & Tr.)	Pop. (It.)	Pop. (CE)	Total
Pop. (Gr. & Tr.)	**27**	1	9	37
Pop. (It.)	0	**30**	3	33
Pop. (CE)	10	2	**21**	33

In order to determine whether allometry played a role in this categorization, and even though the sample was too small, regression results indicated significant relationship between size and shape in both sexes (Table 6). Despite the fact that size explained a low percentage of the body shape (11.49% in males and 9.91% in females), its effect cannot be denied and the influence of allometry can be noted.

**Table 6.** Multivariate regression of log centroid size on shape for both sexes of the three populations.

	Explained variance	Goodall’s *F*-test		
Sex		*F*-value	DF	*p*-value
Males	11.49%	4.16	318/10176	*p* < 0.00001
Females	9.91%	3.63	318/10494	*p* < 0.00001

**Figure 36.**
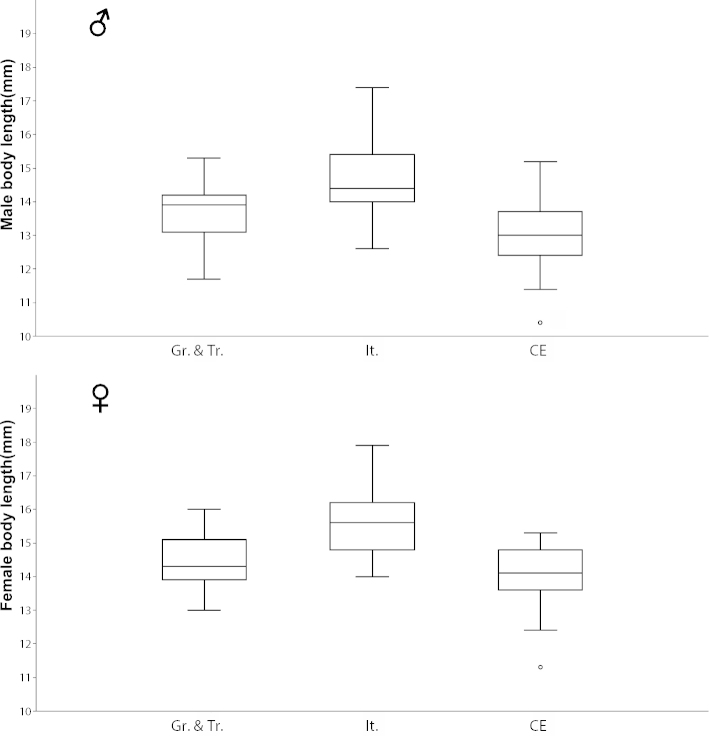
Boxplots of male (above) and female (below) body length in selected populations of *Ablattaria laevigata*; Gr. & Tr. (Greece & Turkey), It. (Italy), CE (Central Europe).

The linear size (body length of pronotum and elytra) of both sexes was measured and plotted in a simple boxplot (Fig. 36). In general, females were larger than males. Even though body length differences did not appear to be very marked, the smallest of the three groups was the population from Central Europe, particularly the males, whereas the females were only slightly smaller than the Greek and Turkish females. The largest specimens measured here were those from Italy.

## Discussion

Geometric morphometric techniques were applied to this genus for the first time. Based on the results we obtained and the observed morphological traits, *Ablattaria cribrata* can be considered a separate species. This agrees with Schawaller (1979) but not with Nikolaev and Kozminykh (2002), who treated *Ablattaria cribrata* as a subspecies of *Ablattaria laevigata*.

*Ablattaria laevigata* has a vast distribution over the Western Palearctic region. Hence, both its shape and size vary greatly across its range. Applying the geometric morphometric techniques to various populations indeed confirmed these shape variations (see above). These results, together with further morphological examination of other populations, revealed that beetles from the Mediterranean region (specimens from Croatia, Italy, Greece and Turkey) tend to be larger in body size than those from Central Europe.

The geographical separation of the identified species can be observed most particularly in *Ablattaria subtriangula*, which is endemic to Spain and is syntopic only with *Ablattaria laevigata*. *Ablattaria subtriangula* is absent from the Balearic Islands, where only *Ablattaria laevigata* is found. This is in agreement with Piloña et al. (2002). *Ablattaria cribrata* is widespread throughout Iran (in contrast to data in Portevin (1926) and Schawaller (1979)) and is separated from *Ablattaria arenaria* at the eastern Iraqi – western Iranian borders, where only one specimen of *Ablattaria arenaria* has been noted (although two specimens of *Ablattaria arenaria* were recorded from Iran: Huzestan prov. by Růžička and Schneider (2002)). *Ablattaria laevigata* overlaps in its occurrence with *Ablattaria arenaria* mainly in eastern and more sparsely in northern Turkey. *Ablattaria arenaria* does not appear to cross into continental Europe. *Ablattaria laevigata* and *Ablattaria cribrata* partially overlap in Georgia, Armenia and southern Russia (specifically in Chechnya). The genus does not seem to extend beyond southern Russia and Iran to Kazakhstan or Afghanistan. It is rather scarce in Turkmenistan, where it is known only in the south-western part (as also mentioned by Nikolaev and Kozminykh (2002)). Moreover, there are no known records of *Ablattaria* from northern Africa or from the Arabian Peninsula.

Schawaller (1979) stated that the genus only rarely occurs at higher elevations. We examined about 52 specimens from localities above 2000 m (140 specimens from localities above 1000 m). Some of the records of *Ablattaria laevigata* from high elevations were from Italy: Molise Reg., Majella Mt. at 2793 m. Also, *Ablattaria arenaria* was cited from Syria: Nur Mts. (Amanus) at 2240 m, and Israel: Mount Hermon at 2000 m. Schawaller (1979) also speculated whether the genus could be found in the higher Pyrenees and Alps. Two specimens of *Ablattaria laevigata* were recorded from the French side of the Pyrenees (Languedoc-Roussillon Reg.: Lac d’Estom and Arles sur Tech), and two specimens from the Spanish side (Catalonia: La Jonquera and Espot). Six specimens of *Ablattaria laevigata* were recorded from localities higher than 1000 m in the Alps, the highest being from Provence-Alpes-Côte d’Azur Reg.: Vaucluse Dept., Mont Ventoux, at an altitude of ca. 1900 m.

## Supplementary Material

XML Treatment for Ablattaria

XML Treatment for Ablattaria arenaria

XML Treatment for Ablattaria cribrata

XML Treatment for Ablattaria laevigata

XML Treatment for Ablattaria subtriangula

## Appendix 1

List of non-type material of *Ablattaria* spp.

### Ablattaria arenaria

**Cyprus:** Akrotiri forest, 16.iii.1936, G. A. Mavromoustakis leg., 1 ♂ (BMNH); **Dhekelia** (British Overseas Territory): at light, 7.x.1957, M. Connelly, 1 ♀ (BMNH); same locality, 21.iv.1958, M. Connelly leg., 1 ♀ (BMNH); same locality, iii.1986, G. Sama leg., 1 ♂ (SMNS); **Kyrenia Dist.:** Asomatos, 21.x.1943, G. A. Mavromoustakis leg., 1 ♂, 1 ♀ (BMNH); Karavas, 1.-4.v.[19]69, J. Kohousek leg., 1 ♂, 1 ♀ (JRUC); same locality, without date, A. Pfeffer leg., 1 ♂ (NMPC); Kyrenia (Mts.), without date, E. Hanzlikova leg., 2 ♂ (NMPC); **Larnaca Dist.:** Larnaca, 6.iv.[19]81, Cl. Besuchet leg., 1 ♂, 1 ♀ (MHNG); same locality, 1896, Hesse leg., 1 ♂, 2 ♀ (SMNS); **Limassol Dist.:** Limassol, 15.v.1918, G. A. Mavromoustakis leg., 1 ♂ (BMNH); same locality, 24.x.1929, G. A. Mavromoustakis leg., 1 ♂ (BMNH); same locality, 1930, Mavromoustakis leg., general coll., 1 ♀ (SDEI); same locality, ii.1933, G. A. Mavromoustakis leg., 2 ♂ (BMNH); same locality, 24.iv.-15.v.1977, R. Köstlin leg., 1 ♂ (SMNS); same locality, coll. W. H. Muche, 1 ♂ (SMTD); Yermasoyia Hills, 15.ii.1952, G. A. Mavromoustakis leg., 1 ♀ (BMNH); Yermasoyia river, 10.ii.1947, G. A. Mavromoustakis leg., 1 ♀ (BMNH); **Paphos Dist.:** Kanaviou, 14.iv.1992, D. & T. Osten leg., 1 ♀ (SMNS); Paphos, 21.v.1988, Konigagraber & T. Osten leg., 1 ♀ (SMNS); same locality, Limentiotissa ruins, 20.ii.2002, M. V. L. Barclay leg., 1 ♀ (BMNH); same locality, Tombs of the Kings, 23.ii.2002, M. V. L. Barclay leg., 1 ♂ (BMNH); **Cyprus without specific locality:** 5.xii.1988, Grimm & Rachinsky leg., Coral bay, 1 ♀ (SMNS); iv.1986, Bilek & Kritscher leg., 1 ♀ (NHMW); without date and collector, coll. Reitter, 1 ♀ (HNHM);

**Greece:** **Central Macedonia Reg.**, Sithonia, 20.vi.-4.vii.1992, P. Průdek leg., 1 ♀ (JRUC); **Crete Island:** without date and collector’s name, coll. E. Frivaldszky, 1 ♂, 1 ♀ (HNHM); Miramare (Resort area), 25.iv.1962, W. H. Tams leg., 1 ♂ (BMNH); same locality, 15.iv.1962, W. H. Tams leg., 1 ♂ (BMNH); same locality, 22.iv.1962, W. H. Tams leg., 1 ♂ (BMNH); **South Aegean Reg., Rhodes Island:** Apolakia, 26.iv.1990, D. Lange & T. Osten leg., 1 ♂ (SMNS); Arhangelos, 8.iv.1980, Schawaller leg., 1 ♂, 1 spec. (SMNS); Epano Kalamon, 4.iv.1996, R. Grimm leg., 1 ♂ (SMNS); Filerimos (village hotel), 6.iv.1996, R. Grimm leg., 2 ♂ (SMNS); Kattavia, 10 m, 9.v.1985, Wienser and Worn leg., 2 ♂ (SMNS); Koskinou, 20 m, 6.v.1985, Wiesner & Worm leg., 1 ♂, 1 ♀ (SMNS); Lardos, Laerma, 28.vi.2007, K. Orszulik leg., 1 ♂ (KORC); Lindos, 27.iv.1964, Helversen leg., 2 ♂ (SMNS); same locality, 4.iv.1980, Schawaller leg., 1 ♂, 2 ♀ (SMNS); same locality, 16.-30.iv.1981, Köstlin leg., coll. R. Köstlin, SMNS 1987, 1 ♀ (SMNS); Rhodes (city): 50 m, 17.v.1985, Wiesner & Worm leg., 1 ♂, 1 ♀ (SMNS); same locality, without date, Oertzen leg., 4 ♂, 1 ♀(ZMHB); **Rhodes Island (without specific locality):** without date, B. Schwarzer leg., 1 ♂ (SMFD); without date, Reitter leg., coll. V. Zoufal, 2 ♂, 2 ♀ (MZMB); without date, Lichtenckert leg., 1 ♂ (HNHM); without date and collector’s name, coll. W. H. Muche, 21 ♂, 13 ♀ (SMTD); without date and collector’s name, coll. Dr. E. Jagemann, 2 ♂, 2 ♀(MZMB); without date and collector’s name, coll. Prof. Noesske, 3 ♂, 7 ♀(SMTD); without date and collector’s name, coll. Reitter, 1 ♂ (HNHM); without date and collector’s name, 2 ♂, 1 spec. (HNHM); without date and collector’s name, 1 ♂, 1 ♀ (ZMHB); without date and collector’s name, 1 ♂, 2 ♀ (NMPC); Katta [not located], 12.iv.1977, Cl. Besuchet leg., 1 ♂ (MHNG); 6.iv.1996, R. Grimm leg., 1 ♂ (SMNS);

**Iran:** Kermanshah Prov., Qasr-e-Shirin, 2.v.1914, Nesterov leg., 1 ♀ (ZMAS);

**Iraq:** Abu-Ghraib, iii.[19]84, I. Smatana leg., 1 ♂, 1 ♀, 1 spec. (JRUC); Al-Kut, iv.1914, A.R. D’Abreu leg., coll. Andrewes Bequest, 1 ♂ (BMNH); Baghdad, 8.iii.1920, Y. R. Roscoll leg., 1 ♂ (BMNH); same locality, summer 1928, leg. R. W. G. Hingston, 1 ♂ (BMNH); same locality, 8.iii.1946, without collector’s name, 1 ♀ (BMNH); same locality, without date, Kalalova leg., 1 ♂ (NMPC); same locality, without date and collector’s name, coll. Sharp 1905, 1 ♀ (BMNH); Khanaqin, 26.-29.iv.1914, Nesterov leg., 1 ♀ (ZMAS);

**Israel:** **Central Dist.**, Ramla, 9.iv.1992, H. Mienis leg., 1 ♂ (SMNS); **Coastal plain Reg.**, Nahsholim (20 km South Haifa Nat. Res., Hof Dor-Ha Bonim) [32.37° N 34.54° E], 28.ii.1996, U. Göllner leg., 1 ♂ (ZMHB); **Golan Heights:** Al-Mahjar, 200 m, 27.iv.1982, Besuchet & Löbl leg., 1 ♀ (MHNG); Mount Hermon, 2000 m, 3.viii.1995, V. Chikatunov leg., 1 ♂ (TAUM); Nimrod Fortress (Qalaat Nimrod), 300-600 m, 7.iv.1985, Heinz leg., 1 ♀ (SMNS); **Haifa Dist.:** Beit Oren, 24.ii.1998, V. Chikatunov & T. Pavlíček leg., 1 ♀ (TAUM); Haifa, 1884, coll. Reitter, 1 ♂, 2 ♀ (HNHM); same locality, 1884, coll. Reitter, 1 ♂, (SMTD); same locality, iv.1927, without collector’s name, 1 ♀ (SMTD), same locality, without date, B. Schwarzer leg., 1 ♂ (SMFD); same locality, without date, Schonfeldt leg., 2 ♀ (SMFD); same locality, without date and collector’s name, coll. Reitter, 1 ♂ (HNHM); Mount Carmel (South-East), Eliakim (South-West), 300 m, 11.ii.1987, Schawaller & Schmalfuss leg., 1 ♂ (SMNS); **Jerusalem Dist.:** Jerusalem, 12.ii.1956, J. Klapperich leg., 1 ♂ (HNHM); same locality, Simon leg., 1 ♂ (ZSM); same locality, without date and collector’s name, 2 ♀ (NMPC); same locality, without date and collector’s name, coll. W. H. Muche, 1 ♀ (SMTD); Tzora, 5.iv.1996, V. Chikatunov leg., coll. V. Chikatunov, 1 ♀ (TAUM); **Northern Dist.:** Ahihud, 9.-20.iv. (without a year), Heinz leg., 1 ♀ (SMNS); Nahariya, 2.-8.iv.1978, T. Osten leg., 1 ♂ (SMNS); Nazareth, 10.iii.1910, P. J. Barraud leg., 1 spec. (BMNH); same locality, 20.iii.1987, C. Blumenthal leg., 1 ♀ (SMNS); same locality, 15.iii.-3.iv.1987, C. Blumenthal leg., 2 ♀ (SMNS); upper Galilee (open stony grazing land, dolomite), South Zivon [33°01.189'N, 35°24.914'E], 750 m, 8.iii.2008, D. W. Wrase leg., 3 ♂, 2 ♀ (SMNS); upper Galilee, Hula Valley, Ma’agar Einan reservoir (toe of dam in moist loamy soil) [33°05.137'N, 35°34. 730'E], 73 m, 1.-2.v.2006, D. W. Wrase leg., 1 ♀ (JCOC); same locality, 73 m, 1.-3.v.2006, D. W. Wrase log., 1 ♀ (JCOC); same locality, 73 m, 1.-4.v.2006, D. W. Wrase log., 1 ♀ (JCOC); **North-western Negev Reg.**, Ein HaBesor, 30.iii.1987, Heinz leg., 1 ♀ (SMNS); **Shephelah Reg.**, Netzer Sereni, 17.iv.1992, H. Mienis leg., 1 ♂ (SMNS); **Southern Dist.**, Bitronot Ruhama, Nachal Zedim [31°32'45"N, 34°41'43"E], 5.iv.2005, L. Friedman leg., 1 ♀ (TAUM); **Tel Aviv Dist.:** Herzliya, hill [32°11'N, 34°49'E], 30.i.2009, A. Freidberg leg., 1 ♀ (TAUM); Herzliya, sandy area, Schweiger leg., 1 ♂, 1 ♀ (SMFD); Tel Aviv, 4.xii.1969, Prys leg., 1 spec. (TAUM); **The West Bank**, Jericho, 23.ii.1941, Bytinski-Solz leg., 1 ♂ (TAUM); Without specific locality, 20.iii.1934, F. S. Bodenheimer leg., 1 ♀ (TAUM);

**Jordan:** Jarash (50 km North Amman), 13.iii.1977, Scheuern leg., 1 ♀ (SMNS); same locality, 30.iv.2004, Lillig & Pavlicek leg., 1 ♂, 1 ♀ (SMNS); Wadi Araba, 1.iii.1983, Ikabo Yamen leg., 1 ♂ (BMNH); Zerkatal near Romana, 3.iii.1957, J. Klapperich leg., 1 ♀ (HNHM);

**Lebanon:** Beirut, without date and collector’s name, coll. L.W. Schaufuss, 1 ♂ (ZMHB); same locality, without date, Plason leg., 3 ♂ (ZMHB); same locality, without date, Plason leg., coll. Pinker, 1 ♂, 1 ♀ (NHMW); Majdal Zun, 7.v.1981, F. Kervink leg., 1 ♂ (ZMAN); Lebanon without specific locality, date and collector’s name, 1 ♂, 1 ♀ (ZMHB);

**Syria:** **Aleppo Governorate:** Maydan Ikbis (Al Midan Akbes), without date, A. Buchta leg., 1 ♀ (ZMHB); same locality, without date, ex coll. Em. Reitter, coll. V. Zoufal, 1 ♂ (MZMB); **Hama Governorate:** Afamia (Apamea) ruins (North-West Hama), 25.iii.1980, Klapperich leg., 1 ♂ (SMNS); Apamea, Qalaat al-Mudiq (North-West Hama), 23.iii.1979, Kinzelbach leg., 1 ♀ (SMNS); **Latakia Governorate:** Latakia, ruins of Ugarit, 4.iii.1979, Kinzelbach leg., 1 ♂, 1 ♀ (SMNS); same locality, 200 m, 9.iv.1978, Heinz leg., coll. Haffe, 1 ♀ (SMNS); Nur (Amanus) Mts., v.1902, Escalera leg., 1 ♂ (MNCN); **Syria without specific locality:** without date, Rosenbach leg., 1 ♂ (SMFD); without date and collector’s name, coll. Sharp, 1905-313, 1 ♂ (BMNH); without date and collector’s name, 2 ♀ (BMNH); without date and collector’s name, 2 ♂ (MHNG);

**Turkey:** **Aegean Reg.:** Bozdağ, without date, Bodemeyer leg., 1 ♂, 2 ♀ (ZMHB); Çameli (6 km South-West), 22.iv.1984, Krätschmer leg., 1 ♂, 2 ♀ (SMNS); Denizli prov., 2.vi.1967, E. Baudisch leg., 1 ♂ (SMNS); same locality, Pamukkale, 28.iv.1992, Kapler leg., 1 ♀ (JRUC); same locality, Hierapolis, 21.iv.1973, Bremer leg., 1 ♂ (SMNS); same locality, 25.-27.vi.2004, V. Hula leg., 4 ♂, 5 ♀ (JRUC); **Anatolia Reg.:** without date, Bodemeyer & Weistritz leg., 2 ♂, 1 ♀ (NMPC); same locality, without date, Obenberger leg., 1 ♂ (JRUC), same locality, without date, Obenberger leg., 1 ♂ (NMPC); Cilician Gates or Gülek Pass, v. (without year), Muche leg., coll. W. H. Muche, 1 ♂, 4 ♀ (SMTD); Yen Chehir, 1900, J. Fodor leg., coll. J. Fodor, 1 ♀ (HNHM); **Black Sea Reg.:** Amasya, 20.vi.1966, Ressl leg., 1 ♂ (SMNS); same locality, 1888, B. Schwarzer leg., 1 ♂ (SMFD); same locality, without date, J. Fodor leg., 1 ♂ (HNHM); same locality, without date, K. Neumann leg., 1 ♀ (SMFD); same locality, without date, Korb leg., 1 ♂ (NMPC); same locality, without date, O. Leonhard leg., general coll., 1 ♂, 1 ♀ (SDEI); same locality, without date and collector, coll. W. H. Muche, 1 ♂, 1 ♀ (SMTD); same locality, without date and collector, coll. J. Fodor, 1 ♀ (HNHM); same locality, without date and collector, 1 ♀ (HNHM); Merzifon, Samsun, 27.vi.1971, Bettou leg., 1 ♂ (MHNG); Mudurnu, 20.v.1996, I. Smatana leg., 2 ♂ (JRUC); Murgul-yayla, forest zone, 6.-7.vii.1983, Heinz leg., 1 ♀ (SMNS); Sinop, 1888, Retowski leg., 1 ♀ (SMFD); Tokat, 700 m, 17.iv.1986, A. Korell leg., 1 ♀ (SMNS); **Central Anatolia Reg.:** Ankara, vii.1926, R. Vogel leg., 1 ♀ (SMNS); same locality, 12.vii.1939, F. S. Bodenheimer leg., 1 ♀ (BMNH); same locality, 3.iv.1963, Ressl leg., 1 ♂ (SMNS); same locality, 900 m, in a park, singled from ground, 27.vi.1980, O. Merkl leg., 1 ♀ (HNHM); Ereğli, 1000-1200 m, 1.iv.1978, Heinz leg., 1 ♀ (SMNS); Hacıbektaş, Gümüşkent, 21.iv.1992, M. Kocian leg., 1 ♂ (JRUC); Konya, 1899, O. Leonhard leg., general coll., 1 ♀ (SDEI); same locality, without date, Bodemeyer leg., coll. Prof. Noesske, 2 ♀ (SMTD); Nevşehir-Göre, 21.iv.1992, M. Kocian leg., 1 ♂ (JRUC); Şereflikoçhisar (15 km South), 24.iv.1973, Heinz leg., 1 ♂ (SMNS); Taurus (Mts.): 1895, Holtz leg., 1 ♀, 1 spec. (ZMHB); without date, A. Kricheldorff leg., coll. Prof. Noesske, 1 ♀ (SMTD); without date, Klein leg., 1 ♂ (JRUC); without date, Klein leg., coll. V. Zoufal, 1 ♂ (MZMB); without date and collector’s name, 5 ♂, 2 ♀ (NMPC); Bulghar Maaden [Bolkar Mountains], Bodemeyer leg., 1 ♂ (ZMUC); Pozantı, 1.v.1969, Klapperich leg., 1 ♀ (SMNS); Yozgat prov., Sorgun (5 km East), vii.1974, Pretzmann & Kasy leg., 1 ♀ (NHMW); **Marmara Reg.:** Adapazarı, iv.-v. (without year), Muche leg., coll. W. H. Muche, 1 ♀ (SMTD); Bandırma, 1973, Heinz leg., 1 ♂ (SMNS); Bursa, 1870, Pavel leg., 2 ♀ (HNHM); same locality (25 km East), 600 m, 9.iv.1986, Krätschmer leg., 1 ♂ (SMNS); Gölpazari, Beşevler, 450 m, 28.-30.v.1996, I. Smatana leg., 1 ♂ (JRUC); **Mediterranean Reg.:** Adana prov.: Tumlu, Kalesi castle, rocky stepe on limestone [37°09'00"N, 35°42'25"E], 44 m, P. Kment leg., 1 ♂, 1 ♀ (NMPC); Yılankale, rocks opposite to Yilankale castle [37°0'52"N, 35°44'52"E], 78 m, shrubs+herbs, P. Kment leg., 1 ♀ (NMPC); Adana, 1905, without collector’s name, 1 ♂ (JRUC); same locality, 1937, without collector’s name, ex coll. G. Paganetti Hummler, coll. P.v.d. Wiel, 1 ♂, 1 ♀ (ZMAN); same locality, without date, H. Holle leg., 1 ♀ (ZMHB); same locality, without date, Kouřil leg., 1 ♂, 2 ♀ (NMPC); same locality, without date, Štěrba leg., 5 ♂, 2 ♀ (NMPC); same locality, without date and collector’s name, 2 ♀ (NMPC); same locality, Çukurova University, 10.x.2006, N. Jansson leg., 1 ♂, 1 ♀ (NJAC); same locality, Yumurtalık, 25.v.1993, P. Průdek leg., 1 ♂ (JRUC); Adana-Bakali, 19.vi.1980, F. Ozqur leg., 1 ♂, 1 ♀ (SMNS); same locality, 31.v.1986, F. Ozqur leg., 1 ♀ (SMNS); Alma Dağ (Mt.), without date, Bodemeyer leg., coll. Prof. Noesske, 1 ♂, 2 ♀ (SMTD); Altinözü, 22.iv.1992, Kapler leg., 1 ♂ (JRUC); Antalya, Konyaalti city park, 10.iv.2012, Jan Vitner leg., 3 ♂, 1 ♀ (JRUC); Belen Pass (Belen Geçidi), 9.iv.1992, Z. Švec leg., 1 ♀ (JRUC); Çamlıyayla, 1300 m, 1.vii.1998, G. Sama leg., 1 ♂ (SMNS); Ceyhan, vi.1937, Vasvari leg., 1 ♀ (HNHM); same locality, without date, Vasvari leg., 1 ♀ (HNHM); Gelemen (Noeth Samsun), 4.v.1959, K. M. Guichard leg., 1 ♂ (BMNH); İskenderun (Alexandrette), without date, A. Kricheldorff leg., coll. R. Formánek, 1 ♀ (MZMB); Isparta prov., Eğirdir-çadır, 7.v.1975, Besuchet & Löbl leg., 1 ♀ (MHNG); Issus, 100 m, 18.iv.1973, Heinz leg., 1 ♀ (SMNS); Kargıcak, 200 m, 11.iv.1981, Heinz leg., 1 ♀ (SMNS); Mersin, 1906, Ariamia leg., 1 ♂ (ZMHB); same locality, 1906, without collector’s name, 1 ♀ (ZMHB); same locality, without date, A. Kricheldorff leg., coll. J. Fodor, 1 ♀ (HNHM); Namrun, v.1967, I. Dr. Schurmann leg., 1 ♂, 1 ♀ (SMNS), same locality, without date, Petrovitz-Ressl leg., 1 ♂ (NHMW); Silifke, on plant, 5.iv.1972, Bremer leg., 1 ♂ (SMNS); Sülek, without date, Schutz leg., 1 ♂, 1 ♀ (NMPC); same locality, without date and collector’s name, 1 ♂ (NMPC); Yakacık (North Iskenderun), 23.iv.1984, Krätschmer leg., 1 ♀ (SMNS); Yayladağı, 600-900 m, 13.iv.1981, Heinz leg., 1 ♂ (SMNS); Yumurtalık, v.1986, H. Donner leg., 1 ♀ (ZMAN); **Southeastern Anatolia Reg.:** Bireçik, without date, Bodemeyer leg., 1 ♂ (ZMHB); Mardin, without date and collector’s name, coll. W. H. Muche, 1 ♂ (SMTD); **Turkey without specific locality:** without date and collector’s name, coll. Piesbergen, 1 ♀ (SMNS); without date, E. Witt leg., 1 ♀ (SMFD).

### Ablattaria cribrata

**Armenia:** Armen Mt. [not located], without date, A. Weis leg., coll. A. Weis, 1 ♂ (SMNS); Khustup, 27.vi.1982, M. Danilevsky leg., 1 ♂ (ZMUM);

**Azerbaijan:** **Absheron, Baku Dist.:** Absheron National Park, without date, Kryzhanovskiy leg., 2 ♂, 1 ♀ (ZMAS), Baku city, 14.v.2007, K. Orszulik leg., 2 ♀ (KORC); same locality, without date, Schonfeldt leg., 1 ♀ (SMFD); Zağulba Bağları, 24.v.1970, Kryzhanovskiy leg., 2 ♂ (ZMAS); same locality, 30.v.1970, Kryzhanovskiy leg., 1 ♂ (ZMAS); same locality, 31.v.1970, Kryzhanovskiy leg., 1 ♀ (ZMAS); **Lankaran Reg.**, Astara, 28.v.2007, K. Orszulik leg., 1 ♀ (KORC); **Shirvan Reg.**, Sündü, Şamaxı, vi.2007, K. Orszulik leg., 1 ♂ (KORC); **Nagorno-Karabakh Rep.**, Stepanakert, 28.viii.1984, Tupavkin leg., 1 ♀ (ZMUM);

**Georgia:** Sioni (Livnetskiy Reg.), 14.vi.1974, Dzambazishich leg., 1 ♂ (ZMUM); Sioni (Planetsk. Reg.), 10.vi.1975, Mlokos’vic leg., 1 ♂ (ZMAS); **Kakheti Reg.**, Lagodekhi, 1896, Mlokos’vic leg., 1 ♂ (ZMAS); **Samtskhe-Javakheti Reg.:** Borjomi Dist., Borjomi-Kharagauli, dry slope, 2 km East Quabiskhevi, 29.vii.2006, D. Bartsch leg., 1 ♀ (SMNS); same locality, 17.vii.1896, without collector’s name, coll. G. Sivers, 1 ♂ (ZMAS);

**Iran:** Arasbaran wildlife refuge, between Makidi and Mahmudabad, 2000-2300 m, 10.vi.[19]78, Martens and Pieper leg., 1 ♂ (SMNS); **Ardabil prov.**, Ardabil, ix.1999, H. Ghahari leg., 1 ♂ (JRUC); **East Azerbaijan prov.:** Arasbaran, ix.2001, without collector’s name, 1 ♀ (JRUC); same locality, viii.2003; without collector’s name, 1 ♀ (JRUC); Maragheh, xii.2004, N. Samin leg., 1 ♂ (JRUC); **Fars prov.:** Kazerun, vii.1998, H. Ostovan leg., 1 ♂ (JRUC); Piruz Apad, vi.2003, S. Hesami leg., 1 ♂ (JRUC); without specific locality, vi.2000, Kazeron leg., 1 ♂ (JRUC); **Ghazvin prov.**, Alamut to Qazvin, viii.2002, H. Ghahari leg., 1 ♀ (JRUC); **Gilan prov.:** Masule (55 km South-West Rasht), 15.-17.iv.1999, J. Rejsek leg., 3 ♂ (SMNS); Rasht, vi.-vii.1980, Saver leg., 1 ♂ (SMFD); Rudbar, 1.vi.2001, Orszulik leg., 1 ♂, 1 ♀ (JRUC); **Golestan prov.:** Gorgan (Astrabad), iv.-vi.1908, O. Leonhard leg., general coll., 2 ♂, 1 ♀ (SDEI); same locality, iv.-vi.1908, O. Leonhard leg., coll. Prof. Noesske coll. 1947, 1 ♂ (SMTD); same locality, 15.iv.1958, Vinert leg., 1 ♂ (ZMAS); same locality, 15.iii.1904, Filinnovich leg., 1 ♂ (ZMAS); same locality, 16.vi.1905; Filinnovich leg., 1 ♀ (ZMAS); same locality, without date and collector’s name, coll. Prof. Noesske, 1 ♀ (SMTD); same locality, without date and collector’s name, 1 ♀ (ZMHB); Gonbad-e Qabus, ix.2003, Z. Karimian leg., 1 ♂ (JRUC); Golestan forest, vi.1998, H. Sakenin leg., 1 ♂ (JRUC); **Hamadan prov.**, Malayer, viii.2004, H. Ghahari leg., 1 ♂ (JRUC); **Hormozgan prov.**, xii.2005, Bandar-Abbas leg., 1 ♀ (JRUC); **Kerman prov.:** Jiroft, vii.2005, H. Ghahari leg., 1 ♀ (JRUC); Kerman, vi.2004, H. Ostovan leg., 1 ♂ (JRUC); **Kurdistan prov.**, vii.2001, Sanandaj leg., 1 ♂ (JRUC); **Māzandarān prov.:** Amol (35 km South) [36°22'01"N, 52°20'25"E], 500 m, 3.v.1998, Gy. Fabian & K. Szekely leg., 1 ♀ (HNHM); Māzandarān prov. or Gilan prov., 25.v.2001, Orszulik leg., 1 ♀ (JRUC); **Razavi Khorasan prov.**, Kashmar, viii.2003, H. Ghahari leg., 1 ♀ (JRUC); **Sistan and Baluchestan prov.**, vii.2004, H. Rakhshanii leg., 1 ♀ (JRUC); **Tehran prov.**, Shahreyar, ix.2003, H. Ghahari leg., 1 ♂ (JRUC); **Not identified:** Shachnisan shachrub [not located], 30.v.1942, E. Lavlovskiy leg., 1 ♂ (ZMAS);

**Russia:** **North Caucasian Federal Dist.**, Chechnya, Gudermes, vi.1954, Ivanov leg., 2 ♂ (ZMAS); Dagestan, without date, A. Weis leg., 1 ♀ (SMFD); same locality, without date, B. Schwarzer leg., 1 ♂ (SMFD); same locality, without date, coll. Reitter, 1 ♂ (HNHM); Derbent, without date, Desbroch leg., 1 ♂ (ZSM); same locality, without date, E. Witt leg., 1 ♀ (SMFD); same locality, without date, Schonfeldt leg., 1 ♂ (SMFD); same locality, without date and collector’s name, coll. Letzner, general coll., 1 ♂ (SDEI); same locality, without date and collector’s name, 1 ♀ (ZMAS); same locality, without date and collector, 2 ♂, 1 ♀ (HNHM); Novyye Vikri, 25.v.1985, Abgurakhm leg., 1 ♂, 1 ♀ (ZMAS);

**Turkmenistan:** Kopet Dag (Mts.), Kara-Kala, 29.iv.1970, L. Zimina leg., 1 ♂ (ZMUM);

**Not located:** Eldar, El’arougikaz. u. (village), El. g., 21.iv.1910, Mlkosev leg., 1 ♀ (ZMAS).

### Ablattaria laevigata

**Albania:** Vermosh, 1914, Penther leg., 1 ♂, 1 ♀ (NHMW); without detailed locality, without date and collector’s name, coll. Prof. F. Werner, 1 ♂, 1 ♀ (NHMW); without detailed locality, without date and collector’s name, 1 ♀ (SMFD);

**Armenia:** Sevan, Covagyukh [Gegharkunik prov.], 1988, Strejček leg., mountain steppe, under stone, 1 ♂ (JRUC); Semyonovka (Diliyan), 4.vi.1989, S. Bečvář leg., 1 ♀ (JRUC);

**Austria:** **Burgenland:** Bruckneudorf, without date, J. Obenberger leg., 1 ♀ (NMPC); Illmitz, v.1922, Baderle leg., 1 ♀ (NHMW); same locality, 22.v.1981, H. Schaeflein leg., coll. R. Schrepfer, 1 ♂ (SMNS); Jois, 30.vii.1955, without collector’s name, 1 ♂ (ZMHB); Lake Neusiedl (Neusiedler See), vi.1927, W. Prock leg., 1 ♂ (NHMW); same locality, 1941, R. Zimmermann leg., coll. Herb. Schmidt, 1 ♂ (SMTD); same locality, 17.vii.1955, without collector’s name, 1 ♂ (ZMHB); same locality, 30.vi.1956, without collector’s name, 1 ♂ (ZMHB); same locality, 1962, Karl Kusdas leg., 1 ♀ (NHMW); same locality, 17.v.1965, Folwaczny leg., 1 ♀ (SMNS); same locality, 22.iv.1967, Baderle leg., 1 ♂ (NHMW); same locality, 20.iv.1976, Scheidl leg., 1 ♂ (NHMW); same locality, 26.v.1978, Probst leg., 1 ♀ (NHMW); Podersdorf am See, 1.v.1932, without collector’s name, 1 ♂ (NHMW); same locality, 19.-31.vii.1956, R. Papperitz leg., coll. R. Papperitz, 1 ♂ (SMNS); same locality, 1.v.1974, Kirschenhofer leg., 1 ♀ (NHMW); Rosalia, 19.vii.1960, Dr. Hermann Vogt leg., coll. Hessen Sammlung, 1 ♂ (SMFD); Zurndorf, without date, H. Franz leg., 1 ♀ (NHMW); **Carinthia:** Pischeldorf, without date, Moosbrugger leg., 1 ♀ (NHMW); Steinteld, without date and collector’s name, coll. R. Köstlin 1987, 1 ♀ (SMNS); same locality, without date and without collector’s name, 1 ♀ (NHMW); without locality without date and collector’s name, 1 ♀ (ZMHB); **Lower Austria:** Achau, without collector’s name, 3.vii.1926, 1 ♂ (NHMW); Amstetten, without date, Moosbrugger leg., 1 ♂ (NHMW); Anninger (Mt.), 16.vi.1997, 1 ♀ (NHMW); Bad Vöslau, Danube, without date and collector’s name, 2 ♀ (NHMW); same locality, without date and collector’s name, 1 ♂ (NHMW); Wien-Umgebung [env.]; without date, Th. V. Wänka leg., 2 ♀ (NHMW); same locality, without date and collector’s name, coll. W. H. Muche, 1 ♂ (SMTD); same locality, without date and collector’s name, coll. V. Zoufal, 1 ♀ (MZMB); same locality, without date and collector’s name, ex coll. Winkler, 1 ♂ (MHNG); Bisamberg, without date, Dr. Minarz leg., 1♂ (NHMW); same locality, 1925, Zigeuner leg., ex coll. Winkler, 1 ♀ (MHNG); same locality, 14.v.1950, Dr. H. Lechner leg., 1 ♀ (NHMW); Ellender Wald, v.1928, Zigeuner leg., ex coll. Winkler, 1 ♂ (MHNG); Felixdorf, 21.v.1974, Ulbrich leg., 1 ♂ (SMNS); Gramatneusiedl, without date, Baderle leg., 1 ♂ (NHMW); same locality, without date, Pachole leg., 1 ♀ (NHMW); Guntramsdorf, 1989, without collector’s name, 1 ♀ (NHMW), same locality, 10.iv.1989, without collector’s name, sand pit (Sandgrube), 1 ♂, 1 ♀ (NHMW); Hadersdorf am Kamp, without date and collector’s name, 1 ♀ (NMPC); Hainburg an der Donau, without date, Mader leg., 1 ♂ (NHMW); Herzogenburg, Nied (river), without date and collector’s name, 3 ♂, 4 ♀ (NHMW); Hohe Wond, without date and collector’s name, coll. Wingelmüller, 1 ♀ (NHMW); Kamptal (wine area), Dr. Minarz leg., coll. Dr. Minarz, 1 ♂, 1 ♀ (NHMW); Kampiche, 1.vii.1969, Wechelg leg., 1 ♀ (NHMW); Krems an der Donau, 11.vi.1924, I. Bachinger leg., 1 ♂ (NHMW); same locality, without date, I. Bachinger leg., 1 ♀ (NHMW); Kampichel, vii.[19]69, Wechelg leg., 1 ♀ (NHMW); Langenzersdorf, v.1965, without collector’s name, 1 ♀ (NHMW); Leithagebirge, without date, Mader leg., 1 ♀ (ZMHB); same locality, without date and collector’s name, coll. Kaufmann, 1 ♂, 1 ♀ (NHMW); Marchfeld, without date and collector’s name, 1 ♀ (SMNS); Melk, viii.1917, without collector’s name, 1 ♂ (NHMW); Mödling, Dr. H. Lechner leg., 1 ♂ (NHMW); Neu’enkbach [not located], 19.v.1924, Ajtai Kovach leg., 1 ♂ (HNHM); Fischa (river), without date and collector’s name, 1 ♀ (NHMW); Oberweiden (Weiden an der March), without date, Hammer leg., 1 ♂ (NHMW); Perchtoldsdorf, Czerny leg., 1 ♀ (NMPC); Retzbach, 4.viii.[19]32, Hedicke leg., coll. P.v.d. Wiel, 1 ♂ (ZMAN); Sankt Pölten, without date, Dr. H. Lechner leg., 1 ♂ (NHMW); Stiefern, without date, Dr. Minarz leg., coll. Dr. Minarz, 1 ♂ (NHMW); Traiskirchen, 1 ♂ (NHMW); Wachau, Dürnstein village, without date, I. Bachinger leg., 1 ♂ (NHMW); Wachau, without date, J. Moosbrugger leg., 1 ♀ (NHMW); Weidling (Klosterneuburg), without date, Walter leg., 2 ♂, 2 ♀ (NHMW); Weyersdorf near Dunkelsteinerwald, without date and collector’s name, 1 ♂ (NHMW); **Salzburg:** Pinzgau west, Habachtal (valley), 15.vii.1982, R. Belert leg., 1 ♂ (SMNS); **Styria:** Kulm am Zirbitz, without date and collector’s name, 1 ♂ (ZMHB); Graz, 5.v.1925, coll. Praxmarer, 1 ♀ (NHMW); Lannach, V. Konschegg leg., coll. Konschegg, 1 ♂, 1 ♀ (NHMW); Marburg, without date and collector’s name, coll. Konschegg, 1 ♂ (NHMW); Neudorf bei Passail, without date and collector’s name, coll. Konschegg, 1 ♂ (NHMW); Purgstall, 14.vi.1973, Ressl leg., pitfall trap, 1 ♂ (NHMW); Stainztal, without date and collector’s name, coll. Konschegg, 1 ♂ (NHMW); **Vienna:** viii.1933, Prock leg., 1 ♀ (NHMW), same locality, 1937, without collector’s name, coll. G. Paganetti Hummler, 1 ♂, 1 ♀ (ZMAN); same locality, without collector’s name, viii.[19]62, coll. Schneider-Kelch., general coll., 1 ♀ (SDEI); same locality, without collector’s name, same locality, 16.viii.1988, without collector’s name, 1 ♀ (NHMW); same locality, without date, Carl Mandl leg., 1 ♀ (NMPC); same locality, without date, G. Reineck leg., coll. Bosch, 1 ♂ (SMFD); same locality, without date, Kelfer leg., 1 ♀ (NMPC); same locality, without date, Klees leg., 1 ♂ (ZSM); same locality, without date, Kracik leg., 1 ♂ (NMPC); same locality, without date, O. Leonhard leg., general coll., 1 ♂ (SDEI); same locality, without date, coll. Reitter, 1 ♂ (NMPC); same locality, without date, Schlereth leg., 2 ♂, 1 ♀ (NHMW); same locality, without date, Türkensch leg., coll. Wingelmüller, 1 ♂ (NHMW); same locality, without date, Walter leg., 1 ♀ (NHMW); same locality, without date and collector’s name, coll. Prof. Noesske, on bird, 1 ♂ (SMTD); same locality, without date and collector’s name, ex coll. Winkler, 1 ♀ (MHNG); same locality, without date and collector’s name, coll. Bachofen, ex coll. Winkler, 1 ♂ (MHNG); same locality, without date and collector’s name, coll. Helter, 1 ♂ (SMTD); same locality, without date and collector’s name, 2 ♂ (ZMHB); Dornbach, without date and collector’s name, coll. Kaufmann, 1 ♀ (NHMW); same locality, without date and collector’s name, 1 ♀ (NHMW); Gartenhotel Altmannsdorf, 12.vii.1958, Scheidl leg., 1 ♂ (NHMW); Kahlerberg (Mt.), 12.v.1926, without collector’s name, 1 ♂ (NHMW); Kuchelau harbour, without date and collector’s name, coll. Muhr, 1 ♀ (NHMW); Oberlaa, v.1974, 1 ♀ (NHMW); Prater (public park), without date, Carl Mandl leg., 1 ♂ (NMPC); Stammersdorf, v.1965, 2 ♂, 1 ♀ (NHMW); same locality, without date, Dr. H. Lechner leg., 1 ♂ (NHMW); same locality, without date, Mader leg., 1 ♀ (NHMW); same locality, without date, Käufel leg., 1 ♂, 1 ♀ (NHMW); Wienerwald highlands, without date, K. Klein leg., 1 ♂ (NMPC); same locality, without date and collector’s name, 1 ♂ (ZMHB), same locality, Peilstein, without date and collector’s name, 2 ♂, 2 ♀ (NHMW); **Vorarlberg**, Feidkirch, without date, Moosbrugger leg., 1 ♀ (NHMW); **Austria without specific locality:** without date and collector’s name, coll. Heller, 1 ♂, 1 ♀ (SMTD); without date and collector’s name, coll. Milch, 1 ♀ (NHMW); without date and collector’s name, coll. Wustnei, 1 ♂ (ZMUC); North Austria, without date and collector’s name, ex coll. Grabowski, 1 ♂ (HNHM);

**Belgium:** Huy, 1906, without collector’s name, coll. Pfaft, 1 ♀ (SMFD); Namur, without date, Muche leg., 1 ♂ (SMTD); without locality, 1906, without collector’s name, coll. Pfaft, 1 ♂ (SMFD); Von Halfern Park (South-West Aachen), v.1876, coll. Rhine, 1 ♀ (ZMHB);

**Bosnia and Herzegovina:** **Federation of Bosnia and Herzegovina:** Bosansko-Grahovo, 800 m, vii.1998, Krätschmer leg., 1 ♂ (SMNS); Breza, without date, Hlisnikowski leg., 1 ♀ (NMPC); Čelić, without date, Zoufal leg., 1 ♀ (NMPC); Klekovača (Mt.), 1892, Beck leg., 1 ♂, 1 ♀ (NHMW); Makljen-Pass, 7.v.1902, O. Leonhard leg., general coll., 1 ♀ (SDEI); Majevica (Mt.), without date and collector’s name, 1 ♀ (NHMW); same locality, without date and collector’s name, 1 ♂, 1 ♀ (NHMW); Mostar, without date, Brydl leg., 1 ♂ (NMPC); same locality, without date, Jak. Schmülzer, V.H. Konschegg leg., coll. Konschegg, 1 ♀ (NHMW); same locality, without date, Scheibel leg., coll. Pinker, 1 ♂ (NHMW); same locality, without date, Stepanek leg., 1 ♂ (NMPC); same locality, without date, Zoufal leg., 1 ♂ (NMPC); same locality, without date and collector’s name, coll. V. Zoufal, 1 ♂ (MZMB); Petrovac, without date, Schmidl leg., 1 ♀ (NHMW); Popovo polje [Popovo field] valley, Kotezi, 25.-26.v.1991, V. Zieris leg., 1 ♀ (JRUC); Sarajevo, without date and collector’s name, coll. Winglemüller, 1 ♂, 1 ♀ (NHMW); same locality, without date and collector’s name, 3 ♂, 1 ♀ (NMPC); Treskavica (Mt.), without date and collector’s name, coll. W. H. Muche, 1 ♂ (SMTD); Zavidovići, 11.v.1907, Kendi leg., 1 ♀ (HNHM); same locality, vi.1907, Kendi leg., 1 ♂ (HNHM); Zenica (Zennrico), without date, A. Schatzmayr leg., coll. O. Leonhard, 1 ♀ (SDEI); **Republika Srpska:** Bileća, without date, Grabowski leg., 1 ♀ (HNHM); Bjelašnica (Mt.), 17.vii.1988, Beck leg., 1 ♂ (NHMW); same locality, 17.vii.1988, without collector’s name, 1 ♀ (NHMW); same locality, 2000 m, vi.1984, Krätschmer leg., 1 ♂ (SMNS); same locality, without date, Diener leg., coll. J. Fodor, 1 ♀ (HNHM); same locality, without date, J. Fodor leg., 1 ♂ (HNHM); same locality, without date, O. Leonhard leg., general coll., 4 ♂, 4 ♀ (SDEI); same locality, without date, Zeithammer leg., 1 ♀ (NHMW); same locality, without date and collector’s name, 1 ♂ (NHMW); same locality, without date, O. Leonhard leg., coll. Prof. Noesske, 1 ♂ (SMTD); Trebinje, 12.vi.1931, Dr. J. Fodor leg., coll. M. Nattan, 1 ♂, 2 ♀ (HNHM); same locality, without date, Vašíček leg., coll. K. Mazura, 1 ♀ (MZMB); same locality, without date and collector’s name, coll. Burghauser, Mahler, Waka, Fleischer, Absolon, 1 ♂ (MZMB); same locality, without date and collector’s name, coll. M. Nattan, 1 ♀ (HNHM); same locality, without date and collector’s name, coll. Reitter, 1 ♂ (HNHM); Višegrad, without date, Zoufal leg., 1 ♀ (NMPC); Volujak (Mt.), 1902, O. Leonhard leg., general coll., 5 ♂, 1 ♀ (SDEI); same locality, without date, O. Leonhard leg., coll. Prof. Noesske, 1 ♂, 1 ♀ (SMTD); same locality, without date and collector’s name, coll. W. H. Muche, 1 ♀ (SMTD); same locality, without date and collector’s name, 1 ♀ (SMTD); Zelengora, 1400 m, v.1977, D. Bare, Krätschmer leg., 1 ♀ (SMNS); **Bosnia without specific locality:** without date, Derodt leg., 1 ♀ (NMPC); without date and collector’s name, 1 ♂ (NMPC); without date and collector’s name, 1 ♀ (HNHM); Dalmatia, without date and collector’s name, 1 ♂ (ZMHB); Bgman [not located], without date, O. Leonhard leg., general coll., 1 ♂ (SDEI); Ksicna [not located], without date, O. Leonhard leg., general coll., 1 ♀ (SDEI); **Hercegovina without specific locality:** 1993, Hawelka leg., 1 ♂, 2 ♀ (NHMW); without date, Reitter leg., coll. R. Formanek, 1 ♂ (MZMB); Domanovič [probably Domanovići], 22.-24.vii.1930, O. Holik leg., 1 ♂ (SMTD);

**Bulgaria:** **Blagoevgrad prov.:** Alí Botúsh or Alibotush (Mt.), 20.vii.1936, E. Csiki leg., 1 ♀ (HNHM); same locality, without date, Hlisnikowski leg., 1 ♂ (NMPC); same locality, without date, Mařan & Táborský leg., 1 ♂ (NMPC); Petrich, without date, Pfeffer leg., 1 ♂ (NMPC); **Burgas prov.:** Kiten, 10.vii.1973, E. Kismarja leg., 1 ♀ (HNHM); same locality, without date, J. Mička leg., 1 ♂ (JRUC); Lozenets, 19.vii.1971, J. Boháč leg., 1 ♂ (JRUC); same locality, 29.vi.1997, M. Šárovec leg., 1 ♂ (JRUC); Nesebar, 12.vi.1962, K. Ermisch leg., 1 ♀ (ZMHB); same locality, 4.vi.1964, K. Ermisch leg., 1 ♀ (ZMHB); same locality, 7.vi.1964, K. Pospíšil leg., 1 ♂ (MZMB); same locality, 9.vi.1964, K. Pospíšil leg., 1 ♀ (MZMB); same locality, 20.vi.-6.vii.1965, K. Ermisch leg., 1 ♂ (ZMHB); same locality, 3.vi.1967, K. Ermisch leg., 1 ♂ (ZMHB); same locality, 10.vi.1967, K. Ermisch leg., 1 ♀ (ZMHB); Nesebar (on the Black Sea coast), below the mill, 18., 25.v.1975, H. Schindler leg., 2 ♂ (SMNS); Pomorie, 3.-5.vi.1982, H. Wendet leg., 1 ♂ (ZMHB); Pomoje dintouni, 26.iv.1973, F. Battoni leg., 1 ♀ (MHNG); Primorsko, 20.ix.1975, P. Pecina leg., 1 ♀ (JRUC); Sozopol, viii.1965, Josef Král leg., 1 ♂, 1 ♀ (JRUC); same locality, 1971, J. Král leg., 1 ♀ (JRUC); same locality, without date, J. Jelínek leg., 2 ♀ (NMPC); same locality, without date, Hlisnikowski leg., 1 ♀ (NMPC); Slančev Brjag, 25.viii.1976, K. Pospíšil leg., tons of rubble, 1 ♀ (MZMB); same locality, without date, Dr. A. Svozil leg., 1 ♂ (SMNS); **Dobrich prov.**, Kamen Brjag, 11.vii.1984, J. Růžička leg., 1 ♀ (JRUC); **Gabrovo prov.**, Tryavna, v.-vi.1912, M. Hilf leg., coll. O. Leonhard, 1 ♀ (SDEI); **Pazardzhik prov.**, Isperihovo village, 17.v.2009, V. Zieris leg., 1 ♀ (JRUC); **Plovdiv prov.**, Asenovgrad, without date, Balog leg., 1 ♂ (HNHM); **Varna prov.:** 22.vii.1936, without collector, 1 ♂ (NHMW); same locality, 1994, Peez leg., 2 ♂, 2 ♀ (NHMW); same locality, without date and collector’s name, 1 ♀ (NHMW); Galata beach, without date, J. Balogh leg., 1 ♂ (HNHM); Golden Sands, 3.ix.1965, P. Sedina leg., in dry grass at noon, 1 ♀ (JRUC); Varna beach, without date, Balogh leg., 1 ♀ (HNHM); **Shumen prov.**, Mandra, Fuss leg., 1 ♂, 1 ♀ (NMPC); **Sofia:** v.1902, without collector’s name, 1 ♀ (ZMHB); Lyulin (residential complex in Sofia), without date, Hanuš leg., 1 ♂ (NMPC); **Stara Zagora prov.**, Kazanlak, 11.-12.ix.1977, Hieke & Ublig leg., 1 ♀ (ZMHB); **Veliko Tarnovo prov.**, Veliko Tarnovo, 18.vii.1965, Palle Jorum leg., 1 ♀ (ZMUC); **Southern Bulgaria:** Rhodope (Mts.), Bachkovo Monastery, 15.iii.1977, D. W. Wrase leg., 1 ♂ (ZMHB); Zvezdec village near Strandzha (Mts.), without date, I. Smatana leg., 1 ♂, 2 ♀ (JRUC); **Bulgaria without a specific locality:** Madaia [not located], 1928, Biro leg., 1 ♂ (HNHM); vii.1975, without collector’s name, 1 ♂ (ZMHB); iv.1989, S. Bečvář leg., 3 ♂, 2 ♀ (JRUC); without date and without collector’s name, 1 ♂ (JRUC); without date and collector’s name, 2 ♂ (NMPC); Dabovec env., Arda challet [not located], without date, steppe, Hůrka leg., 1 ♂ (NMPC);

**Croatia:** **Dalmatia:** “Dalmatia”, without date, Al. Smolka leg., 1 ♀ (NMPC); same locality, without date, J. Vaca leg., 1 ♂ (NMPC); same locality, without date, Kust leg., 1 ♂ (SDEI); same locality, without date, Všetečka leg., 2 ♂ (NMPC); same locality, without date and collector’s name, coll. Fry, 1 ♂, 1 ♀ (BMNH); same locality, without date and collector’s name, coll. Fry, 1 ♀ (BMNH); same locality, without date and collector’s name, coll. K. Hanel, 1 ♂ (SMTD); same locality, without date and collector’s name, coll. Pinker, 1 ♀ (NHMW); same locality, without date and collector’s name, 2 ♀ (MHNG); same locality, without date and collector’s name, 1 ♂, 2 ♀ (NMPC); same locality, without date and collector’s name, 1 ♂ (ZMUC); Brač island, without date, Ad. Hoffmann leg., coll. Prof. Noesske, 1 ♂ (SMTD); Kaštela, without date and collector’s name, 1 ♂ (NHMW); Zennrico [not located], without date, A. Schatzmayr leg., coll. O. Leonhard, 1 ♀ (SDEI); **Dubrovnik-Neretva County:** Dubrovnik (South Dalmatia), 3.v.1981, M. Boneß leg., 1 ♂ (SMNS); same locality, without date and collector’s name, 1 ♀ (NMPC); Opuzen, 29.iv.1975, G. J. Slob leg., 1 ♂, 1 ♀ (ZMAN); Trpanj, without date, Fleischer leg., 1 ♀ (NMPC); Korčula island, v.1902, Gabriel leg., coll. Bosch, 1 ♀ (SMFD); Metković, without date, Fleischer leg., 1 ♂ (NMPC); Hvar island: without date, Kouřil leg., 1 ♂ (NMPC); without date, Hirst leg., 2 ♀ (NMPC); Starigrad, Verbosca (settlement), 13.iv.1929, P. Shulze leg., 1 spec. (ZMHB); Sućuraj, without date and collector’s name, 2 ♀ (NHMW); Zadar, without date, Bocagnazzo leg., 1 ♀ (NMPC); same locality, without date and collector’s name, coll. E. Grundmann, 1 ♀ (NHMW); **Istria County:** without date, without date and collector’s name, coll. Reitter, 1 ♂ (JRUC); same locality, without date and collector’s name, coll. W. H. Muche, 1 ♀ (SMTD); same locality, without date and collector’s name, coll. Reitter, 1 ♀ (HNHM); same locality, without date and collector’s name, 1 ♂ (SMTD); Fuskulin (10 km South Prec), 22.vi.2003, O. Merkl leg., swept and singled, 1 ♂ (HNHM); Isola, 23.vii.1969, S. Horvatovich leg., 1 ♂ (HNHM); Poreč, 29.vi.1920, Devring leg., coll. D. Liebegott, 1 ♀ (SMFD); Pula, 20.viii.1981, T. Klement leg., 1 ♂, 1 ♀ (JRUC); same locality, 21.vii.1982, T. Klement leg., 1 ♀ (JRUC); same locality, 11.xi.1877, Krauss leg., 1 ♀ (NHMW); same locality, without date, Heyrovský leg., 1 ♀ (NMPC); same locality, without date, Kouřil leg., 1 ♀ (NMPC); same locality, without date, Lichtenckert leg., 1 ♂ (HNHM); same locality, without date, Schlettau leg., 1 spec. (NHMW); same locality, without date, Schuster leg., 1 ♀ (NMPC), same locality, without date and collector’s name, 1 ♂ (JRUC); same locality, without date and collector’s name, coll. Kaufmann, 2 ♂, 1 ♀ (NHMW); same locality, without date and collector’s name, 2 ♀ (NHMW); same locality, without date and collector’s name, 2 ♂, 1 ♀ (NMPC); Noriaem [not located], without date and collector’s name, 1 ♀ (NHMW); Rovinj, 4.vi.1960, M. A. Ertel leg., 1 ♀ (SMNS); Vodnjan (Dignano), without date and collector’s name, 1 ♀ (JRUC); **Karlovac County**, Karlovac, without date, Victor Stiller leg., coll. Stiller, 1 ♀ (HNHM); **Lika-Senj County:** Otočac, without date, Jan Obenberger leg., 2 ♀ (NMPC); Senj, 30.vi.1905, Biro leg., 1 ♂ (HNHM); **Lika-Senj County/ Zadar County**, Pag island, without date and collector’s name, 1 ♂ (NMPC); **Primorje-Gorski Kotar County:** Bakar (Buccari), 16.v.1911, without collector’s name, coll. Schaltz, 1 ♂ (ZMUC); Crikvenica, without date and collector’s name, coll. Mihók, 1 ♂ (HNHM); Fužine, without date and collector’s name, coll. Mihók, 1 ♂ (HNHM); Fužine, coastland, 1906, M. Hilf leg., coll. O. Leonhard, general coll., 1 ♂ (SDEI); Krk island, without date and collector’s name, 3 ♀ (NHMW); same locality, 1977, Mader leg., 1 ♀ (ZMHB); Krk city, viii.1968, without collector’s name, coll. J. Dezort, 1 ♂ (MZMB); Krk city (surrounding area), 20.ix.2007, Erwin Holzer leg., 1 ♀ (EHOC); Mali Lošinj (Lussinpiccolo), without date, Majersky leg., 1 ♀ (NHMW); Novi Vinodolski, without date and collector’s name, coll. Mihók, 1 ♂ (HNHM); Punat, viii.1968, without collector’s name, coll. I. Grulich, 2 ♂ (MZMB); Rijeka (or Fiume), without date, Victor Stiller leg., coll. Stiller, 1 ♀ (HNHM); same locality, without date and collector’s name, 1 ♂ (NMPC); Rab island, without date, Fleischer leg., 1 ♂ (NMPC); **Split-Dalmatia County:** Dugopolje, 1.-3.v.2009, L. & V. Zieris leg., 1 ♂ (JRUC); Kaštel Sućurac, without date, Novak, Kouřil leg., 1 ♂ (NMPC); Klis Fortress near Split, 1.iv.1973, R. Grimm leg., 1 ♂ (SMNS); Omiš (Almissa), without date, Horvath leg., 1 ♂ (HNHM); Salona near Solin, without date, Hoffmann leg., 1 ♂ (NMPC); Sinj, without date, Klimesch leg., 1 ♀ (SMNS); Split (Spalato), iv.[19]28, D. Heberdey leg., coll. Praxmarer, 1 ♂ (NHMW); same locality, 6.v.1938, Dr. Feige leg., 1 ♀ (ZMHB); same locality, 22.v.1975, G. J. Slob leg., 1 ♀ (ZMAN); same locality, without date, Karaman leg., coll. Hauser, 4 ♂ (NHMW); Grenzgebiet, Kralu [not located], 1886, Gerlach leg., 1 ♀ (ZMHB); Vis island (Lissa), 1890, Steind leg., 1 ♀ (NHMW); **Velebit mountaine range**, 1500 m, vi.1967, without collector’s name, coll. Stegmann, 1 ♂ (SMNS); same locality, vi.1968, W. Back leg., 1 ♂, 1 ♀ (NHMW); **Zagreb (historic name Agram)**, without date, O. Leonhard leg., general coll., 1 ♂ (SDEI);

**Czech Republic:** **Moravian-Silesian Reg.**, Herčivald or Herzogwald [locality no longer exists], 1900, without collector’s name, coll. K. Mazura, 2 ♀ (MZMB); **Olomouc Reg.:** Prostějov, without date, Kouřil leg., 1 ♂ (NMPC); Weisskirchen [Hranice na Moravě], without date and collector’s name, 1 ♀ (NHMW); **South Bohemian Reg.:** Strakonice, without date, Šípek leg., 1 ♂ (NMPC); **South Moravian Reg.:** Adamov, without date, J. Fleischer leg., 1 ♂ (NMPC); Brno, 8.v.1921, without collector’s name, 1 ♂ (MZMB); same locality, v.1942, Rouscher leg., 1 ♂ (MZMB); same locality, without date, J. Fiala leg., 1 ♀ (MZMB); same locality, without date, Kafka leg., 1 ♀ (JRUC); same locality, without date, Fleischer leg., 2 ♀ (NMPC); same locality, without date and collector’s name, 1 ♂ (NMPC); Brno-Ivanovice, without date and collector’s name, 1 ♂ (NMPC); same locality, without date, Kouřil leg., 1 ♀ (NMPC); Brno-Řečkovice, without date, Formánek leg., coll. R. Formánek, 3 ♀ (MZMB); Bučovice, without date and collector’s name, 1 ♂ (NHMW); Čejč village (in Hodonín Dist.), v.1943, Rouscher leg., 1 ♀ (MZMB); same locality, without date, J. Bechyně leg., 1 ♀ (JRUC); same locality, without date, V. Zavadil leg., 2 ♀ (NMPC); same locality, without date, O. Kodym leg., 1 ♂ and 3 ♀ (NMPC); same locality, without date, Pfeffer leg., 2 ♂ (NMPC); Hodonín, without date, J. Baumert leg., 2 ♀ (NMPC); same locality, without date, Hlisnikovský leg., 1 ♂ (NMPC); same locality, without date and collector’s name, 1 ♂, 1 ♀ (NMPC); Hustopeče, without date, J. Fleischer leg., 2 ♀ (NMPC); Ivančice, without date and collector’s name, coll. Fleischer, 1 ♂ (NMPC); Mutěnice, 13.viii.1963, J. Bechyně leg., 1 ♀ (JRUC); same locality, without date, J. Bechyně leg., 1 ♂ (JRUC); Pálava, without date, J. Fleischer leg., 1 ♂ (NMPC); Sokolnice, without date, J. Fiala leg., coll. J. Fiala, 1 ♀ (MZMB); Stará hora, Novosedly, without date, A. Fleischer leg., 1 ♂ (NMPC); same locality, without date and collector’s name, 1 ♂, 1 ♀ (NMPC); Tábor, without date and collector’s name, coll. Burghauser, Mahler, Waka, Fleischer, Absolon, 1 ♀ (MZMB); Uherčice, 1941, without collector’s name, coll. J. Matoušek, 1 ♀ (MZMB); Znojmo, 1935, without collector’s name, coll. J. Matoušek, 1 ♂ (MZMB); Židlochovice, 19.viii.1914, coll. J. Dezort, 1 ♂ (MZMB); **Vysočina Reg.**, Červená Řečice, without date, Stiburek leg., 1 ♂ (NMPC); **Zlín Reg.:** Bílovice, without date, M. Chvojka leg., 1 ♂ (NMPC); Chvalčov village (in Kroměříž Dist.), 2.vii. [year not provided], L. Cap leg., 1 ♂ (SMNS); Koryčany, without date, O. Kodym leg., 1 ♂ (NMPC); Luhačovice, without date and collector’s name, 1 ♂, 1 ♀ (NMPC); Uherský Brod, vii.1927, W. Prock leg., 1 ♀ (NHMW); same locality, without date, W. Prock leg., 1 ♂ (NHMW);

**France:** **Alsace Reg.:** Souffelweyersheim, vi.[19]53, Stras Bou leg., 1 ♀ (MHNG); Teghllo [not located], without date, coll. Heyden, 1 ♂ (ZSM); without specific locality, without date and collector’s name, coll. W. H. Muche, 1 ♂ (SMTD); **Aquitaine Reg.:** Bordeaux, iv.1943, F. Ermisch leg., 1 ♂, 1 ♀ (ZMHB); same locality, v.1943, F. Ermisch leg., 1 ♂ (ZMHB); Col du Soulor mountain pass, 1892, D. Nodis leg., coll. Bertin, 1 ♀ (MHNG); same locality, without date, D. Nodis leg., 1 ♀ (MHNG); Hendaye, viii.1889, P. de Borre leg., 1 ♀ (MHNG); Léon, without date and collector’s name, coll. W. H. Muche, 1 ♂ (SMTD); **Auvergne Reg.:** Saint-Ferréol-d’Auroure, without date and collector, coll. Achard, 1 ♂ (NMPC); **Basse-Normandie Reg.:** Trouville-sur-Mer, without date and collector’s name, coll. Kraatz, general coll., 1 ♂ (SDEI); **Bigorre Reg.:** Callian, without date, coll. Sharp, 2 ♂ (BMNH); **Brittany Reg.:** Morbihan Dep., without date, Pora Louss leg., 1 ♀ (MHNG); Noirmoutier island, iv.1961, Dr. Breuning leg., ex coll. Breuning, 1 ♂, 1 ♀ (SMTD); same locality, without date and collector’s name, ex coll. Breuning, 18 ♂, 6 ♀ (SMTD); Saint-Gilles-les-Bois, 27.iii.1978, Kiener leg., coll. S. Kiener, 1 ♂ (MHNG); **Centre Reg.:** Amboise, 28.v.1925, without collector’s name, coll. Rosenberg, 1 ♀ (ZMUC); Brezolles, without date, Achard leg., 2 ♀ (NMPC); Cher Dep., Avord, 18.vi.1941, Folwaczny leg., 1 ♀ (SMNS); Tours, without date, D. Ludy leg., 1 ♀ (HNHM); **Champagne-Ardenne Reg.:** Langres plateau, without date and collector’s name, 1 ♀ (NMPC); Preifs [not located], without date and collector’s name, coll. W. H. Muche, 5 ♂, 3 ♀ (SMTD); Prosnes, Martin Heyne, without date, J. Fleischer leg., 1 ♂ (NMPC); Reims, without date and collector’s name, coll. Johannes Werner Schmidt, 1 ♀ (SMTD); same locality, without date and collector’s name, 1 ♂, 1 ♀ (NMPC); without specific locality, 1917, without collector’s name, coll. Detzner, 2 ♀ (SMTD); **Corsica island:** Arro, without date, G. Reineck leg., coll. Bosch, 1 ♂ (SMFD); Corte, 28.v.1912, E. Christeller leg., 1 ♂ (ZMHB); Folelli, 1905, O. Leonhard leg., general coll., 1 ♂ (SDEI); Monte Rotondo, without date and collector’s name, coll. Hanser, 1 ♀ (NHMW); without specific locality, 23.v.1912, E. Christeller leg., 1 ♂ (ZMHB); **Franche-Comté Reg.:** Jura Dep., Staffelberg (Mt.), 22.v.1952, Eckerlein leg., 1 ♂, 1 ♀ (NHMW); Jura Dep., 18.v.1952, coll. Bertin, 1 ♀ (MHNG); Vosges Mts. (South), v.1916, Muller leg., coll. Prof. Noesske, 1 ♂ (SMTD); **Île-de-France Reg.:** Fontainebleau, without date, J. Achard leg., coll. Achard, 1 ♂ (NMPC); Paris, without date and collector’s name, 1 ♀ (MNCN); Provins, 31.iii.1948, P. Bureau leg., coll. P. Bureau, 1 ♀ (SMNS); same locality, without date and collector’s name, coll. Andrewes, 1 ♂ (BMNH); Seine (river), without date and collector’s name, coll. K. Mazura, 1 ♂ (MZMB); Thoiry, 10.ix.1929, K. Ponov leg., 1 ♂ (ZMUM); same locality, 10.vi.1931, K. Ponov leg., 1 ♂ (ZMUM); **Languedoc-Roussillon Reg.:** Agde, 4.-15.iv.1982, Rieger leg., 1 ♂ (SMNS); Aigues-Mortes, 14.i.1930, J. Therond leg., coll. E. Jagemann, 1 ♀ (MZMB); Aimargues, 15.v.1903, Cheviny, 3 ♂ (MHNG); Aude Dep.: Leucate, 1968, J. Hamon leg., 1 ♂ (MHNG); same locality, 1.viii.1970, J. Hamon leg., 1 ♂ (MHNG); same locality, 15.-16.vi.1973, C. v. Nidek leg., 1 ♀ (ZMAN); same locality, 10.vii.1984, J. Hamon leg., 1 ♀ (MHNG); Quillan, 5.v.1955, J. Th. Skovgaard leg., 1 ♀ (ZMUC); Bagnols-sur-Cèze, 18.iv.1981, Döser leg., 1 ♂, 1 ♀ (SMNS); Canet-en-Roussillon (or Canet Plage), 15.-16.v.1973, G. J. Slob leg., 2 ♂, 1 ♀ (ZMAN); Gard Dep.: Aigues-Mortes, 1.-8.iv.1972, P. Poot leg., 1 ♀ (ZMAN); Gallician, without date, H. Perrot leg., 1 ♂ (MHNG); Le Grau-du-Roi, 1.-8.iv.1972, P. Poot leg., 1 ♀ (ZMAN); Uzès, 29.ix.1949, M. Curti leg., 1 ♀ (MHNG); Aude Dep.: Marsa, 25.-26.v.1972, Schawaller leg., 1 ♀ (SMNS); Montpellier, without date, K. Neumann leg., 1 ♂, 1 ♀ (SMFD); Nimes, 28.vi.1925, J. Therond leg., 1 ♂ (NHMW); same locality, without date, J. Therond leg., 1 ♂ (NHMW); Palavas-les-Flots, 27.iv.1949, Eyndboven leg., coll. P. v. d. Wiel, 1 ♂ (ZMAN); Perpignan, 10.vi.1976, G. J. Slob leg., 1 ♀ (ZMAN); Pont du Gard, 20.vii.1924, J. Therond leg., 1 ♂ (ZMHB); same locality, without date, J. Therond leg., coll. E. Jagemann, 1 ♂ (MZMB); Port-Vendres, vii.1975, Kiener leg., coll. S. Kiener, 1 ♀ (MHNG); same locality, viii.1975, Kiener leg., coll. S. Kiener, 1 ♀ (MHNG); Pyrénées (Mts), Lac d’Estom, 1989, 1 ♀ (NHMW); Pyrénées-Orientales Dep., Arles sur Tech, 270 m, 14.-20.vi.1962, P.C. Jerrard leg., 1 ♀ (BMNH); **Lorraine Reg.:** Metz, viii.1957, Lenczy leg., 1 ♀ (HNHM); Vosges Dep., without date, coll. Andrewes, 1 ♂ (BMNH); **Lower Normandy Reg.:** Calvados, without date, Dubourgais leg., coll. Pinker, 1 ♀ (NHMW); same locality, without date, Dubourgais leg., 1 spec. (NMPC); same locality, without date and collector’s name, 2 ♂ (NMPC); Mont Saint-Michel, without date, coll. Reitter, 1 ♀ (SMNS); Luberon (Mts.), Maubec, 30.iv.2003, H. & I. van Oorschot leg., 1 ♀ (ZMAN); **Midi-Pyrénées Reg.:** Ariège, v.1965, Delestelas, Puig de l’Estela [not located], without collector’s name, 1 ♀ (MHNG); Gers, Terraube, without date, 1 ♀ (NMPC); Ornolac-Ussat-les-Bains, without date, Laznicka leg., 1 ♂ (NMPC); **Nord-Pas-de-Calais Reg.:** Calais, without date and collector’s name, 1 ♀ (BMNH); Cambrai, v.1917, without collector’s name, coll. Dr. Jhssen, 2 ♂ (ZMHB); same locality, x.1917, without collector’s name, coll. Dr. Jhssen, 1 ♀ (ZMHB); Monchy-le-Preux, without date, Achard leg., 1 ♂ (NMPC); **Picardy Reg.:** Chaumont-en-Vexin, vii.1927, without collector’s name, coll. Dr. R. Streda, 1 ♀ (HNHM); **Plateau de Langres (Langres plate)**, without date, Riedel leg., 1 ♀ (NMPC); **Poitou-Charentes Reg.:** Charente, 1946, without collector’s name, coll. Fuchs, 2 ♂, 1 ♀ (SMTD); same locality, without date and collector’s name, coll. Fuchs, 1 ♂ (SMTD); Cognac, without date and collector’s name, coll. Achard, 1 ♂ (NMPC); Vienne Dep., Morthemer, without date, L. Mesmin leg., 1 ♂ (NMPC); **Provence Reg.:** Verdon Gorge (Grand canyon du Verdon), 15.v.1985, U. Döser leg., 1 ♂ (SMNS); **Provence-Alpes-Côte d’Azur Reg.:** Alpes-Maritimes Dep., 900 m, 16.vii.1950, Berthemont & P. Bureau leg., coll. R. Kostlin, 1 ♀ (SMNS); same locality, Mandelieu-la-Napoule, 20.x.1962, C. v. Nidek leg., 2 ♂, 2 ♀ (ZMAN); Arles, Pont-de-Crau, 3.ix.1967, coll. R. Ertel Remseck, 1 ♂ (SMNS); Cannes, without date, Homeyer leg., 1 ♂ (SDEI); Castellane, vi.1911, D. Novak leg., coll. V. Zoufal, 1 ♀ (MZMB); Cotignac (3 km North of Barjols), 43°31'47"N, 6°09'00"E, 414 m, 20.iv.2006, J. Skuhrovec leg., 1 ♀ (JRUC); Étangs de Villepey (ponds), 2.iv.1913, W. Liebmann leg., general coll., 1 ♂ (SDEI), same locality, 10.iv.1913, W. Liebmann leg., general coll., 1 ♂, 1 ♀ (SDEI); French riviera, Saint-Raphael, 23.iii.1913, W. Liebmann leg., general coll., 2 ♂ (SDEI); Hyères Dep., 7.-12.v.1921, K. G. Blair leg., 1 ♂ (BMNH); same locality, D. Nodis leg., 1 ♀ (MHNG); same locality, without date, Gallmer leg., 1 ♀ (SMFD); Hyères Dep., Lattes, 26.x.1964, without collector’s name, 1 ♂ (MHNG); same locality, without date, H. Perrot leg., 1 ♂ (MHNG); Hyères Dep., Montpellier, without date and collector’s name, 1 ♂ (MHNG); La Ciotat, 30.vii.1940, G. Condrillier leg., 1 ♀ (MHNG); same locality, 28.viii.1948, without collector’s name, 1 ♂ (MHNG); La Turbie, 1927, Klynstra leg., 1 ♀ (ZMAN); same locality, without date and collector’s name, 1 ♂ (NMPC); Marseille, viii.-ix.1958, Lenczy leg., 1 ♂ (HNHM); same locality, without date, Kobelt leg., 1 ♂ (SDEI); Nice, 2.v.1891, without collector’s name, 1 ♀ (MHNG); same locality, 1883, without collector’s name, 1 ♀ (SMFD); same locality, without date, Al. Smolka leg., 1 ♀ (NMPC); same locality, without date, coll. Toumayeff, 1 ♂, 3 ♀ (MHNG); same locality, without date and collector’s name, 3 ♂ (BMNH); Peille, 17.x.1954, without collector, 1 ♂, 1 ♀ (MHNG); Saint-Laurent-du-Var, iv.1936, without collector, 3 ♀ (MHNG); same locality, 15.iii.1938, without collector’s name, 1 ♀ (MHNG); same locality, 8.iv.1939, without collector’s name, 3 ♂ (MHNG); same locality, 10.iv.1939, without collector’s name, 2 ♂, 1 ♀ (MHNG); Tarascon, 23.-25.v.1949, Roland Skovgaard leg., coll. Th. Skovgaard, 1 ♂ (ZMUC); Thorenc, viii.1965, without collector’s name, 1 ♀ (MHNG); Var Dep.: Canjuers, vii.1964, 1 ♀ (MHNG); Le Lavandou, without date, Obenberger leg., 1 ♀ (NMPC); same locality, without date and collector’s name, 1 ♀ (NMPC); Vaucluse Dep., iii.1937, without collector’s name, coll. H. Coiffait, 1 ♀ (MHNG); same locality, without date and collector’s name, 1 ♂ (MHNG); Mont Ventoux, 18.vi.1959, M. Curti leg., 1 ♂ (MHNG); same locality, 27.-31.v.1973, G. J. Slob leg., 1 ♂ (ZMAN); Vaison-la-Romaine, 19.v.1970, P. Poot leg., 1 ♂ (ZMAN); same locality, 21.v.1985, E. Ulbrich leg., coll. R. Schrepfer, 1 ♂ (SMNS); **Rhône-Alpes Reg.:** Chamonix, without date and collector’s name, coll. Leitzner, general coll., 1 ♀ (SDEI); Gex, 16.v.1901, P. de Borre leg., 1 ♂ (MHNG); Lyon, without date and collector’s name, coll. Perez Arcas, 1 ♂ (MNCN); Miribel Ain Dep., 1947, without collector’s name, 2 ♂ (MHNG); Saint-Vallier, vi.[19]66, Toumayeff leg., 1 ♀ (MHNG); same locality, viii.[19]69, Toumayeff leg., 1 ♀ (MHNG); same locality, viii.[19]76, Toumayeff leg., 1 ♂, 1 ♀ (MHNG); same locality, viii.[19]80, Toumayeff leg., 1 ♀ (MHNG); same locality, v.[19]85, Toumayeff leg., 1 ♂ (MHNG); Salève (Mt.), 7.v.1950, without collector’s name, coll. van de Gumster, 1 ♂, 1 ♀ (MHNG); same locality, 3.vi.1952, without collector’s name, coll. van de Gumster, 1 ♀ (MHNG); Savoie Dep., without date and collector’s name, 1 ♀ (MHNG); **Savoy Reg.:** without date and collector’s name, coll. Sharp, 1 ♂ (BMNH); without specific locality, without date and collector’s name, 2 ♂, 2 ♀ (BMNH); **Upper Normandy Reg.:** Fresney, Dubourgias [not located], without date, J. Fleischer leg., 1 ♂ (NMPC); **France without specific locality:** 14.vii.1927, without collector’s name, 1 ♀ (NHMW); without date and collector’s name, 2 ♀ (NMPC); Galia, without date and collector’s name, 1 ♀ (SMTD); South France: Senazaire, Solsales [not located], 14.vi.1948, 2 ♀ (MHNG); without specific locality, 25.iv.1962, Hermann Vogt leg., Hessen collection, 1 ♂ (SMFD); without specific locality, 24.vi.1962, Hermann Vogt leg., Hessen collection, 1 ♀ (SMFD);

**Georgia:** **Abkhazia:** Gagra, sea coast, 2.-3.ix.1977, Belov leg., 1 ♀ (ZMUM); New Athos, 24. x.1932, B. Rodendorf leg., 1 ♀ (ZMUM); **Samtskhe-Javakheti Reg.:** Bakuriani, without date, J. Mařan leg., 1 ♂ (NMPC); same locality, 15.vi.1910, Mlokos’vich leg., 1 ♀ (ZMAS); Kutaisi, 26.vi. 1898, Deryugin leg., 1 ♀ (ZMAS); **South Ossetia**, Cey, along Ceydon river [not located], 16.vi.1981, S. Alekseev leg., 1 ♀ (ZMUM);

**Germany:** **Baden-Württemberg state:** Bad Wimpfen, Scriba, without date, K. Neumann leg., 1 ♂, 1 ♀ (SMFD); Baden (Historical State), 1902, Dr. Stolz leg., 1 ♀ (NHMW); same locality, 1903, Dr. Stolz leg., 1 ♂ (NHMW); Breisgau, 5.v.1973, without collector’s name, ♂ (SMNS); Giengen, 8.ix.1992, without collector’s name, coll. Piesbergen, 1 ♂ (SMNS); Herrlingen, 27.vi.1954, R. Schrepfer leg., coll. R. Schrepfer, 1 ♀ (SMNS); Ludwigsburg Dist., Gerlingen, 1.vi.1971, A. Greb leg., 1 ♀ (SMNS); Kaiserstuhl (Mts.), v.-vi.1949, without collector’s name, 1 ♂ (ZMHB); same locality, without date, Bischoff leg., 1 ♀ (ZMHB); Mosbach baths, v.1941, Schaaff leg., coll. Bosch, 1 ♂ (SMFD); Schwarzwald (Black Forest), v.1999, Stock leg., 1 ♀ (SMFD); same locality, without date, Schonfeldt leg., 2 ♀ (SMFD); River Danube, Ulm, 1955, G. Scheel leg., 1 ♀ (SMNS); Winterlingen, Rauhe Alb [not located], without date, Dr. Burkart leg., 1 ♂ (SMNS); same locality, Alb. Wurtt. [not located], without date, Dr. Burkart leg., 1 ♂ (SMNS); **Bavaria state:** Burghauser, without date, Mahler, Waka, Fleischer & Absolon leg., 1 ♂ (MZMB); Forchheim, 29.v.1955, Dr. Eeerlein leg., 1 ♂ (NHMW); Hackelsberg, 23.iv.1964, Weinten leg., 1 ♂ (SMFD); Höchberg, v.1978, without collector’s name, coll. Schwarzer, 1 ♀ (SMFD), Karlstadt, fir forest, 1.vi.1952, Eckerlein leg., 1 ♀ (NHMW); same locality, 6.v.1956, Eckerlein leg., 1 ♂ (NHMW); Rohrbach Dist., Neuburg an der Donau, without date, Karl Ruile leg., coll. Dr. Jhssen, 1 ♀ (ZMHB); Sugenheim, without date, W. Sattler leg., 1 ♀ (SMFD); Treuchtlingen, without date and collector’s name, general coll., 1 ♂ (SDEI); Wegscheid, 8.vii.1920, Dr. Priesner leg., 1 ♀ (NHMW); Weisendorf, 25.vi.1965, Marchied leg., 1 ♀ (ZMHB); Wechselberg, without date, S. Breuning leg., ex coll. S. Breuning, coll. P. v. d. Wiel, 1 ♀ (ZMAN); Würzburg, 6.vi.1955, Siegm Hardorfer leg., coll. Stegmann, 1 ♀ (SMNS); **Bavaria/ Hesse and Thuringia states border:** Rhön (Mts.), without date and collector’s name, 1 ♀ (SMTD); **Brandenburg state:** Senftenberg, without date and collector’s name, 1 ♀ (NHMW); **Hesse state:** Bickenbach, 1.vi.1916, without collector’s name, 1 ♂ (SMFD); Darmstadt, 1.v.1951, Hermann Vogt leg., Hessen collection, 1 ♀ (SMFD); same locality, 11.v.1952, Hermann Vogt leg., 1 ♂ (SMFD); Eberstadt, 1959, Rebmann leg., 1 ♂ (SMFD); Flörsheim am Main, 30.iv.1905, H. Bucking leg., 1 ♀ (SMFD); same locality, 10.iv.1914, Axel Krogh leg., 1 ♂ (SMFD); Frankfurt (forest), 12.v.1901, coll. C. le Dous, 1 ♂ (ZMUC); same locality, Frankfurt, Hyd. leg., 1 ♀ (ZSM); same locality, without date, D. J. Stiebel leg., 1 ♂, 1 ♀ (SMFD); same locality, without date, A. Weis leg., 1 ♂ (SMFD); same locality, without date and collector’s name, 1 ♂ (SMFD); Fulda, 1.vi.1957, P. S. Wagner leg., 1 ♀ (ZMAN); same locality, 6.iv.1983, J. Frisch leg., 1 ♀ (ZMHB); same locality, 30.iv.1984; J. Frisch leg., 1 ♂ (ZMHB); same locality, 14.v.1983, J. Frisch leg., 1 ♂ (ZMHB); Kühkopf (Rhein), 19.v.1951, Dr. Hermann Vogt leg., Hessen collection, 1 ♀ (SMFD); same locality, 9.vi.1951, Wagner leg., 1 ♂ (ZMAN); same locality, 8.v.1960, N. Schurmann leg., Hessen collection, 1 ♂ (SMFD); same locality, 18.v.1899, A. Weis leg., 1 ♂ (SMFD); Lorch am Rhein, 21.vi.1958, Schurmann leg., Hessen collection, 1 ♂ (SMFD); same locality, 28.iv.1961, Schurmann leg., Hessen collection, 1 ♀ (SMFD); same locality, 29.iv.1966, Liebegott leg., coll. D. Liebegott, 1 ♀ (SMFD); same locality, Nollig Castle, 9.v.1968, Nollig leg., 1 ♂ (SMNS); Oestrich-Winkel, without date and collector’s name, 1 spec. (ZMHB); Rhönnebirge-Römershag, 21.v. [year not provided], G. Ochs leg., 1 ♀ (SMFD); Sachsenberg, 23.v.1924, Dr. Feige leg., 1 ♀ (SMNS); Seligenstadt, without date, K. Neumann leg., 1 ♂, 1 ♀ (SMFD); **Lower Saxony state:** Hannover, without date, G. Reineck leg., coll. Bosch, 1 ♀ (SMFD); same locality, without date and collector’s name, coll. W. H. Muche, 1 ♀ (SMTD); Lilienthal- Kaiserstuhl, 24.iv.1937, Bischoff leg., 2 ♂ (ZMHB); Wendeburg, 2.vii.1920, K. Benner leg., coll. F. C. Drescher, 1 ♂ (ZMAN); **Mecklenburg-Western Pomerania state**, without date, Redeberg leg., 4 ♂ (ZMHB); **North Rhine-Westphalia state**, Düsseldorf, without date, K. Ermisch leg., 1 ♀ (ZMHB); **Rhineland-Palatinate state:** Ahrweiler Dist., Scriba, without date, K. Neumann leg., 1 ♀ (SMFD); Alzey, Dintheshein [not located], 15.iv.1973, Fell Rathmacher leg., 1 ♀ (SMNS); Asselheim, without date, O. Rebmann leg., 1 ♀ (SMFD); Bad Dürkheim, without date, Eppelsheim leg., coll. Eppelsheim, 1 ♀ (NHMW); Breisgau, 5.v.1973, without collector’s name, 1 ♂ (SMNS); Budenheim, 7.vi.1926, H. Muller leg., Hessen collection, 1 ♂ (SMFD); Frankenthal, 21.ix.1917, Dr. Feige leg., 1 ♀ (ZMHB); same locality, 16.v.1918, Dr. Feige leg., ♀ (ZMHB); same locality, 20.v.1924, Dr. Feige leg., 1 ♂ (ZMHB); Gau-Algesheim, 25.v.1925, H. Bucking leg., 1 ♀ (SMFD); same locality, 28.iv.1895, W. Sattler leg., 1 ♂ (SMFD); Mainz, 6.v.1984, Schonfeldt leg., 1 ♀ (SMFD); same locality, Mainz Sand Dunes (Botanical Reg.), 8.v.1960, Liebegott leg., coll. D. Liebegott, 1 ♂ (SMFD); same locality, 6.v.1984, Schonfeldt leg., 1 ♀ (SMFD); Mainz, Mombach, 12.v.1901, Stock leg., 1 ♀ (SMFD); same locality, 27.iv.1918, Karl Stock leg., 1 ♂ (SMFD); same locality, 20.iv.1961, without collector’s name, 1 ♀ (SMFD); Neustadt an der Weinstraße (formerly Neustadt an der Haardt), 4.vi.1931, Schaaff leg., 1 ♂ (SMFD), same locality, 6.vi.1931, Schaaff leg., 1 ♀ (SMFD); Ockenheim, 29.iv.1913, Karl Stock leg., 1 ♂ (SMFD); Rheinhessen or Rhenish Hesse [wine region], 1921, Buchka & Axel Krogh leg., 1 ♀ (SMFD); same locality, 24.iv.1898, without collector’s name, 1 ♂ (SMFD); Rhein-Pfalz-Kreis Dist., without date and collector’s name, 1 ♀ (NMPC); **Saarland state**, Weiselberg, 8.ix.1965, A. Kirch leg., 1 ♀ (SMFD); **Saxony state:** Dresden, 7.ix.1890, without collector’s name, coll. Prof. Noesske, 1 ♀ (SMTD); Nossen, without date and collector’s name, coll. Fuchs, 1 ♀ (SMTD); Seewiesen (Noth of Breitenbrunn), v.1977, R. Papperitz leg., coll. R. Papperitz, 1 ♂ (SMNS); Waldheim, without date and collector’s name, coll. Fuchs, 1 ♂ (SMTD); **Saxony-Anhalt state:** Eisleben, without date, M. Naumann leg., 1 ♀ (SMTD); same locality, Susser See lake, 1.vi.1938, Dr. Feige leg., 1 ♀ (ZMHB); Freyburg (toboggan), 2.ix.1956, K. Ermisch leg., 1 ♀ (ZMHB); Harz Dist., without date and collector’s name, general coll., 1 ♂ (SDEI); same locality, without date and collector’s name, coll. W. H. Muche, 1 ♂ (SMTD); Harz Dist., Thale, Kärrlingsberg [not located], 17.vii.1951, Dorn leg., 1 ♂ (ZMHB); same locality, 22.v.1952, Dorn leg., 1 ♀ (ZMHB); same locality, 4.vi.1953, Dorn leg., 1 ♂, 1 ♀ (ZMHB); same locality, 12.viii.1954, Dorn leg., 1 ♀ (ZMHB); same locality, 27.viii.1954, Dorn leg., 1 ♂ (ZMHB); Könnern, vii.1962, without collector’s name, 1 ♀ (ZMHB); Laucha an der Unstrut, without date and collector’s name, coll. C. Schenkling. general coll., 3 ♀ (SDEI); Naumburg, sledding area, 25.ix.1936, Maertens leg., 1 ♂ (SMTD); **Thuringia state:** without date and collector’s name, 1 ♀ (HNHM); Altenburg, Hundsheimer Berg [not located], 1.-3.vii.1941, Biscnott leg., 1 ♂ (ZMHB), Altenburg, Auenwald [not located], 29.v.1942, Biscnott leg., 1 ♂ (ZMHB); Arnstadt, 23.v.1920, W. Liebmann leg., general coll., 2 ♂ (SDEI); Bad Frankenhausen, viii.1950, Karl Ermisch leg., 1 ♂, 1 ♀ (ZMHB); same locality, viii.1951, Karl Ermisch leg., 1 ♂ (ZMHB); same locality, 23., 25.v.1953, Karl Ermisch leg., 1 ♂ (ZMHB); same locality, 21.v.1956, Karl Ermisch leg., 1 ♂ (ZMHB); Bleicherode, P. Eigen leg., coll. W. H. Muche, 1 ♂ (SMTD); Bretleben, 14.v.1905, A. Petry leg., 1 ♀ (ZMHB); Frankenhausen, without date, Moosbrugger leg., 1 ♂ (NHMW); Gotha, Veretpsgarten Galberg [not located], v.1907, coll. K. Hanel, 1 ♀ (SMTD); Jonastal [Jonas Valley], 4.vi.1954, Dorn leg., ♀ (ZMHB); Kyffhäuser Gebirge [Kyffhäuser hills], 1.ix.1953, 1 ♂ (ZMHB), same locality, vi.[19]54, without collector’s name, 1 ♀ (ZMHB); Kyffhäuser Gebirge [Kyffhäuser hills], Galgenberg, 18.iv.1959, without collector’s name, 1 ♀ (SMNS); Kyffhäuser Gebirge [Kyffhäuser hills], Kosakenberg, 21.ix.1958, Dorn leg., 2 ♂, 2 ♀ (ZMHB); Naumburg, 12.iv.1925, Dr. Maertens leg., 1 ♂ (ZMHB); same locality, 24.v.1925, Dr. Maertens leg., 1 ♀ (ZMHB); same locality; 2.vi.1925, Dr. Maertens leg., 1 ♂ (ZMHB); same locality, 25.vii.1925, Dr. Maertens leg., 1 ♂ (ZMHB); same locality, 5.vi.1927, Dr. Maertens leg., 1 ♂ (ZMHB); same locality, 29.iv.1928, Dr. Maertens leg., 1 ♂, 3 ♀ (ZMHB); same locality, 14.v.1929, Dr. Maertens leg., 1 ♀ (ZMHB); Naumburg, Totintal, 21.v.1903, Stockhausen leg., 1 ♀ (ZMHB); Naumburg, Großwilsdorf, 13.v.1951, Dorn leg., 1 ♀ (ZMHB); Naumburg, Roßbach, 13.v.1951, Dorn leg., 1 ♂ (ZMHB); Waldeck, Sachsenhausen, without date and collector’s name, coll. Rosenberg, 1 ♂ (ZMUC); Tautenburg, v.-vi.19[60], without collector’s name, 2 ♂ (SMTD); Weimarer Dist., Buchfart, 1895, G. Reineck leg., 1 ♂ (SMFD); same locality, 1897, Reineck leg., 1 ♀ (SMFD); Saihsengurge [not located], 12.v.1912, A. Petry leg., ♀ (ZMHB); Futher l. jega [not located], 25.v.1921, A. Petry leg., ♂ (ZMHB); Blaic Ze..berga [not located], 27.iv.1915, leg. A. Petry, ♂ (ZMHB); Frau [not located], 20.v.1905, leg. A. Petry, ♀ (ZMHB); Seit..burg [not located], 14.v.1911, leg. A. Petry, ♀ (ZMHB); **Upper Bavaria state**, Miesbach, without date, Tratzl leg., 2 ♀ (NMPC);

**Greece:** **Acarnania Reg.**, without date and collector’s name, 1 ♀ (NMPC); **Attica Reg.:** Athens, Glyfada, 29.iv.1973, coll. D. Liebegott, 1 ♂ (SMFD); Athens, Kaisariani, without date, Bartoň leg., 1 ♂ (NMPC); Athens, Nea Ionia, Pefkakia station, 2.vi.1980, Epping leg., 1 ♂ (SMNS); Athens, 1960, without collector’s name, coll. C. & O. Vogt, 1 ♀ (ZMAN); same locality, without date, Berinek leg., 1 ♀ (ZMHB); same locality, without date, Fankrati leg., 1 ♀ (NHMW); same locality, without date, Zebe leg., 1 ♀ (ZMUC); same locality, without date and collector’s name, coll. W. H. Muche, 1 ♂ (SMTD); same locality, without date and collector’s name, 1 ♀ (NMPC); (Cape) Sounion, 13.v.1964, Eckerlein leg., 2 ♂ (SMTD); Faliro, without date, Oertzen leg., 1 ♂ (ZMHB); Piraeus, without date, 2 ♂ (BMNH); Rafina, 15.iv.1960, W. Kühnelt leg., 1 ♀ (NHMW); Attica, without specific locality, without date and collector’s name, 3 ♂, 2 ♀ (NMPC); Attica, without specific locality, 1882, Oertzen leg., 1 ♂, 1 ♀ (NHMW); Attica, 1907, Leonis leg., 1 ♀ (NHMW); Attica, 14.iv.1922, without collector’s name, coll. W. Liebmann, general coll., 1 ♂, 4 ♀ (SDEI); Attica, 27.iv.1967, E. Witt leg., 1 ♂ (SMFD); Attica, 9.v.1869, without collector’s name, coll. Kraatz, general coll., 1 ♂ (SDEI); Attica, without date, Leonis leg., 1 ♂ (NMPC); Attica, without date and collector’s name, coll. W. H. Muche, 2 ♂, 1 ♀ (SMTD); Attica, without date and collector’s name, coll. Noesske, 3 ♂, 2 ♀ (SMTD); Attica, without date and collector’s name, coll. Reitter, 1 ♀ (HNHM); Attica, without date and collector’s name, coll. Thieme, 1 ♂, 1 ♀ (ZMHB); Attica, without date and collector’s name, 1 ♂ (HNHM); Attica, without date and collector’s name, 3 ♂, 1 ♀ (NHMW); Attica, without date and collector’s name, 1 spec. (NHMW); Attica, without date and collector’s name, 3 ♀ (NMPC); **Central Greece Reg.:** Euboea, Chalcis, 26.v. [without year], Holtz leg., 1 ♀ (ZMHB); Boeotia, Chalía, without date, Holtz leg., 1 ♂ (NMPC); Mount Parnassus, without date and collector’s name, coll. Hauser, 1 ♂ (NHMW); same locality, without date and collector’s name, coll. Thieme, 1 ♀ (ZMHB); same locality, without date and collector’s name, coll. W. H. Muche, 1 ♂ (SMTD); same locality, without date and collector’s name, 1 ♂ (HNHM); same locality, without date and collector’s name, 1 ♀ (NHMW); Phocis, Nafpaktos, 14.iv.1979, S. Vit leg., 1 ♂ (MHNG); **Central Macedonia Reg.:** Alistrati, 29.iv.1984, H. Schonmann leg., 1 ♂, 1 ♀ (NHMW); Amfipoli, 28.v.2007, Tomáš Sitek leg., 2 ♂, 1 ♀ (TSIC); Asprovalta, 17.vi.1976, Marggi leg., 1 ♀ (SMNS); same locality, 24.vi.1984, Epping leg., 1 ♂, 1 ♀ (SMNS); Edessa (West), 17.iv.1980, Krätschmer leg., 1 ♀ (SMNS); Evzoni, 10.v.1991, G. Guevare leg., 1 ♀ (JRUC); Chalkidiki Peninsula, Kassandra, viii.2002, Olejníček leg., ex coll. Olejníček, 4 ♂, 11 ♀ (NMPC); Katerini, 4.iv.1987, J. Frisch leg., 1 ♀ (ZMHB); Litochoro, without date, Bartoň leg., 2 ♂ (NMPC); Mount Olympus, without date, Bartoň leg., 1 ♀ (NMPC); Serres, Simvoli, 30.iv.1983, H. Schonmann leg., 1 ♂, 1 ♀ (NHMW); Serres, (3 km North), 41°07'4"N, 23°33'5"E, field, pasture-steppe, 250 m, 26.iv.2007, Jiří Hájek, 2 ♂, 1 ♀ (JRUC); same data, 1 ♂, 2 ♀ (NMPC); Stavros, 20-300 m, 1.-12.vi.1997, Jan Farkač leg., 1 ♀ (JRUC); Thessaloniki (Salonica), 13.v.1968, A. Senglet leg., 1 ♂, 1 ♀ (MHNG); Thessaloniki, 3 km South of Tagarades, 40°28'1"N, 23°00'8"E, olive orchard, field, 170 m, 27.iv.2007, Jiří Hájek leg., 1 ♂, 1 ♀ (JRUC); same data, 2 ♂ (NMPC); Vafiochorion, Castro, 4.iv.1982, Korell leg., 1 ♂ (SMNS); **Crete Island:** without specific locality, v.1904, without collector’s name, 1 ♀ (NHMW); same locality, without date, Holtz leg., 1 ♀ (ZMHB); same locality,without date, Paganetti leg., 1 ♂, 1 ♀ (HNHM); same locality, without date, Paganetti leg., 2 ♀ (NHMW); same locality, without date, Paganetti leg., 2 ♂, 4 ♀ (NMPC); same locality, without date, Paganetti leg., 4 ♂, 2 ♀ (ZMHB); same locality, without date, Paganetti leg., coll. J. Fleischer, 1 ♀ (NMPC); same locality, without date and collector’s name, coll. Kamberský, 1 ♂ (NMPC); same locality, without date and collector’s name, 1 ♂, 1 ♀ (BMNH); same locality, without date and collector, 1 ♀ (NHMW); same locality, without date and collector’s name, 1 ♀ (ZMHB); Amnisos, 20.x.1972, A. C. & W. N. Ellis leg., 2 ♂, 2 ♀ (ZMAN); Chania, ii.1906, Biro leg., 3 ♂, 3 ♀ (HNHM); same locality, iv.1906, Biro leg., 1 ♀ (HNHM); same locality, 5.iii.1925, A. Schulz leg., 1 ♂ (ZMHB); same locality, 31.iii.1925, A. Schulz leg., 1 ♀ (ZMHB); same locality, iv.1926, Holtz leg., 1 ♀ (ZMHB); same locality, 26.iv. (without year), Holtz leg., 1 ♂ (ZMHB); same locality, without date, Maltzan leg., 1 ♂, 1 ♀ (ZSM); same locality, without date and collector’s name, 1 ♂ (HNHM); Georgioupoli, 10.x.1925, A. Schulz leg., 1 ♀ (ZMHB); Heraklion, iv.1906, Biro leg., 3 ♂, 3 ♀ (HNHM); same locality, 18.vi.1925, A. Schulz leg., 1 ♂ (ZMHB); same locality, 14.v.1963, K. Kusdas leg., 1 ♂ (NHMW); same locality, 22.v.1963, K. Kusdas leg., 1 ♀ (NHMW); same locality, 26.v.1963, K. Kusdas leg., 1 ♀ (NHMW); same locality, 23.-27.iv.1986, Z. Meszaros leg., 1 ♀ (HNHM); same locality (6 km West), 10.iv.1975, J. A. W. Lucas leg., 1 ♂ (ZMAN); Chora Sfakion, 26.iii.1992, Motol leg., 2 ♂ (JRUC); Kalyves, 27.iii.1925, A. Schulz leg., 1 ♀ (ZMHB); Kandia, without date, Mařan, Štěpánek & Bartoň leg., 1 ♂, 2 ♀ (NMPC); Knossos, 1983, without collector’s name, coll. Stegmann, 1 ♀ (SMNS); same locality, iv.1988, without collector’s name, 1 ♂ (NHMW); same locality, without date, Mařan, Štěpánek & Bartoň leg., 1 ♂ (NMPC); Kritsa (North) between Dramia and Kalami, 25.-26.iii.1925, A. Schulz leg., 1 ♀ (ZMHB); Malia, 17.iv.1971, W. Wittmer leg., 1 ♂ (SMNS); same locality, v.1972, Messutat leg., 1 ♂ (SMNS); Meradhes (in the South-West), Kastelli Kissamu, 7.iii.1925, A. Schulz leg., 1 ♂, 1 ♀ (ZMHB); Mires, iii.1982, G. Sama leg., 1 ♀ (SMNS); Mitass, Epar. Kanurgion [not located], 16.v.1925, A. Schulz leg., 1 ♀ (ZMHB); Crete, 1884, Oertzen leg., 1 ♀ (NHMW), same locality, Viano [Viannos], without date, Oertzen leg., 1 ♀ (ZMHB); Phaistos, iii.1981, Schurmann leg., 1 ♀ (SMNS); Rethimnon, 14.iv.1980, Korell leg., 1 ♂, 2 ♀ (JRUC); same locality, Melambes (15 km South-East Spili), 35°08'N 24°39'E, 200 m, 7.v.1995, C. Lange & J. Ziegler leg., general coll., 1 ♂, 1 ♀ (SDEI); Rethymno, 8.-29.v.1979, R. Köstlin leg., coll. R. Köstlin, 1 ♀ (SMNS); Rethymno, Amari Valley, 100-300 m, 5.-11.iv.1991, P.M. Hammond leg., 1 ♂ (BMNH); Rethymno, Episkopi (8 km West of Perama), 35°22'N 24°37'E, 100 m, 28.iv.1995, C. Lange & J. Ziegler leg., general coll., 1 ♂, 1 ♀ (SDEI), Rethymno, 12.x.1925, A. Schulz leg., 1 ♂ (ZMHB); Yoryinpoli [not located], 16.v.2001, H. Schmalfuss leg., 1 ♀ (SMNS); **East Macedonia and Thrace Reg.:** Aristino valley (8 km North of Xanthi), Alexandroupoli, v.1979, Krätschmer leg., 2 ♂, 1 ♀ (SMNS); Alexandroupoli, v.1979, Krätschmer leg., 1 ♂ (SMNS); same locality, without date, Bartoň leg., 1 ♂ (NMPC); Drama, without date, Weirather leg., 1 ♂, 3 ♀ (MHNG); Evros (10 km North-West Didymoteicho), Quercus, cultural-land, 50 m, 22.iv.1994, Bense leg., 1 ♀ (SMNS); Galipsos, 1983, Krätschmer leg., 1 ♂ (SMNS); Chalkero (8 km East of Kavala), 40°58'03"N, 24°28'02"E, pasture, 70 m, 25.iv.2007, Jiří Hájek leg., 1 ♀ (JRUC); same locality, without date, Jiří Hájek leg., 1 ♀ (NMPC); Kavala, vi.1973, without collector, 1 ♂, 2 ♀, 1 spec. (MHNG); same locality, v.1979, O. Krätschmer leg., 1 ♀ (SMNS); same locality, 550 m, 3.v.1984, Krätschmer leg., 1 ♂ (SMNS); same locality, 17.vi.1993, M. Jäch leg., 1 ♂ (NHMW); same locality, Pangeo sopra, Akrovounion, 1500 m, 7.vii.1983, 1 ♂ (MHNG); Komotini, 22.iv.1965, 1 ♂ (SMNS); Thasos Island, 11.vi.1967, Epping leg., 1 ♂ (SMNS); Thasos Island, Crisso Akroyiali [probably App. Akrogiali] near Potamia, 9.viii.1972, M. I. Russell leg., 1 ♂ (BMNH); Thasos Island, Panagia, 27.v.1999, H. Schmalfuss leg., 1 ♂ (SMNS); Xanthi, v.1979, Krätschmer leg., 1 ♂ (SMNS); same locality, without date, Bartoň leg., 1 ♂, 1 spec. (NMPC); same locality, valley, 16.iv.1982, Krätschmer leg., 1 ♂ (SMNS); **Epirus Reg.:** Arta, 5.iv.1973, Krätschmer leg., 1 ♂, 1 ♀ (SMNS); Ioannina, vii.1968, W. Kühnelt leg., 1 ♀ (NHMW); Megalo Peristeri, 23.v.1969, without collector’s name, 1 ♀ (MHNG); Pindus (Mt.), Metsovo, 20.iv.1973, Krätschmer leg., 2 ♂ (SMNS); Thesprotia, Igoumenitsa, 5.iv.1973, Krätschmer leg., 1 ♂ (SMNS); same locality, Karteri, 12.vi.2008, K. Orszulik leg., 3 ♂, 3 ♀ (KORC); **Eastern Evrytania / Western Phthiotis Reg. border:** Tymfristos (Mt.), without date and collector’s name, 1 ♀ (NMPC); **Ionian Islands:** Cephalonia Island: 1905, O. Leonhard leg., general coll., 2 ♂ (SDEI); Argostoli, 1908, M. Hilf leg., ex coll. O. Leonhard, coll. Noesske, 1 ♂ (SMTD); same locality, 2.-8.v.1929, Beier leg., 1 ♀ (NHMW); same locality, 12.-18.x.1972, G. Benick leg., 1 ♂ (ZMHB); same locality, 10.-15.v.1980, W. H. Gravestein leg., 1 ♂ (ZMAN); Charakti, 1908, M. Hilf leg., coll. O. Leonhard, general coll., 1 ♂ (SDEI); Corfu island, 1903, Paganetti leg., coll. W. H. Muche, 1 ♀ (SMTD); same locality, 9.v.1913, A. Kramer leg., 1 ♀ (ZMHB); same locality, without date, Paganetti leg., coll. J. Fleischer, 1 ♀ (NMPC); same locality, without date and collector’s name, 1♂, 2 ♀ (SMTD); Lefkada island, 8.-30.iv.1929, Beier leg., 1 ♂, 1 ♀ (NHMW); same locality, Kallighoni, 26.iii.1971, I. Löbl leg., 1 ♀ (MHNG); Zakynthos Island, 22.iii.1936, Eigelt leg., 1 ♀ (NHMW); same locality, Kalamaki, 1909, M. Hilf leg., coll. O. Leonhard, general coll., 3 ♀ (SDEI); Ionian Islands without specific locality, without date, Gabriel leg., coll. Bosch, 1 ♂ (SMFD); **North Aegean Reg.:** Lemnos Island (2 km South of Kalliopi), 39°54'03"N, 25°20'06"E, marsh-saline: sand-dunes, 5 m, Jiří Hájek leg., 1 ♂ (NMPC); Samos Island, 26.v.-6.vi.1987, Bilek, Kritscher leg., 1 ♀ (NHMW); **Peloponnese Reg.:** Achaia county, Panahaiko (Mts.), 29.iv.1998, Behne leg., general coll., 2 ♀ (SDEI); Arkadia, Megalopolis, 12.iv.1973, Krätschmer leg., 1 ♀ (SMNS); Corinth, without date and collector’s name, 2 ♂, 1 ♀ (NHMW); Dymi (Kato Achaia), Patras, v.1972, Messutat leg., 1 ♀ (SMNS); Epidaurus, 20.v.1937, F. Warner leg., 1 ♀ (NHMW); Galatas, 7.-28.vi.1976, Köstlin leg., 1 ♂ (SMNS); Laconia (South West of Skala), 30.ix.1962, without collector’s name, 1 ♀ (ZMAN); Loutraki, 50 m, v.1974, Weisz leg., 1 ♀ (NHMW); Taygetus (Mts.), without date, Brenske leg., coll. Schwarzer, 1 ♂ (SMFD); same locality, without date, Dr. Kinper leg., coll. Hauser, 1 ♂ (NHMW); Taygetus, Kalamata, 4.ix.1977, L. Toth leg., 1 ♀ (HNHM); Taygetus, without date and collector’s name, 1 ♀ (NHMW); Tripotama, Erymanthos valley, 21.iv.1980, Krätschmer leg., 1 ♂ (SMNS); **South Aegean Reg.:** Naxos Island, without date, Dr. Kinper leg., coll. Hauser, 2 ♂, 1 spec. (NHMW); Rhodes Island, without date and collector’s name, 1 ♀ (NMPC); same locality, without date and collector’s name, coll. Reitter, 1 ♀ (NMPC); **Thessaly Reg.:** Karya, iv.1980, Krätschmer leg., 1 ♂ (SMNS); Larissa (7 km South of Elasson), v.1980, Krätschmer leg., 1 ♀ (SMNS); Mount Ossa, Oros, 1000 m, 18.iv.1980; Krätschmer leg., 1 ♂ (SMNS); Sulopulon [not located], 26.iii.1978, S. Vit leg., 1 ♀ (MHNG); Skopelos Island, vi.1985, Bilek & Kritscher leg., 1 ♀ (NHMW); Sporades, Skiathos Island, 17.iii.1976, Liebegott leg., coll. D. Liebegott, 1 ♂ (SMFD); same locality, 20.iv.1976, Liebegott leg., 1 ♀ (SMNS); same locality, 2.vi.1979; Liebegott leg., 1 ♂ (SMFD); same locality, 15.ix.1991, Liebegott leg., 1 ♀ (SMFD); Tyrnavos, 18.iv.1973, Krätschmer leg., 1 ♀ (SMNS); Volos, without date, Stussiner leg., coll. Fry, 1 ♀ (BMNH); same locality, without date, Stussiner leg., 1 ♂, 1 ♀ (NMPC); **West Greece Reg.:** Achaea, Kalogria, 20.-21.v.2004, Angelini leg., 2 ♂, 1 ♀ (JRUC); Kalavryta, without date, Bartoň leg., 1 ♂ (NMPC); Olympia, without date and collector, 1 ♀ (NHMW); same locality, without date and collector, 1 ♀ (NMPC); **Greece without specific locality:** 7.vii. (without year), without collector’s name, 1 ♂ (MHNG); vii.1907, without collector’s name, 1 ♂ (MHNG); without date, L. Salvador leg., 1 ♂ (NMPC); without date, Roth & Dohrn leg., 1 ♀ (ZSM); without date, Walff leg., 1 ♂ (ZMHB); without date and collector’s name, coll. Merlin, 1 ♀ (BMNH); without date and collector’s name, coll. Thieme, 1 ♀ (ZMHB); without date and collector’s name, 1 ♀ (BMNH); without date and collector’s name, 1 ♂ (MNCN); without date and collector’s name, 2 ♀ (NMPC); without date and collector’s name, 1 ♀ (ZMHB);

**Hungary:** **Bács-Kiskun County:** Kunpeszér, 22.iv.1979, Adam leg., 1 ♂ (HNHM); Szalkszentmarton, 14.vii.1995, O. Merkl leg., 1 ♀ (HNHM); Ficser, Puszta (grassland biome), 19.vi.1995, J. Probst leg., 2 ♂ (JRUC); Kalocsa, without date, Speiser leg., coll. F. Speiser, 2 ♀ (HNHM); Karapáncsa, without date and collector’s name, coll. Peregi, 1 ♂, 1 ♀ (HNHM); Kiskunfélegyháza, without date, Motzar leg., 1 ♂ (HNHM); same locality, without date and collector’s name, 1 ♀ (HNHM); Kiskunság National Park, Fülöpháza, 19.v.1982, K. Kiss leg., 1 ♂ (HNHM); Kiskunság National Park, Lakitelek, Tóserdő, 21.ii.1979, Migaly Adam leg., 1 ♂ (HNHM); Kiskunság, Dabas, 12.-17.vi.1979, Adam & Hamori leg., 1 ♀ (HNHM); Kiskunság, Tabdi, 25.x.1977, Adam leg., 1 ♂ (HNHM); Lake Vadkert, 5.v.1936, Erdos leg., coll. J. Erdos, 1 ♂ (HNHM); Tompa, 11.vii.1956, Z. Kaszab leg., 1 ♂ (HNHM); **Baranya County:** Mecsek (Mts.), Zobakpuszta, Hidasi-völgy, without date, Z. Kaszab leg., 1 ♂ (HNHM); Nagyharsany, Harsány, 23.vi.1954, Kaszab & Szekessy leg., 1 ♀ (HNHM); Pécs, 1906, Bokor leg., 1 ♂ (HNHM); same locality, 1906, without collector’s name, ex coll. Bokor, 1 ♀ (HNHM); same locality, 1907, without collector’s name, ex coll. Bokor, 1 ♂ (HNHM); Villány, 18.viii.1954, Gy. Tópál leg., 2 ♀ (HNHM); Zamárdi, 17.viii.1972, Fzong leg., 1 ♂ (HNHM); **Borsod-Abaúj-Zemplén County**, Aggtelek, 26.v.1972, G. Szocs leg., 1 ♂ (HNHM); **Csongrád County**, Algyő, 27.iv. (without year), without collector’s name, 1 spec. (HNHM); Szeged, without date, Victor Stiller leg., coll. Stiller, 2 ♂ (HNHM); **Fejér County:** Besnyő, without date and collector’s name, 1 ♂ (NHMW); Csókakő, without date, Lichtenckert leg., 1 ♀ (HNHM); Ercsi, without date, Victor Stiller leg., coll. Stiller, 1 ♀ (HNHM); Martonvásár, Homokgödör, 27.iv.1955, Z. Kaszab leg., 1 ♂ (HNHM); Mezőfalva, without date and collector’s name, coll. R. Streda, 1 ♀ (HNHM); Székesfehérvár, 17.vi.1904, without collector’s name, 1 ♂ (HNHM); same locality, 1920, without collector’s name, coll. W. H. Muche, 2 ♂ (SMTD); same locality, without date, Lichtenckert leg., 1 ♂ (HNHM); **Győr-Moson-Sopron County:** Győr, 14.iv.1936, Révy leg., coll. D. Révy, 1 ♀ (HNHM); same locality, without date, E. Bokor leg., ex coll. E. Bokor, 1 ♂, 1 ♀ (HNHM); same locality, without date, E. Bokor leg., 1 ♂ (HNHM); Győr, pasture, 15.ix.1975, J. Strejček leg., 1 ♀ (JRUC); Dunakiliti, 29.vi.-19.viii.1996, O. Merkl leg., 1 spec. (HNHM); Mosonmagyaróvár, 27.iv.1944, Revy leg., coll. D. Révy, 1 ♂ (HNHM); Lake Neusiedl (Fertő-tó), without date and collector’s name, coll. R. Streda, 2 ♀ (HNHM); Sopron, viii.1918, without collector’s name, coll. R. Streda, 1 ♀ (HNHM); same locality, without date and collector’s name, coll. R. Streda, 1 ♀ (HNHM); **Hajdú-Bihar County:** Debrecen, without date and collector’s name, coll. D. Kanabé, 1 ♀ (HNHM); **Heves County:** Eger, 16.iv.1952, N. Vamos leg., 1 ♂ (HNHM); same locality, viii.1984, without collector’s name, 1 ♂ (ZMHB); **Komárom-Esztergom County:** Esztergom, 13.v.1916, E. Dudich leg., 1 ♀ (HNHM); same locality, 1919, without collector’s name, ex coll. Bokor, 1 ♂ (HNHM); same locality, 9.v.1925, Veghelyi leg., 1 ♂ (HNHM); same locality, 16.vi.1925, Veghelyi leg., 1 ♀ (HNHM); same locality, without date, Bokor leg., ex coll. Bokor, 1 ♀ (HNHM); same locality, without date and collector’s name, ex coll. Bokor, 1 ♀ (HNHM); **Nógrád County:** 10.-16.viii.1953, Gy. Tópál leg., 1 ♂ (HNHM); Legénd, without date, Tunkl leg., 1 ♀ (HNHM); **Pest County:** Budapest 1879, J. Langert leg., 1 ♀ (HNHM); same locality, 1883, Pavel leg., 1 ♀ (HNHM); same locality, 1931, Z. Kaszab leg., 1 ♂ (HNHM); same locality, iv.1935, Hory leg., 1 ♀ (HNHM); same locality, v.1954, without collector’s name, coll. Dr. Lenci, 1 ♂ (HNHM); same locality, without date, Csillebeh leg., 1 ♀ (HNHM); same locality, without date, E. Dudich leg., 1 ♂ (HNHM); same locality, without date, Kuthy leg., 1 ♂ (HNHM); same locality, without date and collector’s name, coll. H. Diener, 2 ♀, 1 ♂ (HNHM); same locality, without date and collector’s name, 5 ♂, 3 ♀ (NHMW); Budapest, Albertfalva, without date and collector’s name, coll. H. Diener, 1 ♂, 1 ♀ (HNHM); Budapest, Budafok, without date and collector’s name, ex. coll. Guranyi, 1 ♂ (HNHM); Budapest, Cinkota, Naplas-to, 22.iv.1992, without collector’s name, 1 ♀ (HNHM); same locality, 1.vii.1992, L. Nadai leg., coll. E. Csiki, 1 ♂ (HNHM); Budapest, Csepel, 31.vii.1994, O. Merkl leg., 1 ♀ (HNHM); Budapest, Káposztásmegyer, 100 m, 22.iv.1984, O. Merkl leg., 1 ♀ (HNHM); same locality, 25.v.1986, O. Merkl leg., 1 ♀ (HNHM); same locality, 12.v.-30.vi.1996, O. Merkl leg., 1 ♂ (HNHM); Budapest, Lágymányosi Bridge, 24.viii.1953, Révy leg., coll. D. Révy, 1 ♂ (HNHM); same locality, without date and collector’s name, coll. H. Diener, 1 ♀ (HNHM); Budapest, Rákoscsaba, 20.v.1966, L. Haraszthy leg., 1 ♂ (HNHM); same locality, 7.viii.1978, I. Kismarjai leg., 1 ♂ (HNHM); Budapest, Sashegy, without date and collector’s name, coll. H. Diener, 1 ♂ (HNHM); Budapest, Törökvész, 27.viii.1940, Csiki leg., coll. E. Csiki, 1 ♂ (HNHM); Budapest, Újpest, without date and collector’s name, coll. Noesske, 1 ♂ (SMTD); Budapest, Varosi erdo, 1.v.1966, L. Haraszthy leg., 1 ♂ (HNHM); Budaörs, 6.vii.1988, Korsos & Szel leg., 1 ♀ (HNHM); Érd, without date, Csiki leg., coll. E. Csiki, 1 ♀ (HNHM); same locality, without date and collector’s name, coll. E. Csiki, 1 ♂ (HNHM); Felsogod, 12.vi.1916, R. Streda leg., coll. R. Streda, 1 ♂ (HNHM); Gödöllő, 20.v.1896, without collector’s name, coll. E. Csiki, 1 ♀ (HNHM); Haraszt, 27.iv.1926, without collector’s name, 1 ♂ (HNHM); Leányfalu, without date and collector’s name, coll. R. Streda, 1 ♂ (HNHM); Lórév, 2.iv.2006, O. Merkl leg., 1 ♂ (HNHM); Nagykovácsi, Nagyszenas, without date, Kaszab leg., 1 ♀ (HNHM); Ócsa, 1953, Z. Kaszab leg., 1 ♀ (HNHM); Ócsa, Oreg turjan [not located], 24.iv.1952, Kakassne leg., 2 ♀ (HNHM); Szigetbecse, 100 m, 27.v.1987, O. Merkl leg., 1 ♀ (HNHM); Solymár, 26.iv.1953, Kovacsne leg., 1 ♀ (HNHM); same locality, 26.vi.1982, Racz leg., 1 ♂ (HNHM);Torokugrato hill, Budaörs, 1.vii.2005, Gyözö Szel leg., 1 ♂ (HNHM); Visegrád (castle town), 1905, Penther leg., 1 ♂ (NHMW); **Somogy County:** Kaposvár, 15.-16.iv.1960, M. Nattan leg., coll. M. Nattan, 1 ♂, 1 ♀ (HNHM); same locality, 29.iv.1961, M. Nattan leg., coll. M. Nattan, 1 ♀ (HNHM); same locality, 30.iv.1962, M. Nattan leg., 1 ♂ (HNHM); **Tolna County:** Simontornya, 1910, without collector, ex coll. Bokor, 1 ♂ (HNHM); same locality, iv.1911, without collector’s name, coll. Pillich, 1 ♀ (HNHM); same locality, 26.iv.1913, without collector’s name, coll. Pillich, 1 ♂ (HNHM); same locality, 27.iv.1927, without collector’s name, coll. Pillich, 1 ♂ (HNHM); Udvari, Alsopelpuszta, 22.v.1996, O. Merkl leg., 1 ♀ (HNHM); **Torontal County**, Újszeged, 22.iv.1932, Erdos leg., coll. J. Erdos, 1 ♂ (HNHM); **Veszprém County:** Balatonederics, without date and collector’s name, 1 ♂, 1 ♀ (HNHM); Berhida, iv.1954, Lenci leg., 1 ♀ (HNHM); same locality, vii.1954, without collector’s name, coll. Lenci, 1 ♀ (HNHM); same locality, without date, Szekessy leg., 1 ♂ (HNHM);Pápa, without date, E. Horvath leg., 1 ♂ (BMNH); Pilis Mts., Köhegy, 24.iii.1966, L. Haraszthy leg., 1 ♂ (HNHM); Tihany, 12.iv.1934, Mihalyi leg., 1 ♀ (HNHM); same locality, v.1941, Kaszab & Szekessy leg., 2 ♂, 1 ♀ (HNHM); Zirc, without date and collector’s name, 1 spec. (HNHM); **Zala County:** Keszthely, 1869, Dyeney leg., 1 ♂ (HNHM); Nova, without date and collector’s name, coll. R. Streda, 3 ♂, 1 ♀ (HNHM); **Hungary without specific locality:** 12.ix.1914, without collector’s name, 1 ♀, 1 spec. (ZMUC); without date and collector’s name, coll. E. Frivaldszky, 1 ♀ (HNHM); without date and collector, coll. Markel, 1 ♂ (SMTD); without date and collector’s name, coll. Noesske, 1 ♀ (SMTD); without date and collector’s name, 1 ♀ (BMNH); without date and collector’s name, 1 ♀ (ZMUC); without date and collector’s name, 1 ♀ (ZSM);

**Italy:** **Abruzzo Reg.:** Avezzano, vi.1899, B. Schwarzer leg., 1 ♂ (SMFD); L’Aquila prov., Cerchio, 1908, D’Amore leg., 3 ♂, 1 ♀ (NHMW); Monte Greco, without date, Paganetti leg., 1 ♀ (NHMW); same locality, without date, Paganetti leg., coll. Franklin Muller, general coll., 3 ♀ (SDEI); same locality, without date, Paganetti leg., coll. Rebmann, 1 ♀ (SMFD); without specific locality, without date, Paganetti leg., 1 ♀ (JRUC); **Apulia Reg.:** Brindisi, 3.iv.1973, Krätschmer leg., 2 ♂, 1 ♀ (SMNS); same locality, iv. (without year), Muche leg., coll. W. H. Muche, 1 ♂, 1 ♀ (SMTD); Castellaneta, 20.iv.1979, Duschurmann leg., 1 ♀ (NHMW); Foce di Capoiale, 1.v.1977, F. Angelini leg., coll. R. Kostlin, 1 ♀ (SMNS); Gargano, 7.v.1995, Mattinaio leg., 1 ♀ (JRUC); same locality, without date, L. S. Giovanni leg., 2 ♂, 3 ♀ (NHMW); Gargano, Mattinata, 5.v.1997, J. Vilimova leg., 1 ♀ (JRUC); Monte Gargano, 24.iv.1979, Schurmann leg., 1 ♂ (NHMW); same locality, 1907, M. Hilf leg., ex coll. O. Leonhard, coll. Noesske, 1 ♀ (SMTD); same locality, 1907, M. Hilf leg., coll. O. Leonhard, general coll., 1 ♂, 3 ♀ (SDEI); same locality, Peschici, 23.iii.1975, M. Niehuis leg., 2 ♂ (SMNS); Ginosa, Salinelle (are three fields of mud volcanoes), 17.xii.1978, F. Angelini leg., coll. R. Kostlin, 1 ♀ (SMNS); Grottaglie, iv.1974, 1979, Dr. Schurmann leg., 1 ♂, 1 ♀ (NHMW); same locality, without date, Paganetti leg., general coll., 1 ♀ (SDEI); same locality, without date, Paganetti leg., 1 ♀ (HNHM); same locality, without date, Paganetti leg., 1 ♂ (NHMW); same locality, without date, Paganetti leg., 1 ♀ (SMFD); same locality, without date, Paganetti leg. 1 ♂ (SMNS); same locality, without date and collector’s name, coll. Dr. J. Fodor, 1 ♀ (HNHM); Lecce prov., 25.-31.v.1930, Dannenberg leg., 1 ♀ (ZMHB); Lecce prov., Spongano, 21.vi.1930, Dannenberg leg., 1 ♀ (ZMHB); same locality, 16.-31.iii.1933, Loudon leg., 3 ♂, 2 ♀ (ZMHB); same locality, 5.-10.iv.1933, Loudon leg., 1 ♂ (ZMHB); same locality, 27.iv.1933, Loudon leg., 1 ♂, 2 ♀ (ZMHB); Manfredonia, without date, Hermann Vogt leg., Hessen collection, 1 ♀ (SMFD); same locality, without date, J. Fleischer leg., 1 ♀ (NMPC); Monte Sant’Angelo (Souther slope of Monte Gargano), without date, Stolz leg., 2 ♂ (NHMW); same locality, without date and collector’s name, 1 ♂ (NHMW); Taranto, iii.1919, without collector’s name, 2 ♂ (BMNH); **Calabria Reg.:** Sant’Eufemia d’Aspromonte, 1905, O. Leonhard leg, general coll., 1 ♀, 1 spec. (SDEI); Serra San Bruno, vi.1875, without collector’s name, 1 ♀ (MHNG); same locality, without date and collector’s name, coll. K. Mazura, 2 ♂ (MZMB); **Campania Reg.:** Naples, without date, B. Schwarzer leg., 1 ♀ (SMFD); same locality, without date, Muche leg., coll. W. H. Muche, 1 ♂ (SMTD); same locality, without date, Zimmermann leg., 3 ♂ (NHMW); same locality, without date and collector, coll. R. Streda, 2 ♀ (HNHM); Pioppi, 16.vi.1965, W. Liebmann leg., coll. E. Ulbrich, 1 ♀ (SMNS); Pompeii (in Napoli prov.); 15.iv.2003, P. Kment & M. Horsák leg., 1 ♂, 1 ♀ (JRUC); San Giorgio, without date, Sterba leg., 2 ♂, 1 ♀ (NMPC); Sturno, 1891, Zara Vecch leg., 1 ♂ (NHMW); **Emilia-Romagna and Tuscany Reg.**, Apennine (Mts.), Passo Dei Mandrioli, 6.vi.1963, R. H. Lechner leg., 1 ♂ (NHMW); **Emilia-Romagna Reg.:** Cattolica, iv.1952, H. Schmiett leg., 1 ♀ (SMFD); same locality, x.1962, Schirners leg., 1 ♂ (SMFD); Cesenatico, 29.iv.-8.v.1991, P. Průdek leg., 1 ♂, 2 ♀ (JRUC); Ravenna, without date, I. Kovář leg., 1 ♂, 1 ♀ (NMPC); Riccione, vi.1958, K. Ulbrich leg., coll. E. Ulbrich, 1 ♂ (SMNS); same locality, vii.1970, without collector’s name, coll. S. Kiener, 1 ♀ (MHNG); same locality, without date, Hájek leg., 7 ♂, 4 ♀ (NMPC); same locality, without date, Hájek leg., 1 ♂ (NMPC); Serramazzoni, 22.vii.1963, Lamholdt leg., 1 ♂ (ZMUC); **Friuli-Venezia Giulia Reg.:** Cervignano del Friuli, 20.vi.1910, Pinker leg., 1 ♀ (NHMW); Gorizia, viii.1908, Krekich leg., 1 ♂ (NHMW); Görz (Gorzia), without date, Gabriele leg., 4 ♀ (NHMW); Latisana, without date, Weiser leg., 1 ♂ (NMPC); Martiguacco, 15.iv.1963, without collector’s name, 1 ♂ (MHNG); same locality, 3.vii.1963, without collector’s name, 1 ♂ (MHNG); Monfalcone, 16.vii.1910, Pinker leg., 1 ♂ (NHMW); Noghera, 17.iv.1904, without collector’s name, 1 ♀ (NHMW); Tagliamento river, Latisana, without date, I. B. Weises leg., 1 ♂, 1 ♀ (NMPC); Trieste, 2.v.1984, H. Noack leg., 1 ♀ (SMFD); same locality, without date, Dr. Will leg., 1 ♀ (ZMHB); same locality, without date, Schiodte leg., 1 ♀ (ZMUC); same locality, without date, Ullrich leg., 1 ♀ (ZMUC); same locality, without date and collector’s name, coll. Bosch, 1 ♀ (SMFD); same locality, withou date and collector’s name, coll. Schneider-Kelch., general coll., 1 ♀ (SDEI); same locality, without date and collector’s name, coll. Krekich, 1 ♀ (NHMW); same locality, without date and collector’s name, 1 ♀ (NHMW); same locality, without date and collector’s name, 1 ♂ (NMPC); same locality, without date and collector’s name, 1 ♂ (SMTD); same locality, without date and collector’s name, 2 ♂, 3 ♀ (ZMHB); Udine prov., Martignacco, 15.iv.1963, without collector’s name, 1 ♂, 1 ♀ (MHNG); same locality, vi.1964, without collector’s name, 1 ♂, 1 ♀ (MHNG); same locality, 20.viii.1966, Cassutti-Morandini leg., 1 ♀ (MHNG); **Lazio Reg.:** Ausonia, 7.v.1965, without collector, coll. J. Erdos, 1 ♀ (HNHM); Latina prov., Parco Nazionale del Circeo, Sabaudia (environs of brackish laquna), 9.iv.2003, Horsák & Kment leg., 1 ♂ (JRUC); Rome, v.1896, without collector’s name, coll. W. H. Muche, 1 ♀ (SMTD); same locality, iv.1972, without collector’s name, coll. S. Kiener, 1 ♂ (MHNG); same locality, without date, Brenske & Schonfeldt leg., 1 ♀ (SMFD); same locality, without date, Dolm leg., 1 ♂ (NMPC); same locality, without date, O. Leonhard leg., general coll., 1 ♀ (SDEIC); same locality, without date, P. de Borre leg., 1 ♂ (MHNG); same locality, without date, Schiodte leg., 1 ♂ (ZMUC); same locality, without date, Šulc leg., 1 ♂ (NMPC); same locality, without date and collector’s name, coll. D. v. d. Hoop, 1 ♂ (ZMAN); same locality, without date and collector’s name, coll. Heyden, general coll., 1 ♂, 2 ♀ (SDEI); same locality, without date and collector’s name, coll. Schneider-Kelch., general coll., 1 ♀ (SDEI); same locality, without date and collector’s name, 1 ♂ (HNHM); same locality, without date and collector’s name, 2 ♂ (NHMW); same locality, without date and collector’s name, 2 ♂ (NMPC); same locality, without date and collector’s name, 1 ♀ (ZSM); Rome, Ciampino, 11.ix.1984, Miloš Fassati leg., 1 ♂ (JRUC); Rome, Ostia, without date, Privora leg., 1 ♂ (NMPC); Rome, Palatine Hill, without date, W. Sattler leg., 1 ♂ (SMFD); Rome, Tusculum, without date and collector’s name, 1 ♀ (NMPC); Tivoli (North-East Rome), without date and collector’s name, 1 ♀ (NMPC); without specific locality, without date, Bellagra leg., 1 ♂ (MHNG); **Liguria Reg.:** Finale Ligure, 5.v.1964, without collector’s name, 1 ♂ (MHNG); Genoa, without date, Natterer leg., 1 ♀ (NHMW); same locality, without date, Natterer leg., 1 ♀ (SMNS); Varazze, 1964, without collector’s name, coll. S. Kiener, 1 ♀ (MHNG); **Lombardy Reg.:** Grigna (Mts.), without date and collector’s name, 1 ♂ (ZSM); Mantua, Guidizzolo, ix.2002, P. M. Giachino leg., 1 ♀ (SMNS); Milan, without date, L. Franzel leg., coll. W. H. Muche, 1 ♂, 1 ♀ (SMTD); **Lombardy Reg. / Veneto Reg. and Trentino Alto-Adige Reg. border:** Lake Garda, viii.-ix.1912, without collector’s name, coll. Dr. Jhssen, 1 ♂ (ZMHB); same locality, without date, J. Fleischer leg., 1 ♀ (NMPC); same locality, without date, Mlynář leg., 1 ♂ (NMPC); **Magna Graecia Reg.**, Paestum, iv.1883, without collector’s name, 1 ♂ (ZMHB); **Marche Reg.:** Albacina, 24.iv.1960, J. Th. Skovgaard leg., 1 ♂ (ZMUC); Fano, Pesaro, without date, I. Kovář leg., 1 ♂ (NMPC); Macerata, v.1955, S. Battoni leg., 1 ♀ (MHNG); same locality, Gualdo, 4.vi.1979, Cola & Freude leg., coll. R. Kostlin, 1 ♀ (SMNS); Martinsicuro, 1970, Liebegott leg., coll. D. Liebegott, 1 ♂ (SMFD); Pontile, vi.1936, Cerruti leg., 1 ♂ (MHNG); **Molise Reg.:** Maiella (Mts.), 22.vi.1976, M. Curti leg., 5 ♂, 1 ♀ (MHNG); Ovada, without date and collector’s name, 1 ♀ (MHNG); Teramo prov., Prati di Tivo, 22.vi.1976, M. Curti leg., 1 ♀ (MHNG); **Piedmont Reg.:** Bussoleno, without date, Della Beffa leg., 1 ♀ (JRUC); Carcare, vi.1910, Luigi Bigliani leg., 1 ♀ (ZMHB); **Sardinia Island:** San Basilio, without date, Paganetti leg., coll. O. Leonhard, general coll., 3 ♂, 1 ♀ (SDEI); same locality, without date, Paganetti leg., coll. Franklin Muller, general coll., 2 ♂, 2 ♀ (SDEI); same locality, without date, Paganetti leg., general coll., 3 ♀ (SDEI); same locality, without date, Paganetti leg., 1 ♂, 1 ♀ (NHMW); same locality, without date, Paganetti leg., coll. J. Fodor, 1 ♂ (HNHM); same locality, without date, Paganetti leg., 2 ♀ (NMPC); **Sicily Island:** Messina, 11.x.1942, without collector, 1 ♂ (SMNS); Piano Zucchi, 16.iv.1979, Schurmann leg., 2 ♂, 1 ♀ (NHMW); Ragusa, 1808, Mann leg., 1 ♀ (NHMW); same locality, without date and collector’s name, coll. W. H. Muche, 1 ♂ (SMTD); same locality, without date and collector’s name, coll. K. Hanel, 1 ♀ (SMTD); same locality, without date and collector’s name, 1 ♂, 1 ♀ (NHMW); Sicily Island without specific locality: without date, Zeller leg., 1 ♀ (SDEI); without date, P. de Borre leg., 1 ♀ (MHNG); without date and collector’s name, 1 ♀ (MHNG); **Trentino-Alto Adige/Südtirol Reg.:** Bolzano, 1867, Mann leg., 1 ♀ (NHMW); Brixen, without date, Moosbrugger leg., 1 ♂ (NHMW); Eisacktal (South of Tyrol), Ritten, 6.vii.1983, J. Frisch leg., 1 ♂ (ZMHB); Nago-Torbole, without date and collector’s name, coll. Herb-Schmidt, 1 ♂, 1 ♀ (SMTD); Rovereto, without date, Troll leg., 1 ♂, 3 ♀ (NHMW); same locality, without date and collector’s name, 1 ♀ (NMPC); South Tyrol, 10.vi.1965, G. Scheel leg., 1 ♀ (SMNS); same locality, without date, Sarcatal & Schaaff leg., coll. Bosch, 1 ♂ (SMFD); Tirol, without date, Jureček leg., 1 ♂ (NMPC); same locality, without date and collector’s name, coll. C. Schenkling, general coll., 2 ♀ (SDEI); Trentino prov., without date, J. Waka leg., coll. Burghauser, Mahler, Waka, Fleischer & Absolon, 1 ♂, 1 ♀ (MZMB); Trentino, without date, Sarcatal & Schaaff leg., coll. Bosch, 1 ♀ (SMFD); **Trentino-Alto Adige/Südtirol Reg./ Veneto Reg. border:** Monte Baldo, without date, Nickerl leg., 1 ♂ (NMPC); same locality, without date and collector’s name, coll. K. Hanel, 3 ♂, 2 ♀ (SMTD); **Tuscany Reg.:** Florence, without date, B. Schwarzer leg., 1 ♂ (SMFD); Gaville (Gaville 3 km West Figline Valdarno), 26.iv.2003, B. Brugge leg., 1 spec. (ZMAN); Grosseto prov.: Parco Regionale della Maremma, San Rabano env., pasture, 7.iv.2003, Kment leg., 1 ♂ (JRUC); Principina a Mare (sand dune on beach), 7.iv.2003, P. Kment & M. Horsák leg., 1 ♀ (JRUC); Livorno, without date and collector’s name, coll. G. Schneider, 1 ♂ (SMNS); Monte Argentario, without date and collector’s name, 3 ♂, 3 ♀ (NHMW); Monte Pisano, without date and collector’s name, 1 ♀ (NMPC); Orbetello, v. (without year), Miyer leg., coll. W. H. Muche, 1 ♂, 1 ♀ (SMTD); Passo della Futa, without date, Klynar leg., 1 ♀ (NMPC); Piombino, iii.-iv.1908, A. Kniz leg., 4 ♂, 1 ♀ (NHMW); same locality, iii.-iv. (without year), Molitor leg., 2 ♂ (NHMW); same locality, without date and collector’s name, 1 ♂, 1 ♀ (NHMW); Pisa, 7.ix.1976, Miloš Fassati leg., 1 ♂ (JRUC); Vallombrosa, without date, O. Schneider leg., 1 ♀ (HNHM); Viareggio, without date, Hanuš leg., 1 ♂, 1 ♀ (NMPC); Volterra, iv.1976, T. Osten leg., 1 ♂, 1 ♀ (SMNS); Tuscany Reg., Firenze, 1924, Lembardi leg., ex coll. Reitter, coll. P.v.d. Wiel, 1 ♀ (ZMAN); Tuscany Reg., without specific locality: without date, Bellier leg., 2 ♂, 2 ♀ (ZSM); without date, M. Argentario leg., 4 ♀ (NHMW); Stierlin leg., general coll., 1 ♀ (SDEI); without date and collector’s name, general coll. 1 ♀ (SDEI); **Umbria Reg.:** Lake Trasimeno, without date, Klynar leg., 3 ♂, 1 ♀ (NMPC); Monte Martano, 11.-20.ix.2002, P. M. Hammond leg., coll. P. M. Hammond, 1 ♂ (BMNH); Perugia, 1050 m, 31.v.1962, Battoni leg., 1 ♂ (MHNG); **Veneto Reg.:** Ca’ Noghera, without date and collector’s name, coll. W. H. Muche, 1 ♀ (SMTD); Lido di Venezia, without date, G. Waldo leg., 1 ♀ (BMNH); Limena, without date and collector’s name, 1 ♂ (ZMHB); Padua, Abano Terme, 24.iii.1975, E. Ulbrich leg., 1 ♂ (NHMW); Verona, without date, K. Neumann leg., 1 ♀ (SMFD); Vicenza, without date, Minozzi leg., coll. W. H. Muche, 1 ♂ (SMTD); **Italy without specific locality:** 1878, Ollerich leg., coll. W. H. Muche, 1 ♂ (SMTD); without date, Bötzelmann leg., ex coll. G. Reineck, coll. Bosch, 1 ♂ (SMFD); without date, Reitter leg., 1 ♂ (NMPC); without date, Sprenger leg., 1 ♂ (NMPC); without date and collector’s name, 3 ♂, 2 ♀ (NMPC); Monte Falcone [not located], without date, J. Fleischer leg., 1 ♀ (NMPC);

**Liechtenstein:** Vaduz, without date, Dr. Feige leg., 1 ♂ (ZMHB);

**Macedonia:** Dojran Lake, 140-350 m, 1.vi.1985, Vilyer et Sruta leg., 1 ♀ (JRUC); Galičica (Mt. and National Park), 5.vi.1973, R. Grimm leg., 1 ♀ (SMNS); Jakupica (Mts.), without date, A. Matejka leg., 1 ♀ (NMPC); same locality, without date, O. Kodym leg., 3 ♂, 4 ♀ (NMPC); same locality, without date, Purkyne leg., 1 ♀ (NMPC); **Skopje Region:** Skopje, 21.iv.1961, J. Th. Skovgaard leg., 1 ♀ (ZMUC); same locality, 21.vi.1973, Blumenthal leg., 2 ♂ (SMNS); same locality, vi.1980, R. de Vos leg., 1 ♂ (ZMAN); same locality, 1985, Zeller leg., coll. W. H. Muche, 1 ♀ (SMTD); Vardar river shore in Skopje, 1.vi.1980, F. Heike leg., 2 ♂, 3 ♀ (ZMHB); **Mavrovo Reg.:** South of Mavrovo, ix.1976, Schurmann leg., 1 ♂ (NHMW); **Vardar Reg.:** Veles, without date, Smetana leg., 1 ♀ (NMPC); **Macedonia without specific locality:** without date and collector’s name, 1 ♂ (HNHM);

**Moldavia:** Bălți, 19.vi.1973, L. Zimina leg., 1 ♀ (ZMUM);

**Monaco:** without specific locality, beginning of May, W. Sattler leg., 1 ♂ (SMFD);

**Montenegro:** **Bar:** Rikavac, 1800 m, 25.vi.-2.vii.1914, Grenze Penther leg., 1 ♂ (NHMW); same locality, 1800-2000 m, 1914, Grenze Penther leg., 1 ♀ (NHMW); Sutomore Mont, 22.v.1978, G. J. Slob leg., 1 ♂ (ZMAN); **Berane:** 6.vii.1917, Akad. Balk. Exped., E. Csiki leg., 1 ♀ (HNHM); **Budva:** 5.-20.v.1954, Kuntzen leg., 1 ♂ (ZMHB); same locality, 30.iv.1975, J. Strejček leg., 1 ♀ (JRUC); same locality, without date, Hummer leg., coll. Franklin Muller, general coll., 1 ♂ (SDEI); **Podgorica:** 1900, Führer leg., 2 ♂, 1 ♀ (ZMAS); same locality, 14.vii.1910, Führer leg., 1 ♂, 1 ♀ (ZMAS); same locality, 1914, Grenze Penther leg., 1 ♀ (NHMW); same locality, without date, Führer leg., 3 ♂, 4 ♀ (ZMAS); Prokletije or Albanian Alps: Gusinje, without date, Purkyne leg., 3 ♂, 2 ♀ (NMPC); **Ulcinj:** v.1976, Marggi leg., 1 ♀ (MHNG); same locality, 8.vi.1984, J. Strejček leg., 1 ♂ (JRUC); Grenzgeb. [not located], without date, F. Gerlach leg., 1 ♂ (ZMHB); **Žabljak:** 1907 m, 15.vi.2006, L. Blažej leg., 1 ♂ (JRUC); 9.viii.1971, Drovenik leg., 1 ♀ (SMNS); Durmitor (Mt.): vii.1970, W. Sach leg., 4 ♂, 1 ♀ (NHMW); Dobri Dol, 1900 m, vii.1980, Krätschmer leg., 1 ♂ (SMNS); Jaksica Katuni [not located], 1800 m, without date, Kaszab & Szekessy leg., 1 ♀ (HNHM); Todorov Dol, without date, J. Mařan leg., 1 ♀ (NMPC); same locality, 1800 m, 2.vii.1958, Kaszab & Szekessy leg., 1 ♂ (SMNS); Štuoc, 1850 m, vii.2001, Ivan Marvan leg., traps, 2 ♂ (JRUC);

**Poland:** Tarnow forest, without date and collector’s name, 1 ♂, 1 ♀ (NHMW);

**Romania:** **Banat Reg.:** Anina, 29.iv.1986, R. Fencl leg., 2 ♀ (JRUC); Banat, without date, Reitter leg., coll. Reitter, 1 spec. (HNHM); **Black sea** (without specific location), viii.1968, Niehuis leg., 1 ♂ (SMNS); **Danube Delta river**, Babadag, 27.v.2002, P. Schule leg., 1 ♀ (SMNS); **Transsylvania Reg.:** Krassó-Szörény county, Mehádia, 22.iv.1915, J. Fodor leg., 1 ♂ (HNHM); same locality, 26.iv.1915, J. Fodor leg., 1 ♀ (HNHM); Aiud, Irk leg., 1 ♀ (NHMW); **Wallachia Reg.**, without specific locality, without date and collector’s name, coll. K. Fuss, 1 ♀ (HNHM); Mamaia, without date, Hlisnikowski leg., 1 ♂ (NMPC); Reşiţa, without date, D. Kanabe leg., coll. Kanabe, 2 ♂ (HNHM); Paraipanii [not located], 1.iv.1917, W. Liebmann leg., general coll., 1 ♀ (SDEI); same locality, 11.iv.1917, W. Liebmann leg., general coll., 1 ♂ (SDEI); Paraipanii, 29.iv.1917, W. Liebmann leg., general coll., 1 ♂ (SDEI); without specific locality, without date and collector, coll. K. Hanel, 2 ♂ (SMTD);

**Russia:** **North Caucasian Federal Dist.**, Mount Elbrus, without date and collector’s name, 3 ♀ (JRUC); same locality, without date and collector’s name, coll. E. Jagemann, 1 ♂ (MZMB); North of Mount Elbrus, Malka River, without date, Ryssel leg., 1 ♀ (ZMHB); North Ossetia (Alania), Mozdok, 14.iv.1929, coll. O. N. Kabakov, 1 ♂ (ZMAS); North of Ossetia, Skalisty Mt., drainage basin of Ardon river, 1000 m, 2.viii.1997, A. G. Koval leg., 1 ♂ (JRUC); Stavropol Kray: Pyatigorsk, vii.1904, O. Liur leg., 1 ♀ (ZMHB); Yessentuki, 26.-30.viii.1982, Gordunov & Kholina leg., 1 ♂, 2 ♀ (ZMUM); **Southern Federal Dist.**, Krasnodar Kray: Adler, 1909, G. Sumakov leg., 1 ♀ (ZMAS); Anapa, 24.vii.1982, Korotiaev leg., 1 ♂ (ZMAS); Kuban Reg., Bajalaja [not located], without date, Jüthner leg., 1 ♂, 1 ♀ (NHMW); Gelendzhik, without date, N. Vorob’ev leg., 1 ♂ (ZMAS); Maykop, 8.vi.1903, Filinchenko leg., 1 ♂ (ZMAS);

**Serbia:** Belgrade, without date and collector’s name, coll. W. H. Muche, 1 ♀ (SMTD); Belgrade, Resnik, 13.iv.1936, K. Martino leg., 1 ♀ (BMNH); Crvenka (West Bačka Dist. in Vojvodina prov.), 1981, Kouřil leg., 1 ♂ (NMPC); Dojkinci (in Pirot Dist.), 1.v.2002, T. Lackner leg., 1 ♂ (ZMAN); Niš (in Nišava Dist.), iv.1957, Lenczy leg., 2 ♀ (HNHM); Novi Pazar (in Raška Dist.), 1907, Carl Stock leg., 1 ♂ (SMFD); Subotica (North Bačka Dist. in Vojvodina prov.), 1909, Soos leg., 1 ♂ (HNHM);

**Slovakia:** **Banská Bystrica Reg.:** Donovaly, without date, Smetana leg., 1 ♂ (NMPC); same locality, S. Bílý leg., 1 ♂ (NMPC); **Košice Reg.**, Zádiel, 23.vi.1939, Balogh & Kaszab leg., 1 ♀ (HNHM); **Nitra Reg.:** Kamenný Most (in Nové Zámky Dist.), without date, Smetana leg., 1 ♂ (NMPC); Obid (in Nové Zámky Dist.), on terrace, 21.iv.2000, Mantič leg., 1 ♀ (JRUC); Parkáň [Štúrovo], without date, Baumert leg., 1 ♀ (NMPC); Štúrovo, 5.vii.1954, J. Strejček leg., 2 ♂ (JRUC); same locality, 23.v.1959, J. Strejček leg., 2 ♀ (JRUC); same locality, vii.1961, J. Strejček leg., 1 ♀ (JRUC); same locality, 17.v.1965, A. Svozil leg., 2 ♂ (SMNS); same locality, 9.v.1984, Klouček leg., 1 ♀ (JRUC); same locality, iv.1992, Krásenský leg., 1 ♀ (JRUC); same locality, without date, I. Kovář leg., 1 ♀ (NMPC); same locality, without date, Kouřil leg., 1 ♂ (NMPC); Štúrovo, Hegyfarok, 1955, Brejcha leg., 1 ♂ (MZMB); Štúrovo, Kamenica nad Hronem, 23.v.1994, Krásenský leg., 1 ♀ (JRUC); Obid (in Nové Zámky Dist.), terrace, 21.iv.2000, Mantič leg., 1 ♀ (JRUC); **Prešov Reg.**, Plaveč, Sv. Mikuláš street, without date, O. Kodym leg., 2 ♂, 1 ♀ (NMPC); **Trenčín Reg.:** Beluša, without date, V. Zoufal leg., 1 ♂ (NMPC); Trenčín, without date and collector’s name, 1 ♂ (NMPC); same locality, without date and collector’s name, 1 ♂ (NMPC); Štvrtok, without date, O. Kavan leg., 1 ♂ (NMPC); **Žilina Reg.:** Radola (in Kysucké Nové Mesto Dist.), without date and collector’s name, 1 ♂ (NMPC); Bratislava, without date, A. Hoffer leg., 1 ♀ (NMPC); same locality, without date, O. Kavan leg., 2 ♂ (NMPC); same locality, without date and collector’s name, 1 ♀ (NMPC); Gombasek Cave, without date, J. Macek leg., 1 ♀ (NMPC); Tatra Mts., 1973, J. Strejček leg., 1 ♂ (JRUC);

**Slovenia:** **Carniola Reg.:** without date and collector’s name, coll. K. Hanel, 1 ♂ (SMTD); without date, Schiodte leg., 1 ♀ (ZMUC); **Littoral Reg.:** Kozina, Petrinje, 29.iv.-1.v.2003, J. Vávra leg., 1 ♀ (JRUC); Podgorski kras, Petrinje, 1.vii.2002, Z. Malinka leg., 1 ♀ (JRUC); Strunjan, vi.1881, Zigeuner leg., ex coll. Winkler, 1 ♀ (MHNG); Tolmin, without date, coll. V. Zoufal, 1 ♂ (MZMB); same locality, Wagner leg., 1 ♀ (NHMW); Wippach [Vipava], without date and collector’s name, coll. Kaufmann, 2 ♂ (NHMW); **Lower Carniola Reg.:** Novegomesto or Novo Mesto, without date, Adoha leg., 1 ♀ (NMPC); **Slovenian Littoral Reg.:** Izola, 4.vi.1968, M. Hubes leg., 1 ♂ (SMNS); Piran (bay), 22.viii.1989, U. Göllner leg., 1 ♀ (ZMHB); **Upper Carniola Reg.:** Bled, vi.1911, V. Zoufal leg., coll. K. Mazura, 1 ♂ (MZMB); same locality, without date, Prochazka leg., coll. Prochazka, 1 ♂ (NMPC); Velika Planina, v.-vii.1912, Ramme leg., 1 ♂, 1 ♀ (ZMHB); same locality, without date, Ramme leg., 1 ♂ (ZMHB);

**Spain:** **Andalusia:** Huelva prov., Almonte, v.1969, C. Konig leg., 1 ♀ (SMNS); without more specific locality, without date, Witt leg., 1 ♀ (SDEI); **Aragon**, Turia River, without date and collector’s name, coll. F. Speiser, 1 ♀ (HNHM); **Asturias**, Tuilla, 22.v.1973, K. W. Harde leg., 1 ♀ (SMNS); **Basque Country**, San Sebastián, without date, J. Ardois leg., 1 ♂, 2 ♀ (MNCN); **Catalonia:** Barcelona, Sabadell, iv.1974, Krätschmer leg., 1 ♀ (SMNS); same locality, 15.vi.1977, without collector’s name, coll. Poul Kry Poulsen leg., 1 ♀ (ZSM); Bagur, 15.-28.vi.1957, R. Schrepfer leg., coll. R. Schrepfer, 1 ♀ (SMNS); Costa Brava: Castell-Platja, d’Aro, 16.v.1981, K. W. Harde leg., 1 ♂ (SMNS); Empúries, 22.v.1973, K. W. Harde leg., 1 ♀ (SMNS); same locality, 18.x.1974, K. W. Harde leg., 4 ♂, 8 ♀ (SMNS); same locality, 22.v.1973, R. Köstlin leg., coll. R. Köstlin, 1 ♀ (SMNS); same locality, iv.1982, W. G. Ullrich leg., 2 ♀ (MHNG); same locality, without date and collector’s name, 1 ♂, 1 ♀ (MNCN); L’Escala, 14.v.1975, Heikel leg., 1 ♀ (SMNS); L’Estartit, 9.v.1973, K. W. Harde leg., 1 ♂ (SMNS); same locality, 16.v.1973, K. W. Harde leg., 1 ♀ (SMNS); same locality, 16.v.1973, R. Köstlin leg., coll. R. Köstlin, 1 ♀ (SMNS); same locality, 11.iv.1977, K. W. Harde leg., 1 ♀ (SMNS); same locality, 14.v.1980, E. Ulbrich leg., 1 ♂ (NHMW); Lloret de Mar, 24.-25.iii.1985, J. Frisch leg., 2 ♂ (ZMHB); Palamós, 12.vi.1957, without collector, 1 ♀ (ZMHB); same locality, 17.v.1957, without collector, 1 ♂ (ZMHB); same locality, 20.vi.1957, without collector, 1 ♀ (ZMHB); same locality, 7., 19.ix.1959, without collector, 2 ♂ (ZMHB); same locality, Girona prov., 7.-13.ix.1974, Ulbrich leg., coll. Ulbrich, 1 ♂ (SMNS); Palamós, La Fosca, 4., 20.v.1975, C. v. Nidek leg., 1 ♂ (ZMAN); Pyrénées (Mts.), Espot, 2200 m, vi.-vii. (without year), Umlauf leg., 1 ♂ (SMNS); Pyrénées (Mts.), La Jonquera, 26.v.1980, E.Ulbrich leg., 1 ♀ (SMNS); Roses, 16.vi.1869, R. V. Budberg leg., 1 ♂ (NHMW); same locality, vi.1966, Budberg leg., 1 ♀ (NHMW); same locality, vi.1967, Budberg leg., 1 ♂ (NHMW); same locality, v., vi.1968, R. V. Budberg leg., 2 ♀ (NHMW); same locality, 1.vii.1970, R.V. Budberg leg., 1 ♀ (NHMW); same locality, 20.vi.1971, R. V. Budberg leg., 1 ♂ (NHMW); same locality, 2.iv.1974, W. Ziegler leg., coll. W. G. Ullrich, 1 ♀ (MHNG); Toroella de Montgri, 7.viii.1972, C. Koening leg., 1 ♀ (SMNS); **Galicia**, without date, Lauffer leg., 1 ♀ (MNCN); **Ibiza island**, San Rafael, without date, M. Escalera leg., 1 ♂ (MNCN); **Valencia:** without date, G. Reineck leg., coll. Bosch, 1 ♂ (SMFD); same locality, 2.iv.[19]74, W. Ziegler leg., coll. W. G. Ullrich, 1 ♂ (MHNG); Oliva prov., 3.iv.1974, W. Ziegler leg., coll. W. G. Ullrich, 1 ♀ (MHNG);

**Switzerland:** **Aargau Canton:** Bözen, vi.1898, A. Weis leg., 1 ♀ (SMFD); Kaiserstuhl, vi.1952, R. Papperitz leg., 1 ♂ (SMNS); **Bern Canton:** Biel (Bienne), Hartmann leg., 1 ♀ (NMPC); Kandersteg, 16.vii.(without year), Stock leg., 1 ♀ (SMFD); Isenfluh, Bernese Oberland (Bernese Highlands), 2.viii.1963, R. Köstlin leg., 1 ♀ (SMNS); **Vaud Canton:** Bex, 27.vi.1904, without collector’s name, 1 ♀ (BMNH); same locality, 3.v.1904, without collector’s name, 1 ♀ (BMNH); same locality, 1904, without collector’s name, 1 ♀ (BMNH); Lausanne, 3.viii.1985, Hoop leg., 1 ♂ (ZMAN); Vallorbe, without date, J. Fleischer leg., 1 ♀ (NMPC); **Switzerland without specific locality:** without specific locality, without date, Kirsch leg., 2 ♂, 1 ♀ (SMTD); without specific locality, without date and collector’s name, 2 ♂ (SMFD); Waldhaus [not located], 8.vi.1919, without collector’s name, 2 ♂, 1 spec. (ZMHB);

**Turkey:** **Aegean Reg.:** Izmir, Gümüldür, without date and collector’s name, 1 ♂ (NMPC); same locality, 18.-31.iii.1917, La Baume leg., 3 ♂, 1 ♀ (ZMHB); Ödemis (40 km North Kastamonu), 1000 m, 11.vii.1986, A. Korell leg., 1 ♂ (SMNS); **Black Sea Reg.:** Abant lake, vi.1983, G. Sama leg., 1 ♀ (SMNS); Trabzon, without date, Beyrolle leg., coll. Sharp, 1 ♀ (BMNH); **Bosphorus strait**, Dopot Garten [not located], without date, Dr. J. Jaus leg., 1 ♀ (NHMW); **Ionia Reg.:** Ephesus, v.1904, Dr. Werner leg., 1 ♂ (NHMW); **Marmara Reg.:** Çanakkale prov., Gelibolu, 10 m, 3.v.1982, without collector’s name, 1 ♂, 1 ♀ (MHNG); Edirne, Necatiye, 1.iv.1990, A. Podlussany leg., 1 ♂ (HNHM); Edirne, v.1894, Flach leg., 1 ♂ (SMFD); same locality, 16.v.1993, P. Průdek leg., 1 ♂ (JRUC); same locality, 3.vii.1993, Kantner leg., 1 ♀ (JRUC); same locality, 16.v.1993, P. Průdek leg., 1 ♂ (JRUC); İpsala, 2.iv.1980, without collector’s name, 1 ♂ (SMNS); Istanbul, Ispartakule, 7.iv.1908, Bodemeyer leg., 1 ♀ (ZMHB); Istanbul, Kilyos (in Sarıyer Dist.), beach zone, 1964, Schweiger leg., 1 ♀ (SMFD); Istanbul, Yeşilköy, 1964, Schweiger leg., 2 ♀ (SMFD); Istanbul, Küçükçekmece, 1965, Schweiger leg., 1 ♀ (SMFD); Istanbul, 1900, J. Fodor leg., 1 ♂ (HNHM); same locality, 1900, without collector’s name, coll. J. Fodor, 1 ♀ (HNHM); same locality, 7.iv.1908, Bodemeyer leg., coll. Piesbergen, 1 ♂ (SMNS); same locality, 10.-11.v.1937, without collector’s name, 1 ♂ (HNHM); same locality, 31.iii.1977, Scheuern leg., 2 ♀ (SMNS); same locality, 2. iv.1999, Orszulik leg., 1 ♂, 1 ♀ (JRUC); same locality, 22.v.2001, Orszulik leg., 1 ♂ (JRUC); same locality, without date, S. Stelano, Wimmer leg., 1 ♂, 1 ♀ (NHMW); Lüleburgaz (in East Thrace), 21.iii.1972, Bremer leg., 1 ♂ (SMNS); Tekirdağ (Rodosto, hist. name), 24.vii.1939, Vasvati leg., 1 ♀ (HNHM); Üsküdar, without date and collector’s name, coll. Winkler, 2 ♂ (NHMW); **Southeastern Anatolia Reg.:** without date and collector’s name, 1 ♂, 1 ♀ (BMNH); Edessa (West) in Şanlıurfa, 17.iv.1980, Krätschmer leg., 1 ♀ (SMNS); Kodi, without date and collector’s name, 1 ♀ (ZMHB);

**United Kingdom:** Binea [not located], without date and collector’s name, 1 ♀ (BMNH); Cornwall, Whitesand Bay, Land’s End, 1935, G. C. Champion & H. C. Champion leg., coll. G. C. Champion, 1 ♀ (BMNH); Darneth, without collector’s name, 1.v.1870, R. O. S. Clarke leg., in chalk pit, 1 ♂, 1 ♀ (BMNH); Rochester, 18.iv.1894, without collector’s name, 1 ♂ (ZMUC);

**Ukraine:** **Crimea:** Alma River, without date, Rivakov leg., 2 ♂, 4 ♀ (ZMAS); same locality, 25.iv.1954, Kasheev leg., coll. O. N. Kabakov, 2 ♂ (ZMAS); Foros, vi.1930, without collector’s name, 1 ♀ (ZMUM); Kherson, 6.-16.iv.1903, Ewert leg., 1 ♀ (ZMHB); Kyzyl-Tash (Krasnokamyanka), 15.vii.1911, Pliginski leg., 1 ♀ (ZMAS); Mama-Chakrak, Kertch, 26.iv.1911, A. Dirin leg., 1 ♂ (ZMAS); Odessa, without date and collector’s name, coll. Schwarzer, 1 ♂ (SMFD); same locality, Limanskaya, 11.v.1979, Kabakov leg., coll. O. N. Kabakov, 1 ♀ (ZMAS); Sevastopol, 4.-8.iv.1899, Birulia leg., 2 ♀ (ZMAS); same locality, 10.iv.1911, V. Kizeritskiy leg., 1 ♂ (ZMAS); same locality, 26.iii.1912, N. L. Pastukhov leg., 1 ♀ (ZMAS); same locality, 6.vi.1941, L. Arnol’di leg., 1 ♀ (ZMUM); same locality, without date, coll. W. H. Muche, 2 ♂ (SMTD); Simferopol, 6.iv.1908, O. G. & K. Khristoforovy leg., 1 ♂ (ZMAS); same locality, 20.iv.1908, O. G. & K. Khristoforovy leg., 1 ♀ (ZMAS); Tarkhankut, 9.v.1980, Mimonov leg., 1 ♂ (ZMUM); Taushan-bazar, 29.vi.1907, B. Grigoriev leg., 1 ♀ (ZMAS); same locality, Jaltinsk [Zaliv Yaltinskiy], 26.vi.1907, W. Pliginkiy leg., 1 spec. (ZMAS); Simferopol, without date and collector’s name, 1 ♂, 1 ♀ (NHMW); without specific locality, without date and collector’s name, 1 ♂, 1 ♀ (HNHM); Yalta, 21.v.1925, without collector’s name, 1 ♂ (ZMAS); Yarlygachs [not located], 13.v.1913, Aleksandov leg., 1 ♂ (ZMAS); Yevpatoria (Eupatoria); viii.1972, S. Alekseev leg., 1 ♂ (ZMUM); **Lviv Oblast**, Galicia, Gródek Jag.[ielloński] (Horodok), without date, D. Grabowski leg., coll. Grabowski, 1 ♀ (HNHM); **Zaporizhia Oblast**, Kamianka-Dniprovska, 5.vii.1973, Koval leg., coll. O. N. Kabakov, 1 ♂ (ZMAS).

**Not located:** Obercugalin, without date and collector’s name, coll. Piesbergen, 1 ♀ (SMNS); Waldhann, 1978, without collector’s name, coll. Piesbergen, 2 ♂ (SMNS);

### Ablattaria subtriangula

**Spain:** **Álava prov.**, Nanclares, vi.1966, J. Vives leg., 1 ♂, 1 ♀ (MNHN); **Biscay prov.:** Orduña, iv.1881, Kobelt leg., 1 ♀ (SMNS); same locality, without date, Simon leg., 1 ♂ (MNHN); **Burgos prov.:** Puerto de Orduña, 10.v.1992, J. Alcorta leg., 1 ♀ (MNCN); Soncillo (Valle de Valdebezana), 22.iv.-20.v.1964, I. & E. Yarrow leg., 1 ♂ (BMNH); **Cantabria prov.:** Reinosa, vi.1902, G. Schramm leg., 1 ♀ (MNCN); same locality, without date and collector’s name, 1 ♂ (MNCN); Soto, Mr. Martin leg., 3 ♂, 1 ♀ (ZFMK); Tortosa, without date and collector’s name, 1 ♀ (MNCN); **La Rioja prov.**, Cameros (La comarca de Tierra de Cameros), without date, C. Bolivar leg., 1 ♀ (MNCN).
